# Tuning Active Hydrogen on Reconstructed RuO_2_/Co(OH)_2_ Catalysts for Selective Ammonia Synthesis

**DOI:** 10.1002/adma.202515346

**Published:** 2025-09-25

**Authors:** Anquan Zhu, Heng Liu, Lulu Qiao, Bin Liu, Kunlun Liu, Chuhao Luan, Kai Liu, Yin Zhou, Dewu Lin, Guoqiang Gan, Jiapei Li, Guo Hong, Wenjun Zhang

**Affiliations:** ^1^ Department of Materials Science and Engineering & Center of Super‐Diamond and Advanced Films City University of Hong Kong 83 Tat Chee Avenue Kowloon Hong Kong P. R. China; ^2^ Advanced Institute for Materials Research (WPI‐AIMR) Tohoku University Sendai 980‐8577 Japan; ^3^ State Key Laboratory of Powder Metallurgy Central South University Changsha 410083 P. R. China; ^4^ Shenzhen Key Laboratory of Micro/Nano‐Porous Functional Materials (SKLPM) Department of Chemistry Department of Materials Science and Engineering and SUSTech‐Kyoto University Advanced Energy Materials Joint Innovation Laboratory (SKAEM‐JIL) Southern University of Science and Technology (SUSTech) Shenzhen 518055 P. R. China; ^5^ State Key Laboratory of Chemical Resource Engineering Beijing Advanced Innovation Center for Soft Matter Science and Engineering College of Chemistry Beijing University of Chemical Technology Beijing 100029 P. R. China; ^6^ State Key Laboratory of Advanced Waterproof Materials School of Advanced Materials Peking University Shenzhen Graduate School Shenzhen 518055 P. R. China; ^7^ Key Laboratory of Subsurface Hydrology and Ecological Effects in Arid Region Ministry of Education School of Water and Environment Changan University Xi'an 710064 P. R. China; ^8^ The Shenzhen Research Institute City University of Hong Kong Shenzhen 518057 P. R. China

**Keywords:** active hydrogen, ammonia synthesis, electrolyte optimization, precatalyst reconstruction, rechargeable Zn‐NO_3_^−^ battery

## Abstract

The electrochemical nitrate reduction reaction (eNO_3_RR) is widely recognized as a promising strategy for sustainable ammonia production and supporting the nitrogen cycle. However, its advancement is impeded by complex behavior of reaction intermediates and the inevitable reconstruction of precatalysts. To address these challenges, the generation and utilization of active hydrogen (*H) are strategically managed by tailoring the RuO_2_/Co_3_O_4_ precatalyst and optimizing the electrolyte composition (OH^−^ and NO_3_^−^ concentration), thereby selectively enhancing ammonia formation. Consequently, the in situ reconstructed RuO_2_/Co(OH)_2_ catalyst achieves an impressive ammonia yield of 35.9 ± 0.9 mg h^−1^ cm^−2^ and a Faradaic efficiency (FE) of 98.1 ± 2.6% at −0.3 V versus RHE. Furthermore, the catalyst shows significant potential for applications in nitrate‐rich wastewater treatment and rechargeable Zn‐NO_3_^−^ batteries, maintaining stable operation for over 260 hours at 1 mA cm^−2^ with only a 6 mV increase in the potential window. Mechanistic studies reveal that electron‐rich RuO_2_ facilitates *H generation through water dissociation, which subsequently migrates to Co(OH)_2_ to hydrogenate nitrogenous intermediates, selectively producing ammonia. This study highlights the importance of designing efficient catalytic systems that address both precatalyst reconstruction and the complexities of reactant and intermediate conversion in electrolytes, which are essential for managing the intricate electron and proton transfer processes involved in eNO_3_RR.

## Graphical abstract

A combined approach of precatalyst design and electrolyte engineering is employed to develop highly active, selective, and stable catalysts for electrochemical nitrate reduction. In alkaline media, the reconstructed RuO_2_/Co(OH)_2_ catalyst demonstrates impressive performance in ammonia synthesis, rechargeable Zn‐NO_3_^−^ battery, and the remediation of nitrate‐containing wastewater.

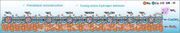

## 1 Introduction

Ammonia is widely used in agriculture and various industries, including fertilizers, explosives, refrigeration, textiles, and pharmaceuticals.^[^ ^1^ ^]^ Recently, it has gained intensive attention in energy storage and transportation due to its high energy density (3 kWh L^−1^) and zero‐pollution combustion products (nitrogen and water), contributing to a low‐carbon renewable energy future.^[^ ^2^ ^]^ However, current ammonia synthesis primarily relies on the Haber‐Bosch (H‐B) process, an energy‐intensive method that accounts for 1.8% of the globe's annual carbon emissions, conflicting with the net‐zero carbon goals.^[^ ^3^ ^]^ The search for new, clean, and sustainable ammonia synthesis methods has led to the exploration of the electrochemical nitrogen reduction reaction (eN_2_RR), which utilizes the abundant nitrogen from the air. Yet, its practical application is hindered by inefficiency primarily stemming from the high dissociation energy of the N≡N bond (941 kJ mol^−1^) and the low solubility of nitrogen gas in water (0.019 g L^−1^ at 1 atm and 25 °C). In response to these challenges, the electrocatalytic nitrate reduction reaction (eNO_3_RR) has gained prominence, mainly due to the lower dissociation energy of the N = O bond (204 kJ mol^−1^) and the significantly higher solubility of nitrate ions (880 g L^−1^).^[^ ^4^ ^]^ However, a primary concern regarding the eNO_3_RR is the sustainable supply of nitrate. Compared to supply strategies involving plasma activation of N_2_ and O_2_ gases or electrochemical oxidation of N_2_, the separation and purification of nitrate from various wastewater sources, such as industrial, domestic, and nuclear wastewater, offer significant advantages in terms of energy consumption and purity. The abundance of these nitrate resources establishes a solid basis for the eNO_3_RR, given that the designed catalysts can function efficiently in the presence of coexisting inorganic and organic contaminants.^[^ ^5^ ^]^ This capacity would ultimately facilitate the nitrogen cycle in nature.^[^ ^6^ ^]^

Theoretically, eNO_3_RR involves complex deoxygenation and hydrogenation steps, which result in sluggish reaction kinetics.^[^ ^7^ ^]^ To enhance ion transport and electron transfer, alkaline solutions such as KOH are often used as electrolytes. The conversion of NO_3_^−^ to NH_3_ can proceed via two pathways: Path 1: NO_3_^−^→*NO_3_→*NO_3_H→*NO_2_→*NO_2_H→*NO→*NOH→*N→*NH→*NH_2_→*NH_3_→*+NH_3_(g); or Path 2: NO_3_^−^→*NO_3_→*NO_3_H→*NO_2_→*NO_2_H→*NO→*NOH→*HNOH→*H_2_NOH→*NH_2_→*NH_3_→*+NH_3_(g).^[^ ^2b^ ^]^ Both pathways primarily involve two types of intermediates: nitrogenous groups and active hydrogen species (*H). Each transition step of the nitrogenous groups requires the participation of active hydrogen; insufficient *H will halt the conversion, leading to the release of intermediates like *NO_2_ into the electrolyte as the NO_2_^−^ byproduct.^[^ ^8^ ^]^ Conversely, excessive *H may recombine to form H_2_.^[^ ^9^ ^]^ Therefore, this competitive behavior highlights the crucial demand for rational catalyst design for eNO_3_RR, aimed at manipulating intermediate dynamics in a balanced manner.

Recently, Lu et al. demonstrated that PdCu achieved the highest ammonia yield and FE among the prepared PdM (M = Fe, Co, Ni, Cu) bimetallene due to its moderate adsorption ability for N‐species (*NO_3_ and *NO_2_), enhanced *NO activation, and reduced hydrogen evolution reaction (HER) activity.^[^ ^10^ ^]^ Xiong et al. proposed that, in a developed Ni_3_N nanosheet with Cu nanoclusters, the *H generated from water dissociation on Ni_3_N rapidly transferred to Cu through interfacial Ni−N−Cu bonds, facilitating hydrogenation for ammonia formation on Cu.^[^ ^11^ ^]^ These strategies, which emphasize the compositional and structural design of catalysts, offer valuable insights for the development of eNO_3_RR catalysts. However, it is important to note that these eNO_3_RR catalysts often undergo dynamic reconstruction (e.g., morphology, structure, composition, or phase changes) under working conditions.^[^ ^4^, ^12^ ^]^ As a result, the pre‐defined composition and structure of the as‐synthesized catalysts are no longer relevant to the observed electrochemical performance. For instance, Beatriz et al. found that Cu_2_O nanoparticles tended to convert into a Cu_2_O/Cu composite and eventually to metallic Cu at 0.1 V versus RHE and −0.3 V versus RHE, respectively, during eNO_3_RR.^[^ ^3a^ ^]^ Similarly, Cho et al. discovered that copper‐cobalt hydroxy double salt underwent in situ reconstruction into a Cu/Co(OH)_2_ composite during the same process.^[^ ^13^ ^]^ Understanding the reconstruction process and the resulting stabilized structure‐performance relationships is essential yet challenging in the design of an efficient catalyst and exploring its catalytic pathways/mechanisms.^[^ ^14^ ^]^

Previously, we observed that the eNO_3_RR kinetics of reconstructed Co(OH)_2_ catalysts derived from Co_3_O_4_ were inhibited by the adsorption‐energy scaling relations of reaction intermediates on single active component, as well as by an insufficient supply of active hydrogen.^[^ ^4a^ ^]^ In this study, we aim to improve ammonia yield while maintaining high selectivity through strategic design of precatalysts. This approach involves comprising Co_3_O_4_ octahedra with a controlled loading of RuO_2_ nanoparticles. The resulting RuO_2_/Co_3_O_4_ precatalysts evolve into RuO_2_/Co(OH)_2_ catalysts upon reaction. Comprehensive experimental and theoretical analyses reveal that the *H species generation is precisely controlled by modifying the structure and composition of precatalysts. Specifically, *H species are produced on RuO_2_ and subsequently transferred to the in situ reconstructed Co(OH)_2_, facilitating the hydrogenation of nitrogenous groups and significantly enhancing the eNO_3_RR kinetics. The optimized RuO_2_/Co(OH)_2_ catalyst achieves an NH_3_ yield of 35.9 ± 0.9 mg h^−1^ cm^−2^ and a FE of 98.1 ± 2.6% at −0.3 V versus RHE. It effectively reduces the nitrate concentrations in alkaline wastewater to below 10 ppm, well below the World Health Organization (WHO) safety threshold for drinking water (≤50 ppm). Furthermore, it maintains a high ammonia FE of 96.3 ± 3.0% after 100 hours of continuous operation. This notable stability extends to its performance in a rechargeable Zn‐NO_3_^−^ battery, which operates stably for over 260 hours at 1 mA cm^−2^. In addition to precatalyst design, we also identify that the electrolyte composition (OH^−^ and NO_3_^−^ concentrations) influences the balance between active hydrogen generation from water dissociation and its utilization in the hydrogenation of nitrogenous groups. An optimal electrolyte configuration for the RuO_2_/Co(OH)_2_ catalyst is identified as 1 m KOH and 0.1 m KNO_3_. These results underscore the importance of optimizing the intermediate behaviors through concurrently tailoring precatalysts and modifying electrolyte composition to develop highly active, selective, and stable catalysts for complex hydrogenation reactions.

## 2 Results and Discussion

### 2.1 Synthesis and Characterization of Precatalysts

**Figure 1:**
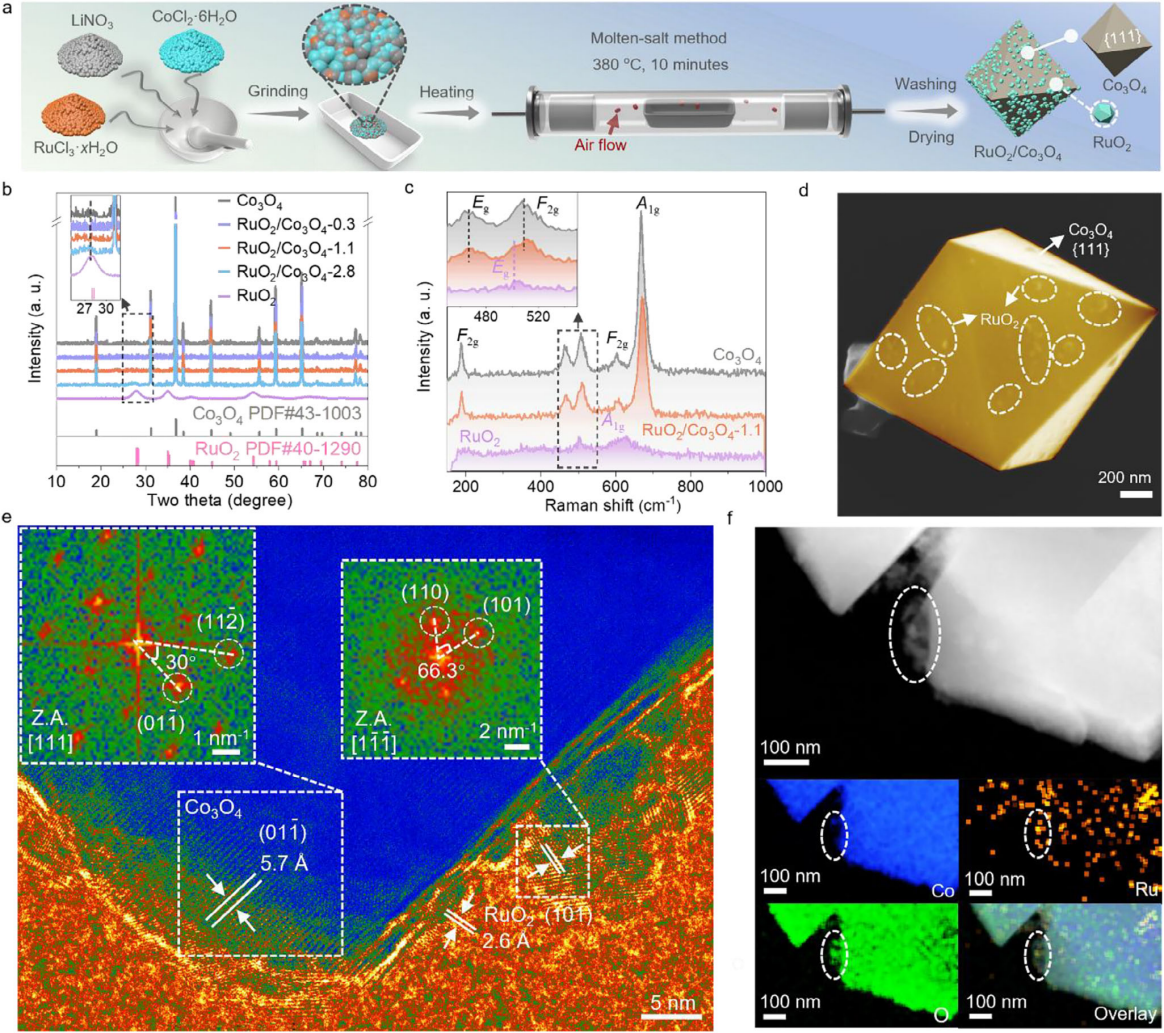
Characterizations of precatalysts. a) Synthetic route of RuO_2_/Co_3_O_4_ precatalysts. b) XRD patterns and c) Raman spectra of the RuO_2_, RuO_2_/Co_3_O_4_, and Co_3_O_4_ precatalysts. d) SEM image, e) HRTEM image (insets show the corresponding FFT patterns of Co_3_O_4_ and RuO_2_ at the marked regions), and f) high‐angle angular dark field scanning transmission microscopy (HAADF‐STEM) image and the corresponding elemental mapping of Co, Ru, and O in the RuO_2_/Co_3_O_4_ precatalyst.

A facile and effective molten‐salt method was employed to synthesize RuO_2_, Co_3_O_4_, and RuO_2_/Co_3_O_4_ composite (**Figure** **1**a). Three RuO_2_/Co_3_O_4_ composite samples with Ru/Co molar ratios of 0.3%, 1.1%, and 2.8% as identified by inductively coupled plasma optical emission spectrometry (ICP‐OES) were prepared (Table S1, Supporting Information). Accordingly, these composites are denoted as RuO_2_/Co_3_O_4_‐0.3, RuO_2_/Co_3_O_4_‐1.1, and RuO_2_/Co_3_O_4_‐2.8, respectively. As revealed by the x‐ray diffraction (XRD) patterns in Figure 1b, Co_3_O_4_ shows a cubic structure (PDF#43‐1003, FD‐3m(227)) while RuO_2_ exhibits a tetragonal structure (PDF#40‐1290, P42/mnm (136)).^[^ ^15^ ^]^ As the Ru/Co ratio increases, the (110) peak of RuO_2_ becomes more pronounced, indicating an increased RuO_2_ content. Raman analysis reveals typical vibrations of spinel Co_3_O_4_ (*A*_1g_, *F*_2g_, and *E*_g_) and a shoulder peak at ≈505 cm^−1^ corresponding to the *E*_g_ mode of RuO_2_ (Figure 1c).^[^ ^16^ ^]^ The high‐temperature molten‐salt environment promotes rapid growth and nucleation of metal oxides, leading to the formation of Co_3_O_4_ octahedron with {111} facets and irregular RuO_2_ nanoparticles (Figure S1, Supporting Information). In the RuO_2_/Co_3_O_4_ composites, RuO_2_ nanoparticles are attached to the {111} surfaces of Co_3_O_4_ octahedra, as demonstrated by scanning electron microscopy (SEM), high‐resolution transmission electron microscopy (HRTEM), and x‐ray photoelectron spectroscopy (XPS) results (Figure S2, Supporting Information). A close observation in Figure 1e reveals distinct *d*‐spacings of 5.7 and 2.6 Å, corresponding to the (01$\bar{1}$) and (101) planes of Co_3_O_4_ and RuO_2_, respectively. The corresponding fast Fourier transform (FFT) pattern displays the (01$\bar{1}$) and (11$\bar{2}$) planes with a vector angle of 30°, aligned with the [111] zone axis, indicating that the surfaces of the Co_3_O_4_ octahedra are enclosed by {111} facets. Moreover, the RuO_2_ nanoparticles exhibit (110) and (101) planes with a vector angle of 66.3°, observed along the [1$\bar{1}\bar{1}$] direction. These results confirm the successful incorporation of RuO_2_ nanoparticles onto the {111} surfaces of Co_3_O_4_. Additionally, the uniform distribution of the Ru element across the RuO_2_/Co_3_O_4_ matrix further validates the even dispersion of RuO_2_ nanoparticles on Co_3_O_4_ (Figure 1f).

### 2.2 Reconstruction of Precatalysts

**Figure 2:**
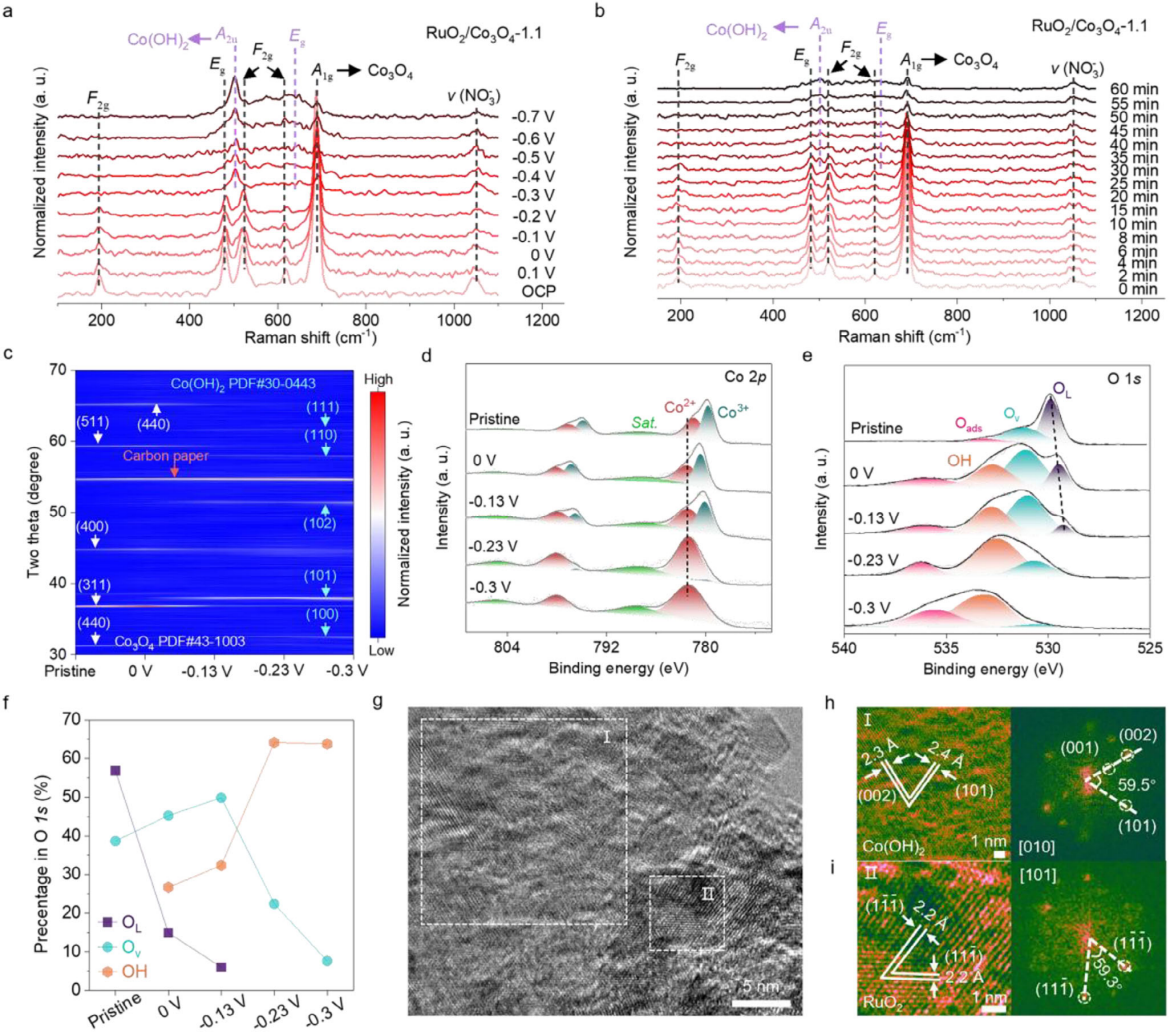
Understanding the precatalyst reconstruction. a) Potential‐dependent in situ Raman spectra of the RuO_2_/Co_3_O_4_‐1.1 composite in 1 m KOH containing 100 mm NO_3_^−^; potential ranges between 0.1 and −0.7 V with an amplitude of −0.1 V. b) Time‐dependent in situ Raman spectra of RuO_2_/Co_3_O_4_‐1.1 composite at −0.3 V in 1 m KOH containing 100 mm NO_3_^−^. Potential‐dependent c) ex situ XRD patterns, high‐resolution x‐ray photoelectron spectroscopy (HRXPS) of d) Co 2*p*, e) O 1*s*, and f) the relative content of various O species in O 1*s* of the RuO_2_/Co_3_O_4_‐1.1 composite after being treated at various potentials. g–i) HRTEM image and the corresponding fast Fourier transform (FFT) patterns of the RuO_2_/Co_3_O_4_‐1.1 composite after the reconstruction at −0.3 V versus RHE for 1 hour.

In situ Raman spectroscopy was used to monitor the surface transformations of various precatalysts.^[^ ^17^ ^]^ During eNO_3_RR, the RuO_2_/Co_3_O_4_‐1.1 composite exhibits a similar transformation rule from Co_3_O_4_ to Co(OH)_2_ as the Co_3_O_4_ sample, but the threshold voltage for phase change is reduced to −0.3 V (**Figure** **2**a; Figures S3 and S4, Supporting Information). Due to the low RuO_2_ content and liquid environment, Raman signals for RuO_2_ in the RuO_2_/Co_3_O_4_‐1.1 composite were undetectable. Time‐dependent in situ Raman spectra at −0.3 V for RuO_2_/Co_3_O_4_‐1.1 and Co_3_O_4_ show a gradual weakening of Co_3_O_4_ vibrations (Figure 2b; Figure S5a, Supporting Information). After 30 minutes, the *A*_2u_ and *E*_g_ modes of Co(OH)_2_ were observed for the RuO_2_/Co_3_O_4_‐1.1 composite.^[^ ^18^ ^]^ When extended to 1 hour, the Raman vibrations for *A*_1g_, *A*_2u_, and *E*_g_ modes of Co(OH)_2_ became independent of the applied potentials (Figure S6, Supporting Information), indicating that Co(OH)_2_ is an electrochemically stable phase for eNO_3_RR in alkaline media. In contrast to Co_3_O_4_, RuO_2_ maintains its characteristic *E*_g_ (512 cm^−1^) and *A*_1g_ (648 cm^−1^) vibrations,^[^ ^19^ ^]^ demonstrating structural stability during eNO_3_RR (Figure S5b, Supporting Information).

The phase components of the reconstructed catalysts were further analyzed using XRD. After 1 hour of electrochemical reconstruction (Figure 2c), the intensity of the Co_3_O_4_ lattice in RuO_2_/Co_3_O_4_‐1.1 decreased with the increasing negative potential, while the XRD peaks of Co(OH)_2_ became more pronounced. This illustrates that the phase change from Co_3_O_4_ to Co(OH)_2_ is potential‐dependent. A more negative potential results in a severe collapse of the Co_3_O_4_ octahedron host and an increased presence of Co(OH)_2_ nanosheets on the Co_3_O_4_ surface (Figure S7, Supporting Information). At −0.3 V, the Co_3_O_4_ surface is predominantly covered with Co(OH)_2_, consistent with the XRD results. Similar changes are observed for the pristine Co_3_O_4_, whereas RuO_2_ maintains its phase and nanoparticle morphology (Figures S8 and S9, Supporting Information). The chemical compositions of the reconstructed catalyst surface at various potentials were investigated using XPS (Figure 2d,e). The population of Co^3+^ in Co_3_O_4_ decreases with the increased negative potential, with Co^2+^ predominating in the reconstructed catalyst at −0.3 V. O 1*s* spectra reveal reduced levels of lattice oxygen (O_L_) from Co_3_O_4_, and oxygen vacancy (O_v_) and an increased hydroxide (OH) content, confirming the surface transition from Co_3_O_4_ to Co(OH)_2_.^[^ ^20^ ^]^ Detailed microstructure observations by TEM further reveal that, following reconstruction, Co_3_O_4_ octahedrons transform to Co(OH)_2_ nanosheets, while RuO_2_ maintains its nanoparticle structure with slight agglomeration (Figures S8–S12, Supporting Information). The reconstructed RuO_2_/Co(OH)_2_ (R‐RuO_2_/Co(OH)_2_) comprises Co(OH)_2_ nanosheets loading with RuO_2_ nanoparticles, as indicated by HRTEM images (Figure 2g). Distinct *d*‐spacings of 2.3 and 2.4 Å, with a vector angle of 59.5°, are assigned to the (002) and (101) lattice planes of Co(OH)_2_, respectively (zone axis: [010]). Meanwhile, RuO_2_ nanoparticle is revealed as (11$\bar{1}$) and (1$\bar{1}\bar{1}$) planes with a vector angle of 59.3° (zone axis: [101]), as shown in Figure 2h,i.

### 2.3 Electronic Structure Characterization

**Figure 3:**
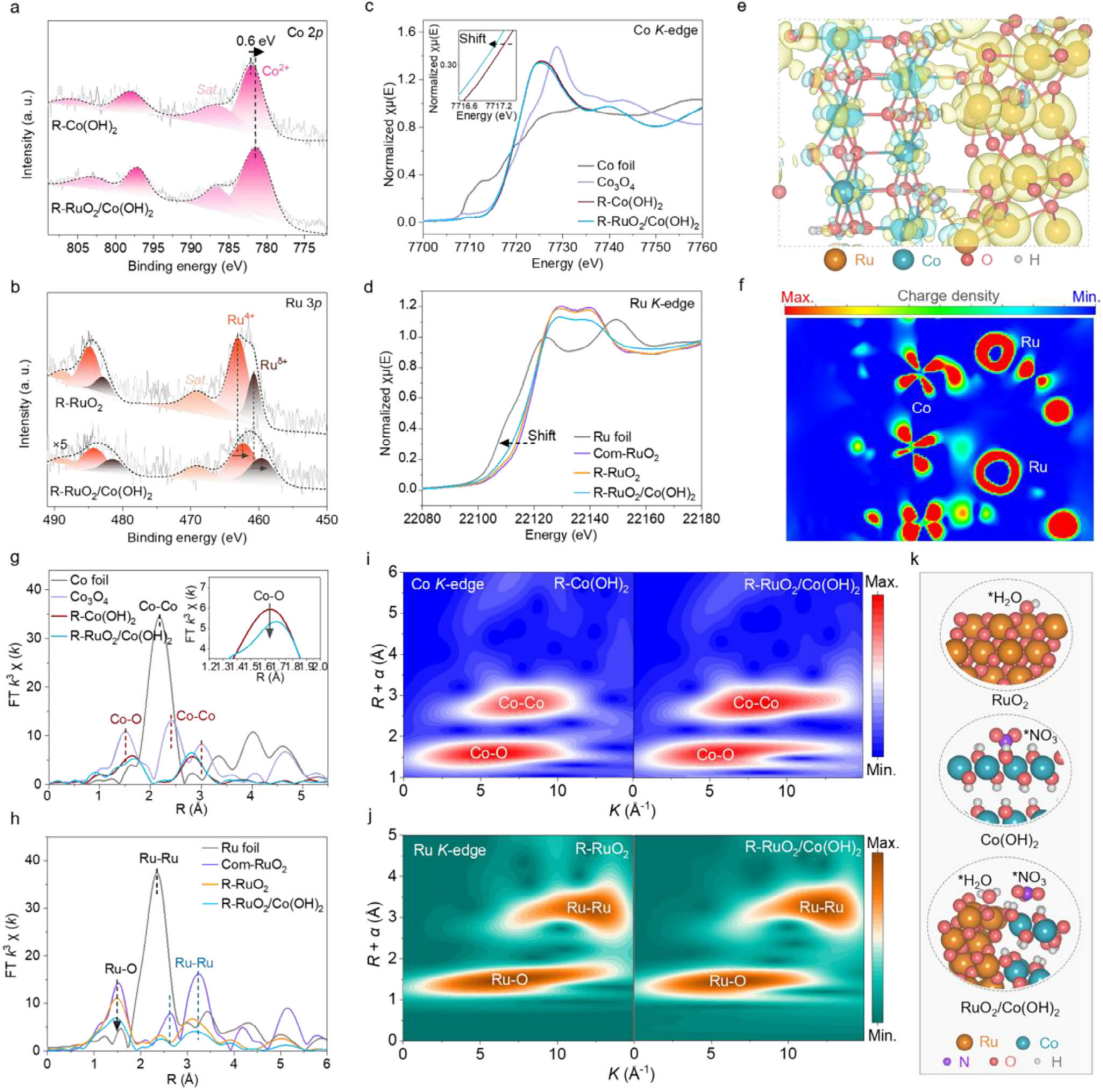
Electronic structure of the reconstructed catalysts. High‐resolution XPS spectra of a) Co 2*p* and b) Ru 3*p* in R‐Co(OH)_2_, R‐RuO_2_@Co(OH)_2_, and R‐RuO_2_. c) Co *K*‐edge and d) Ru *K*‐edge XANES spectra of the catalysts, with reference samples for comparison. e**_,_** f) 3D and 2D charge density distribution. Isosurface unit: 0.0068 e Å^−3^, color legends for isosurface: cyan and yellow indicate charge depletion and charge accumulation, respectively. Fourier‐transformed *K* ^3^‐weighted g) Co *K*‐edge and h) Ru *K*‐edge extended x‐ray absorption fine structure (EXAFS) spectra of various samples. i) Co *K*‐edge and j) Ru *K*‐edge WT‐EXAFS spectra of R‐Co(OH)_2_, R‐RuO_2_@Co(OH)_2_, and R‐RuO_2_. k) Structural diagrams show the synergistic activation of H_2_O and NO_3_^−^ on RuO_2_/Co(OH)_2_.

The electronic structure of catalysts plays a critical role in determining the behavior of eNO_3_RR intermediates, including nitrogenous groups and *H, which can be probed by x‐ray photoelectron spectroscopy (XPS). In R‐Co(OH)_2_, the peaks at 782.1 and 786.9 eV correspond to Co^2+^ 2*p*_3/2_ and its satellite peak from Co(OH)_2_, respectively.^[^ ^21^ ^]^ In contrast, the Co^2+^ 2*p*_3/2_ in R‐RuO_2_/Co(OH)_2_ exhibits a shift to lower binding energy position by 0.6 eV, indicating increased electron density in Co compared to R‐Co(OH)_2_ (**Figure** **3**a). Similarly, the binding energy of Ru^4+^ in R‐RuO_2_/Co(OH)_2_ shifts negatively by 0.7 eV compared to R‐RuO_2_, suggesting higher electron density in Ru (Figure 3b). These results demonstrate that the electrochemical treatment of the RuO_2_ and Co_3_O_4_ precatalysts generates electron‐enriched Ru and Co centers in R‐RuO_2_/Co(OH)_2_, which serve as active sites for *H production (via water dissociation) and NO_3_^−^‐to‐NH_3_ conversion, respectively. Further insights into the electronic and coordination environments of Ru and Co in various reconstructed catalysts were obtained through x‐ray absorption spectroscopy (XAS).^[^ ^22^ ^]^ The normalized x‐ray absorbance near‐edge structure spectroscopy (XANES) shows the Co *K*‐edge position of R‐RuO_2_/Co(OH)_2_ between Co^0^ (Co foil) and Co*^α^* ^+^ (Co_3_O_4_, 2 < *α* < 3), confirming an intermediate oxidation state (valence state *δ*, 0 < *δ* < *α*) of Co. A slight shift to lower energy position compared to R‐Co(OH)_2_ indicates electron accumulation on Co, aligning with the XPS results (Figure 3c). These electron‐rich Co sites can serve as active centers to capture the nitrogenous groups during eNO_3_RR.^[^ ^23^ ^]^ Meanwhile, the Ru *K*‐edge XANES of R‐RuO_2_/Co(OH)_2_ exhibits a negatively shifted pre‐edge and reduced white line intensity relative to R‐RuO_2_, further supporting enhanced electron density at Ru (Figure 3d). Charge distribution analysis corroborates the electron‐rich nature of both Co and Ru in R‐RuO_2_/Co(OH)_2_ (Figure 3e,f). The increased electron density at Ru sites promotes H_2_O adsorption, accelerating *H production through water dissociation.

The coordinate environment of Co and Ru was further examined using extended x‐ray absorption fine structure (EXAFS) spectroscopy (Figures S13–S17, Supporting Information). For the *K* ^3^‐weighted *R*‐space Co *K*‐edge EXAFS, a lower intensity of Co−O scattering path in R‐RuO_2_/Co(OH)_2_ than that in R‐Co(OH)_2_ suggests a reduced coordination number on Co−O structures due to RuO_2_ incorporation (Figure 3g; Table S2, Supporting Information).^[^ ^24^ ^]^ The reduced coordination number is also observed on Ru−O shell of R‐RuO_2_/Co(OH)_2_ (Figure 3h; Table S3, Supporting Information), indicating that the electron‐rich electrochemical environment removes partial of surface oxygen atoms in RuO_2_ and Co(OH).^[^ ^24^ ^]^ Our previous study has shown the transformation of Co_3_O_4_ into Co_3_O_4_ with oxygen vacancies (O_vc_) and finally into Co(OH)_2_.^[^ ^4a^ ^]^ When treated at −0.3 V for 1 hour, high‐resolution O 1*s* XPS spectra from both R‐RuO_2_/Co(OH)_2_ and R‐Co(OH)_2_ reveal the loss of lattice oxygen from the oxides and the formation of hydroxide (Figure S18, Supporting Information). Moreover, a higher concentration of the adsorbed oxygen species (e.g., H_2_O) is detected on R‐RuO_2_/Co(OH)_2_ compared to the R‐Co(OH)_2_ counterpart. This enhanced ability to adsorb H_2_O in R‐RuO_2_/Co(OH)_2_ is attributed to the presence of RuO_2_, where the abundant O_vc_ create numerous sites for H_2_O dissociation, which is consistent with the following calculation results. Wavelet transform (WT) of the EXAFS profile further confirms the coordination structure (Figure 3i,j). R‐RuO_2_/Co(OH)_2_ exhibits resolved spectra of Co−O and Co−Co bonds at (*k*, *R*+*α*) space coordination of (1.6 Å, 5.6 Å^−1^) and (2.8 Å, 8.1 Å^−1^), similar to R‐Co(OH)_2_ but different from the Co−Co bond in Co foil (2.2 Å, 8.1 Å^−1^). Additionally, the resolved spectra of Ru−O and Ru−Ru bonds in R‐RuO_2_/Co(OH)_2_ were observed at (1.4 Å, 6.8 Å^−1^) and (3.3 Å, 12.2 Å^−1^), respectively, distinguished from those in Ru foil (2.3 Å, 10.3 Å^−1^). These results confirm that R‐RuO_2_/Co(OH)_2_ comprises RuO_2_ and Co(OH)_2_ phases rather than any metallic phases. Additionally, the fitting results of Ru *K*‐edge EXAFS profile indicate the slightly increased Ru−Ru/Co distance in R‐RuO_2_/Co(OH)_2_ compared to the R‐RuO_2_ counterpart. This change is attributed to the formation of a RuO_2_/Co(OH)_2_ interface, where the adjacent Ru and Co sites work cooperatively to influence the behavior of *H and N‐containing species (Table S3, Supporting Information). The Ru sites in RuO_2_ and Co sites in Co(OH)_2_ components of R‐RuO_2_/Co(OH)_2_, with their respective charge and chemical structure advantages at the interface, demonstrate a stronger ability to adsorb H_2_O and NO_3_^−^ species, offering significant potential to boost the eNO_3_RR kinetics for rapid NH_3_ formation through a site synergy effect (Figure 3k).

### 2.4 Tuning Active Hydrogen by Catalyst Design

**Figure 4:**
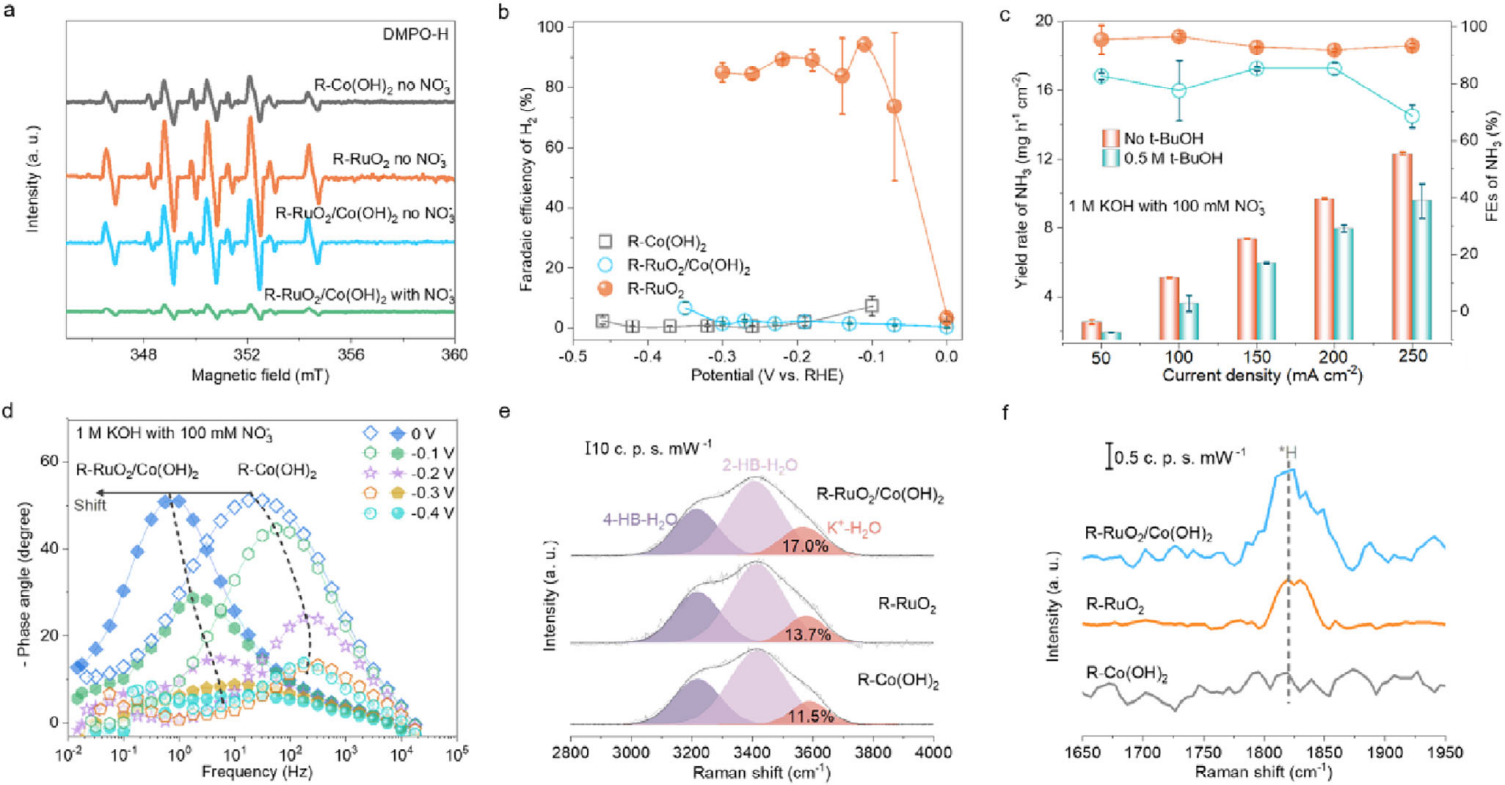
Tuning active hydrogen by catalyst design. a) ESR spectra of various catalysts in 1 m KOH with and/or without NO_3_^−^. b) FEs for H_2_ evolution over various catalysts during eNO_3_RR. c) Yield rates and FEs of NH_3_ over the R‐RuO_2_/Co(OH)_2_ catalyst in 1 m KOH containing 100 mm NO_3_^−^ with and without the addition of 0.5 m *t*‐BuOH. d) Potential‐dependent in situ Bode plots of the R‐Co(OH)_2_ and R‐RuO_2_/Co(OH)_2_ catalysts in 1 m KOH containing 100 mm NO_3_^−^. e,f) In situ Raman spectra of various catalysts in 1 m KOH containing 100 mm NO_3_^−^ at −0.3 V versus RHE. Error bars in insets (b,c) represent the standard deviation from three independent measurements.

Electron spin resonance (ESR) spectroscopy using a 5,5‐dimethyl‐1‐pyrroline‐N‐oxide (DMPO) as a radical trapping reagent was employed to directly validate the dynamic production and utilization of *H species (**Figure** **4**a). After the reaction at −0.3 V in 1 m KOH, nine peaks with an intensity ratio of approximately 1:1:2:1:2:1:2:1:1 were observed for all catalysts, attributed to the spin adduct of DMPO‐H.^[^ ^25^ ^]^ The DMPO‐H signal intensity follows the order of R‐RuO_2_>R‐RuO_2_/Co(OH)_2_>R‐Co(OH)_2_, confirming RuO_2_ as the active component for H_2_O‐to‐*H conversion. Upon adding NO_3_^−^ to the KOH electrolyte, the signals showed significantly reduced intensity, suggesting *H consumption in the hydrogenation of nitrogenous groups. Correspondingly, R‐RuO_2_ exhibited FEs of H_2_ above 70% at potentials below −0.1 V during eNO_3_RR, demonstrating preferential *H recombination over the hydrogenation of nitrogenous groups (Figure 4b). In contrast, R‐RuO_2_/Co(OH)_2_ maintained the FE for H_2_ below 1%, revealing efficient *H utilization for NH_3_ formation via spillover to Co(OH)_2_. This mechanism was further supported by significant decreases in NH_3_ yield rate and FE when tert‐butanol (*t*‐BuOH) was used as a trapping agent for *H (Figure 4c).^[^ ^26^ ^]^

The kinetics of *H formation on R‐Co(OH)_2_ and R‐RuO_2_/Co(OH)_2_ was investigated by using in situ electrochemical impedance spectroscopy (EIS) in 1 m KOH with (Figure 4d) or without (Figure S19, Supporting Information) 100 mm NO_3_^−^. The peak positions of Bode plots for R‐RuO_2_/Co(OH)_2_ shift to lower frequencies compared to R‐Co(OH)_2_ at the same applied potentials, indicating dominant *H production through water dissociation (Volmer step) on the R‐RuO_2_/Co(OH)_2_ catalyst, where the *H prefers to recombine through a Heyrovsky step on R‐Co(OH)_2_.^[^ ^27^ ^]^ The water dissociation behaviors during eNO_3_RR were further studied by in situ Raman spectroscopy under the conditions of 1 m KOH with 100 mm NO_3_^−^ and the applied potential of −0.3 V. Three distinct interfacial water configurations were resolved: 4‐coordinated hydrogen‐bonded water (4‐HB‐H_2_O, 3220 cm^−1^), 2‐coordinated hydrogen‐bonded water (2‐HB‐H_2_O, 3420 cm^−1^) and K^+^ ion hydrated water (K^+^‐H_2_O, 3600 cm^−1^).^[^ ^28^ ^]^ The significantly increased population of K^+^‐H_2_O on R‐RuO_2_/Co(OH)_2_ (17%) accounts for its notable *H production ability (Figure 4e; Table S4, Supporting Information), which results in the most pronounced *H peak at ∼1820 cm^−1^ on the R‐RuO_2_/Co(OH)_2_ catalyst.^[^ ^29^ ^]^ These *H species were effectively used for the hydrogenation of nitrogenous groups, driving the dramatic improvement in NH_3_ production.

### 2.5 Tuning Active Hydrogen by the Electrolyte Configuration

**Figure 5:**
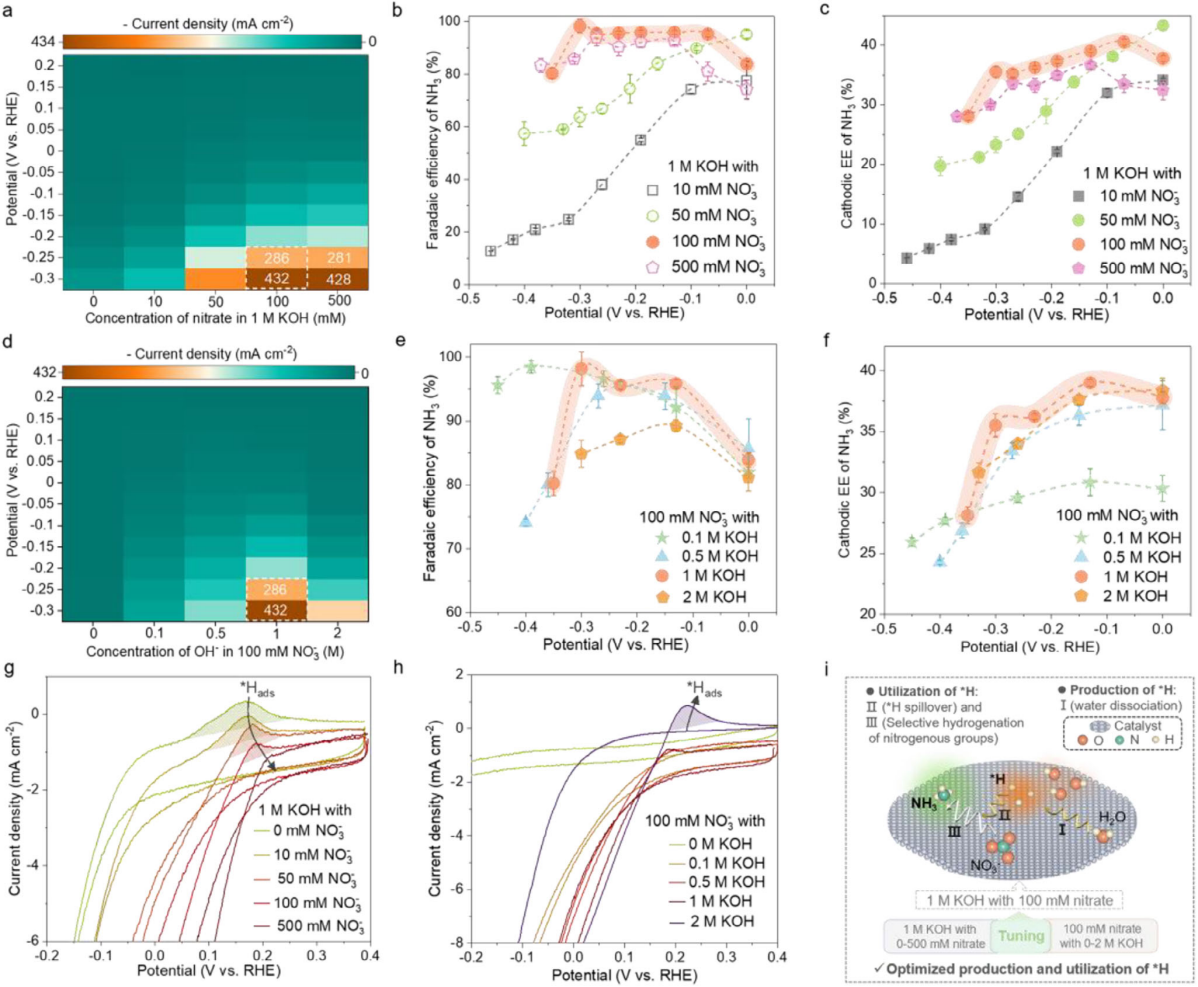
Tuning active hydrogen by controlling electrolyte compositions. a) Potential‐dependent current densities, b) FEs, and c) Half‐cell cathodic EEs of NH_3_ over R‐RuO_2_/Co(OH)_2_ in 1 m KOH with various concentrations of NO_3_^−^. d) Potential‐dependent current densities, e) FEs, and f) Half‐cell cathodic EEs of NH_3_ over R‐RuO_2_/Co(OH)_2_ in 100 mm NO_3_^−^ with various concentrations of KOH. CV curves in g) 1 m KOH with various concentrations of NO_3_^−^, and h) 100 mm NO_3_^−^ with various concentrations of KOH. i) Illustration of the production and utilization of active hydrogen through water dissociation and eNO_3_RR with a cooperative effect. Error bars in insets (b,c,e,f) represent the standard deviation from three independent measurements.

The eNO_3_RR performance was optimized by adjusting the electrolyte composition, e.g. the concentration of OH^−^ and NO_3_^−^, to balance the active hydrogen production and utilization over the R‐RuO_2_/Co(OH)_2_ catalyst for the rapid NH_3_ production. Increasing nitrate concentrations from 0 to 500 mm improves current densities, peaking at 100 mm (**Figure** **5**a). The FEs and energy efficiencies (EEs) of NH_3_ decrease with more negative potentials below 50 mm NO_3_^−^, due to excess active hydrogen not being used for hydrogenation (Figure 5b; Figures S20 and S21, Supporting Information).^[^ ^30^ ^]^ At 100 and 500 mm of NO_3_^−^, the FEs and EEs of NH_3_ maintain above 80% and 25%, respectively, with the highest FE and EE of 98.1 ± 2.6% and 40.5 ± 0.5% at 100 mm NO_3_^−^ (Figure 5c). This concentration is optimal for balancing active hydrogen production and utilization by the hydrogenation of N‐containing groups while inhibiting *H recombination into H_2_. Nitrate concentration has negligible effects on reaction kinetics, as indicated by similar Tafel slopes (Figure S22, Supporting Information), but improves the NH_3_ selectivity through enriched nitrogenous reactants on the catalysts’ surface.

In addition to the reactant concentration, electrolytes’ pH is also an important factor influencing the eNO_3_RR kinetics and selectivity.^[^ ^31^ ^]^ With 100 mm NO_3_^−^ while varying KOH concentration from 0 to 2 m (corresponding to pH values of 5.6 to 14.5), the current densities of R‐RuO_2_/Co(OH)_2_ increase with KOH concentration, plateauing at 1 m (Figure 5d; Figure S23, Supporting Information). If the electrolyte solely contains nitrate, the NH_3_ yield is only 1.07 ± 0.09 mg h^−1^ cm^−2^ at −0.3 V, despite a FE of 73.2 ± 6.2%, suggesting that the NO_3_^−^‐to‐NH_3_ conversion in pure NO_3_^−^ has very slow kinetics due to limited mass transfer (Figure S24, Supporting Information).^[^ ^31a^ ^]^ Above 0.1 m KOH in the electrolyte, the NH_3_ FEs are over 80% with significantly boosted yield rates. Particularly at 1 m KOH, the largest yield rate and FE (>95%) of NH_3_ are attributed to the optimal active hydrogen production and selective utilization. However, at 2 m KOH, the generated *H species overwhelmingly cover catalysts’ surface to recombine as H_2_, reducing NH_3_ FEs below 90%, consistent with the half‐cell EE trend (Figure 5e,f). Electrolytes with only nitrate produce ammonia slower, but benefit from KOH additives to accelerate kinetics, evidenced by the decreased Tafel slopes and reduced *R*_1_ and *R*_2_ values in 1 m KOH with 100 mm NO_3_^−^, where *R*_1_ is the resistance of electron transfer from the reaction interface to reactants and intermediates, and *R*_2_ is the resistance of electron transfer from the electrode to surface sites of the catalyst^[^ ^27^ ^]^ (Figures S25 and S26, Supporting Information). As a result, the R‐RuO_2_/Co(OH)_2_ catalyst achieves the highest NH_3_ activity and selectivity in 1 m KOH with 100 mm NO_3_^−^. The enhanced kinetics and selectivity are closely related to the optimized *H behaviors. CV curves show a distinct oxidation peak between 0.05 and 0.2 V versus RHE, indicating *H desorption, essential for hydrogenation reactions (Figure 5g,h).^[^ ^32^ ^]^ Increasing NO_3_^−^ concentrations weakens *H desorption peaks, indicating efficient *H utilization. These results demonstrate that eNO_3_RR activity and selectivity can be tuned by varying the NO_3_^−^ and OH^−^ concentrations, achieving optimal active hydrogen production and utilization in 1 m KOH containing 100 mm NO_3_^−^ for the R‐RuO_2_/Co(OH)_2_ catalyst (Figure 5i).

### 2.6 eNO_3_RR Performance

**Figure 6:**
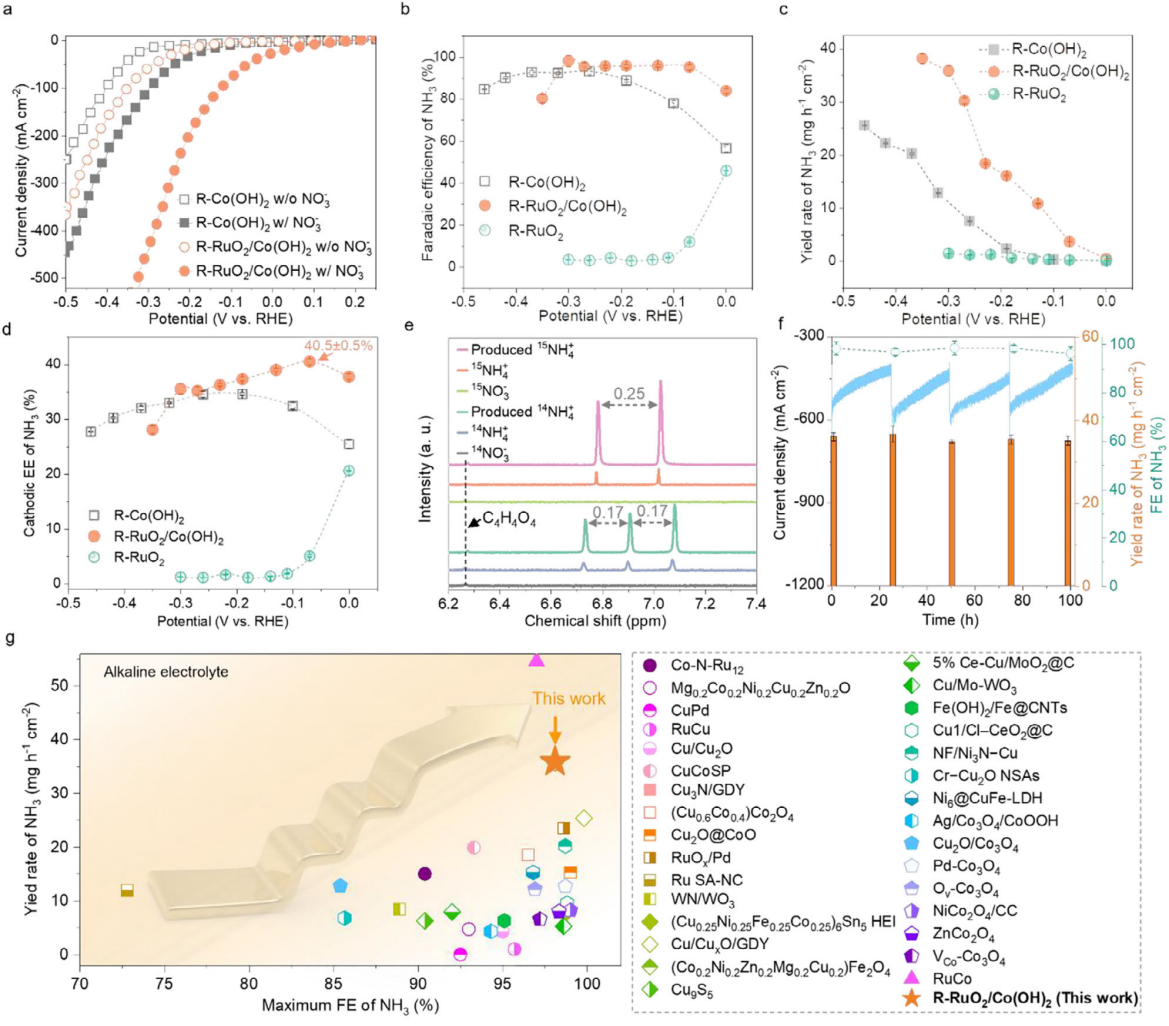
eNO_3_RR performance. a) Linear sweep voltammetry (LSV) curves of the R‐Co(OH)_2_ and R‐RuO_2_/Co(OH)_2_ catalysts in 1 m KOH with (w/) and without (w/o) 100 mm NO_3_^−^, scan rate: 5 mV s^−1^. b) Faradaic efficiencies (FEs), c) yield rate, and d) half‐cell cathodic energy efficiency (EE) of NH_3_ over various catalysts. e) ^1^H nuclear magnetic resonance (NMR) spectra of various electrolytes by ^15^N and ^14^N isotope‐labeling experiments. f) Stability test at −0.3 V for 100 hours and the corresponding yield rates and FEs of NH_3_ over the R‐RuO_2_/Co(OH)_2_ catalyst. g) Comparison of ammonia synthesis performance from eNO_3_RR over the R‐RuO_2_/Co(OH)_2_ with other reported catalysts in alkaline electrolytes. Error bars in insets (b,c,d,f) represent the standard deviation from three independent measurements.

The eNO_3_RR performance was assessed in an H‐type cell under ambient conditions using 1 m KOH with and without 100 mm NO_3_^−^ as electrolytes. The electrolytes were separated by a Nafion 117 membrane to prevent ammonia consumption at the anode. Linear sweep voltammetry (LSV) curves were recorded to evaluate the eNO_3_RR activity. All catalysts exhibit an enhanced current density in 1 m KOH with 100 mm NO_3_^−^ compared to the case without NO_3_^−^, suggesting nitrate's significant role in contributing the current density increase for eNO_3_RR (**Figure** **6**a; Figure S27, Supporting Information).^[^ ^33^ ^]^ Among these catalysts, R‐RuO_2_/Co(OH)_2_ shows the largest current density increase, demonstrating its comparable potential for NH_3_ production. Chronoamperometry tests conducted at various potentials aim to evaluate both activity and selectivity, with the resulting ammonia quantified by ultraviolet‐visible (UV–vis) absorbance spectra (Figures S28–S30, Supporting Information). Compared to R‐RuO_2_ and R‐Co(OH)_2_, R‐RuO_2_/Co(OH)_2_ shows significantly improved FEs for NH_3_ below −0.3 V versus RHE, suggesting a synergistic effect of RuO_2_ and Co(OH)_2_ on NH_3_ formation (Figure 6b; Figure S31, Supporting Information). This effect is attributed to the abundant *H supply from RuO_2_ to Co(OH)_2_ through a spillover effect,^[^ ^34^ ^]^ which facilitates the hydrogenation of nitrogenous groups on Co(OH)_2_. The FEs of NO_2_^−^, a primary by‐product, were also examined (Figures S32–S34, Supporting Information).^[^ ^35^ ^]^ The lower FEs of NO_2_^−^ and higher FEs of NH_3_ across all potentials for R‐RuO_2_/Co(OH)_2_ indicate effective conversion of NO_2_^−^ into NH_3_. Therefore, it shows the best selectivity, with FEs of NH_3_ exceeding 95% over a wide potential window between −0.05 and −0.3 V. Importantly, it achieves the highest ammonia FEs of 98.1 ± 2.6% at −0.3 V with a yield rate of 35.9 ± 0.9 mg h^−1^ cm^−2^ (Figure 6b,c). These results indicate that the kinetics of nitrate‐to‐ammonia conversion is significantly accelerated by combining RuO_2_ and Co(OH)_2_, allowing for more efficient utilization of these active components. Consequently, the R‐RuO_2_/Co(OH)_2_ catalyst presents higher energy utilization compared to other counterparts, with a peak EE of 40.2 ± 0.5% (Figure 6d).^[^ ^35^ ^]^

^14^N‐ and ^15^N‐isotopic labeling experiments were performed, and the ^1^H nuclear magnetic resonance (NMR) spectra of the electrolytes before and after catalysis at −0.3 V were measured to confirm ammonia production via eNO_3_RR (Figure 6e).^[^ ^36^ ^]^ The ^1^H NMR spectra exhibit characteristic splitting patterns matching those of standard ^14^NH_4_^+^ and ^15^NH_4_^+^ for the respective isotopic reactants. In contrast, no ^1^H signals are detected in the absence of ammonia. Combined with the overlapped absorbance spectra of 1 m KOH before and after the treatment at −0.3 V and the catalysts before and after soaking in 1 m KOH with 100 mm NO_3_^−^, the possibility of environmental and catalysis setup contamination is ruled out, demonstrating that the detected ammonia originates from eNO_3_RR (Figure S35, Supporting Information).

The eNO_3_RR stability of R‐RuO_2_/Co(OH)_2_ was investigated at −0.3 V for 100 hours. After continuous operation for 25 hours (Figure 6f), the electrolyte was refreshed to determine the NH_3_ FEs. The gradually decreased current density with extended operation time is attributed to the reduced NO_3_^−^ concentration rather than the deactivation of R‐RuO_2_/Co(OH)_2_. Upon replacing with fresh electrolyte, the current densities return to initial levels. After 100 hours of catalysis, R‐RuO_2_/Co(OH)_2_ maintains a high NH_3_ FE of 96.3 ± 3.0%, indicating its reliable stability for nitrate waste removal and NH_3_ production. The produced NH_3_ was recovered using an argon stripping‐acid trapping‐evaporation method.^[^ ^37^ ^]^ After implementing under argon stripping and acid trapping treatments (Figure S36, Supporting Information), only 11.2 ± 1.9 ppm of ammonia remained in the reacted electrolyte (Figure S37a, Supporting Information). UV–vis measurements indicate that 99 ± 1% of NH_3_ was removed from the reacted electrolyte, with 97.7 ± 0.3% of this NH_3_ effectively trapped by the acid solution. Through an evaporation process, the trapped NH_3_ was converted into NH_4_Cl powder, yielding 6.072 g of collected NH_4_Cl, which corresponds to a collection rate of 95.2% (Figure S37b,c, Supporting Information). The NH_4_Cl phase of the collected product was further confirmed by XRD patterns (Figure S37d, Supporting Information). These results demonstrate that the argon stripping‐acid trapping‐evaporation strategy is effective for the extraction and collection of ammonia, showcasing its potential application in ammonia recovery processes. The collected NH_4_Cl can be used as a raw material for fertilizer, refrigeration, textile, pharmaceutical, and energy storage and transportations. These findings demonstrate the notable activity, selectivity, and stability of R‐RuO_2_/Co(OH)_2_ for eNO_3_RR to NH_3_, outperforming mostly reported catalysts (Figure 6g; Table S5, Supporting Information).

Additional electrochemical measurements were conducted to understand the underlying mechanisms of performance improvement for R‐RuO_2_/Co(OH)_2_. R‐Co(OH)_2_ shows an increased *C*_dl_ value in 1 m KOH containing 100 mm NO_3_^−^ compared to the case without NO_3_^−^, while R‐RuO_2_ exhibits an opposite trend (Figures S38 and S39, Supporting Information), suggest that Co(OH)_2_ serves as the active component for nitrate binding, while RuO_2_ acts as the active phase for water/hydroxide ion adsorption. Through a cooperative effect between Co(OH)_2_ and RuO_2_, R‐RuO_2_/Co(OH)_2_ achieves the best NH_3_ synthesis performance. This cooperative effect is also facilitated by faster reaction kinetics, evidenced by a lower Tafel slope of 211 mV dec^−1^ for the R‐RuO_2_/Co(OH)_2_ (Figure S40, Supporting Information). The kinetics were further probed by in situ EIS (Figure S41, Supporting Information). An equivalent circuit model is established to illustrate the electrochemical behaviors at the catalyst/electrolyte interface, where *R*_s_ represents the solution resistance, *R*_1_ is the resistance of electron transfer from the reaction interface to reactants and intermediates, and *R*_2_ is the resistance of electron transfer from the electrode to surface sites of the catalyst.^[^ ^27^ ^]^ Compared to R‐Co(OH)_2_, the *R*_1_ values of R‐RuO_2_/Co(OH)_2_ remain lower across the tested potentials, indicating more ready interaction of intermediates and active sites at the catalyst/electrolyte interface, accounting for the greatly boosted kinetics and larger NH_3_ yields (Figure S42, Supporting Information). Overall, a synergistic effect of RuO_2_ and Co(OH)_2_ in R‐RuO_2_/Co(OH)_2_ ensures a significantly accelerated rate of NH_3_ formation during eNO_3_RR.

### 2.7 Mechanistic Insights

**Figure 7:**
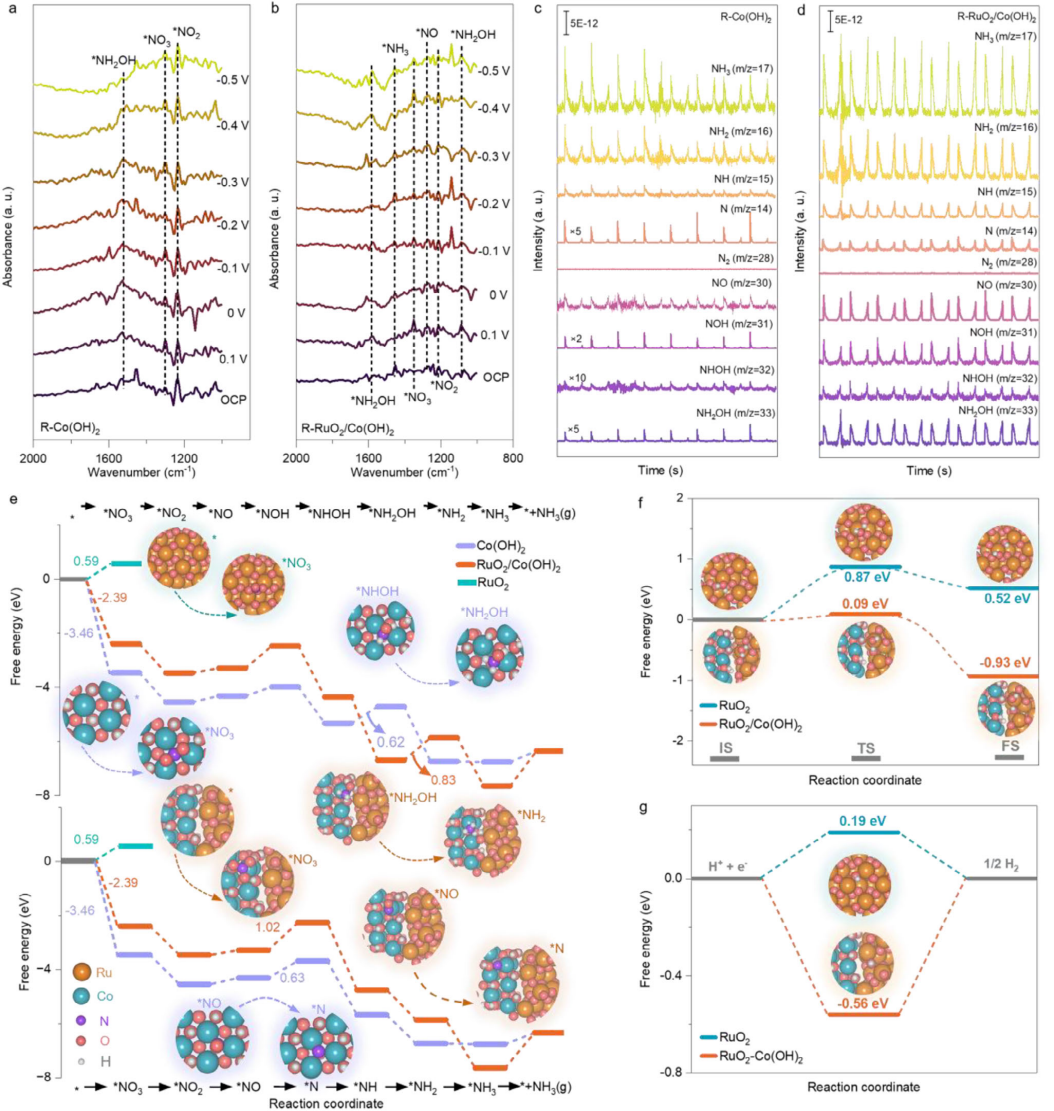
Mechanistic analysis. Potential‐dependent electrochemical in situ Fourier transform infrared spectroscopy (FTIR) spectra in 1 m KOH containing 100 mm NO_3_^−^ over a) R‐Co(OH)_2_, and b) R‐RuO_2_/Co(OH)_2_. Online differential electrochemical mass spectrometry (DEMS) of c) R‐Co(OH)_2_, and d) R‐RuO_2_/Co(OH)_2_ in 1 m KOH containing 100 mm NO_3_^−^. e) Energy profiles of various catalysts during eNO_3_RR with different reaction pathways. f) Calculated kinetic barriers of water dissociation (IS: initial state; TS: transition state; FS: final state). g) Free energy diagrams of *H intermediates adsorbed on various nanostructures.

The eNO_3_RR intermediates were preliminarily tracked using in situ Fourier transform infrared spectroscopy (FTIR) as a function of potential. The FTIR spectrum acquired at open circuit potential (OCP) served as the reference, with the electrolyte composed of 1 m KOH containing 100 mm NO_3_^−^. As shown in **Figure** **7**a, characteristic peaks at approximately 1236, 1300, and 1520 cm^−1^ are ascribed to the absorbance bands of *NO_2_, *NO_3_, and *NH_2_OH species, respectively. This finding suggests that the nitrogenous conversion processes on R‐Co(OH)_2_ involve the generation and transformation of these intermediates.^[^ ^38^ ^]^ After the incorporation of RuO_2_ onto Co(OH)_2_, three additional peaks at 1085, 1280, and 1456 cm^−1^ were detected for the R‐RuO_2_/Co(OH)_2_ catalyst electrode (Figure 7b). These peaks are assigned to the stretching vibration of N–O in*NH_2_OH, the *NO vibration band, and the N–H bending band in *NH_3_, respectively.^[^ ^38^, ^39^ ^]^ These results indicate that the combination of RuO_2_ with Co(OH)_2_ enriches the conversion pathways of reaction intermediates with a component synergistic effect. To further clarify the reaction pathway of reactant and intermediate conversion, online differential electrochemical mass spectrometry (DEMS) measurements were performed.^[^ ^40^ ^]^ During periodic changes of cyclic voltammetry (CV) cycles between −0.6 and 0.4 V versus RHE, mass‐to‐charge (m/z) signals of various intermediates including 33(NH_2_OH), 32 (NHOH), 31(NOH), 30 (NO), 14 (N), 15 (NH), 16 (NH_2_), and 17 (NH_3_) were collected during the eight continuous cycles (Figure 7c,d). These signals are detectable for both R‐Co(OH)_2_ and R‐RuO_2_/Co(OH)_2_. However, the latter consistently exhibits significantly higher signal intensities for various intermediates. These findings indicate that the RuO_2_ incorporation does not alter the reaction pathway but facilitates the kinetics for fast ammonia formation. According to in situ FTIR and online DEMS results, eNO_3_RR to NH_3_ over R‐RuO_2_/Co(OH)_2_ followed two possible pathways: (1) *NO→*NOH→*NHOH→*NH_2_OH→*NH_2_→*NH_3_; (2) *NO→*N→*NH→*NH_2_→*NH_3_. The significantly higher signal intensities of NH_2_OH, NHOH, and NOH species in pathway 1 compared to those of N and NH species observed in pathway 2 indicate that the former is the dominant reaction path for the eNO_3_RR over R‐RuO_2_/Co(OH)_2_. Density functional theory (DFT) calculations were conducted to understand the positive role of active hydrogen in enhancing NH_3_ yield, where structures interacted with various intermediates were considered (Figures S43 and S44, Supporting Information). Initially, NO_3_^−^ should be easily adsorbed on catalysts to initiate eNO_3_RR.^[^ ^41^ ^]^ A NO_3_^−^ adsorption energy of 0.59 eV for RuO_2_ indicates its limited ability to proceed nitrate reduction. In contrast, RuO_2_/Co(OH)_2_ and Co(OH)_2_ with adsorption energies of −2.39 and −3.46 eV demonstrate much better NO_3_^−^ capture ability for further reactions (Figure 7e). Regarding this, free energy calculations were performed on Co(OH)_2_ and RuO_2_/Co(OH)_2_. The calculations show the largest uphill free energies of 0.62 and 0.63 eV for Pathways (1) and (2) on Co(OH)_2_, which correspond to the hydrogenation of *NHOH and the deoxygenation of *NO intermediate, respectively, making these processes the rate‐determining steps (RDSs). For R‐RuO_2_/Co(OH)_2_, the conversion of *NH_2_OH to *NH_2_, and *NO to *N are the RDSs, with free energies of 0.83 and 1.02 eV, respectively. Decreases in Gibbs free energies for the conversion of *N to *NH species are observed in both RuO_2_/Co(OH)_2_ and Co(OH)_2_. However, the *N−*N coupling on these structures requires very high energy barriers of 3.87 and 0.61 eV, respectively (Figures S45, Supporting Information). These theoretical results demonstrate that R‐RuO_2_/Co(OH)_2_ favors the selective ammonia production rather than the NO_3_^−^‐to‐N_2_ conversion, consistent with the experimental evidence from online DEMS. RuO_2_ incorporation makes NO_3_^−^‐to‐NH_3_ conversion on Co(OH)_2_ slightly more difficult thermodynamically with larger energy barriers for the RDSs, but it obviously improves reaction kinetics while maintaining high selectivity. Evidence is that RuO_2_/Co(OH)_2_ (Figure 7f) shows a much lower energy barrier (0.09 eV) for water dissociation than that of the pristine RuO_2_ (0.87 eV).^[^ ^42^ ^]^ Co(OH)_2_, covered with OH motifs, is inaccessible to water molecules. These findings indicate that combining RuO_2_ with Co(OH)_2_ significantly accelerates water dissociation, providing abundant active hydrogen for the selective hydrogenation of nitrogenous groups. The active hydrogen species produced on either RuO_2_ or Co(OH)_2_ alone tend to desorb into electrolytes with positive adsorption energies (Figure 7g; Figure S46, Supporting Information). However, the adsorption energy of *H on RuO_2_/Co(OH)_2_ is −0.56 V, suggesting a stronger *H adsorption but a lower *H recombination trend. These *H species accumulate, transfer, and finally hydrogenate nitrogenous groups on adjacent Co(OH)_2_, resulting in high ammonia yields and FEs over the RuO_2_/Co(OH)_2_ catalyst.

### 2.8 Extended Applications

**Figure 8:**
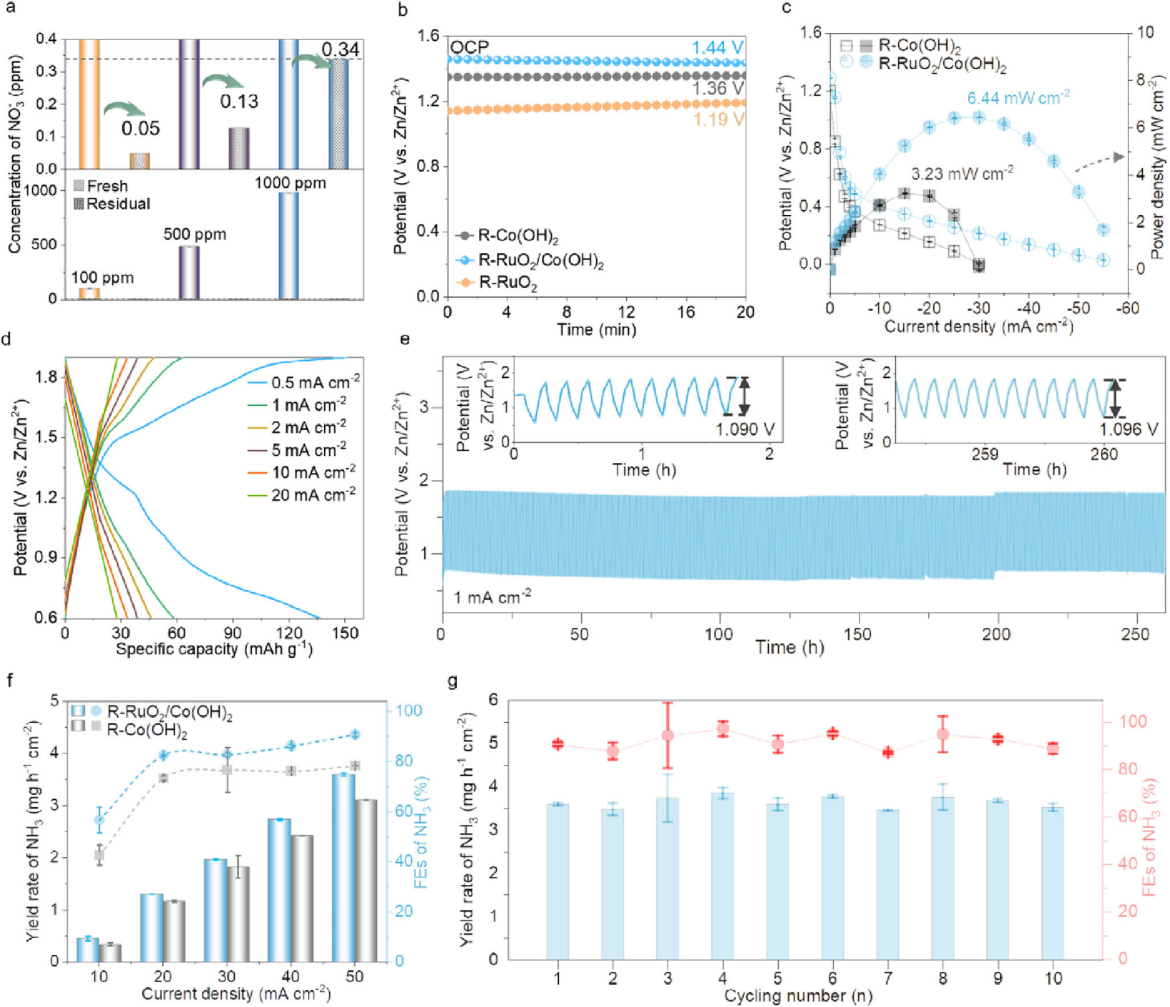
Applications for nitrate waste removal and rechargeable zinc‐nitrate batteries. a) Concentrations of residual NO_3_^−^ after the operation at −0.3 V for 20 hours over the R‐RuO_2_/Co(OH)_2_ catalyst in 1 m KOH containing 100, 500, and 1000 ppm of NO_3_^−^. b) Open‐circuit potential (OCP) of Zn‐NO_3_^−^ batteries assembled by R‐Co(OH)_2_, R‐RuO_2_/Co(OH)_2_, and R‐RuO_2_ cathodes. c) Discharge curves from OCP to 0 V and their corresponding power densities. d) Charge and discharge curves at various current densities for the R‐RuO_2_/Co(OH)_2_ cathode. e) Stability test at a constant current density of 1 mA cm^−2^ for 260 hours. f) Yield rate and FEs of NH_3_ at various current densities in the Zn‐NO_3_^−^ batteries configured with R‐Co(OH)_2_ and R‐RuO_2_/Co(OH)_2_. g) Stability test for NH_3_ production at 50 mA cm^−2^ in a Zn‐NO_3_^−^ battery configured with R‐RuO_2_/Co(OH)_2_. Error bars in insets (c,f,g) represent the standard deviation from three independent measurements.

Considering the notable activity of catalyzing NO_3_^−^ to NH_3_, R‐RuO_2_/Co(OH)_2_ was used as a catalyst to remove nitrate waste (initial concentrations of 100, 500, and 1000 ppm) (Figures S47 and S48, Supporting Information). The residual contents are 0.05, 0.13, and 0.34 ppm (**Figure** **8**a) after working for 20 hours at −0.3 V, significantly below the World Health Organization (WHO) nitrate standard (50 ppm NO_3_^−^) in drinking water,^[^ ^43^ ^]^ indicating R‐RuO_2_/Co(OH)_2_′s comparable ability to purify nitrate from wastewater. Given the notable eNO_3_RR stability of R‐RuO_2_/Co(OH)_2_, we applied it as a cathode material in a Zn‐NO_3_^−^ battery.^[^ ^44^ ^]^ The cathodic electrolyte is 1 m KOH containing 0.1 m NO_3_^−^, while the anodic electrolyte is 6 m KOH with 0.2 m Zn(Ac)_2_, separated by a bipolar membrane. The assembled battery with R‐RuO_2_/Co(OH)_2_ achieves the highest open circuit potential (OCP) of 1.44 V (vs Zn/Zn^2+^), reaching 89.4% of the theoretical potential (Figure 8b). The output current density increases with the negatively reduced potential in the discharging polarization curves, achieving a maximum power density of 6.44 mW cm^−2^ for the R‐RuO_2_/Co(OH)_2_ (Figure 8c), superior to that of R‐Co(OH)_2_ (3.23 mW cm^−2^). The Zn‐NO_3_^−^ battery with R‐RuO_2_/Co(OH)_2_ shows better rate performance across current densities of 0.5 to 50 mA cm^−2^ (Figure S49, Supporting Information), with specific discharging capacities of 150.7, 62.8, 47.5, 38.7, 33.3, and 28 mAh g^−1^ at current densities of 0.5, 1, 2, 5, 10, and 20 mA cm^−2^ (Figure 8d). Impressively, the battery operates stably for 260 hours at 1 mA cm^−2^, with the potential gap increasing by only 6 mV, demonstrating its reliable durability (Figure 8e). The NH_3_ synthesis performance at the cathode was also investigated (Figure S50, Supporting Information). For all current densities, the R‐RuO_2_/Co(OH)_2_ cathode always exhibits the largest yield rates and FEs for NH_3_ (Figure 8f). Specifically, it achieves an impressive ammonia FE of 90.7 ± 0.7% and a yield rate of 3.60 ± 0.03 mg h^−1^ cm^−2^ at 50 mA cm^−2^. After ten cycles, the retention rates of FE and yield rate are 98% (88.9 ± 2.1%) and 97.8% (3.52 ± 0.09 mg h^−1^ cm^−2^), respectively, indicating a good stability (Figure 8g). The Zn‐NO_3_^−^ battery with an R‐RuO_2_ cathode displays a slightly higher power density (8.17 mW cm^−2^) but significantly decreased FEs and yield rates of NH_3_ (Figure S51, Supporting Information). These results, along with the performance comparison to other recently reported catalysts (Table S6, Supporting Information), demonstrate that R‐RuO_2_/Co(OH)_2_ is one promising material for purifying nitrate‐containing wastewater and for use in the rechargeable Zn‐NO_3_^−^ battery.

## 3 Conclusion

In summary, we have strategically engineered RuO_2_/Co_3_O_4_ precatalysts for eNO_3_RR to ammonia. The potential‐ and time‐dependent reconstruction pattern from Co_3_O_4_ to Co(OH)_2_ were elucidated using combined in situ and ex situ characterizations, where RuO_2_ maintained its phase stability. Experimental and theoretical results reveal that the in situ reconstructed RuO_2_/Co(OH)_2_ catalyst consists of electron‐enriched Ru in RuO_2_ and Co in the Co(OH)_2_. RuO_2_ with good water dissociation but weak *H adsorption ability functions as the active component for the production and supply of *H species, while Co(OH)_2_ accepts *H to promote hydrogenation of nitrogenous groups and selective ammonia formation. This synergistic effect is enabled by effective production and utilization of active hydrogen in a combined manner of precatalyst design and the optimization of electrolyte's composition (OH^−^ and NO_3_^−^ concentration). Consequently, in 1 m KOH containing 100 mm NO_3_^−^, the optimized R‐RuO_2_/Co(OH)_2_ catalyst achieves an impressive ammonia yield rate of 35.9 ± 0.9 mg h^−1^ cm^−2^ with a FE of 98.1 ± 2.6% at −0.3 V versus RHE. Even after 100 hours of catalysis, the FE remains at 96.3 ± 3.0%, confirming its comparable stability. Additionally, R‐RuO_2_/Co(OH)_2_ shows notable advantages for nitrate‐containing wastewater remediation (reduces the nitrate less than 1 ppm) and rechargeable Zn‐NO_3_^−^ batteries (operate for 260 hours at 1 mA cm^−2^ with a potential window increase of only 6 mV).

## 4 Experimental Section

### Synthesis of RuO_2_ Precatalyst

Typically, 208 mg of RuCl_3_.*x*H_2_O (Sigma‐Aldrich, ≥ 99.9%) and 5 g of LiNO_3_ were added into a mortar and ground to achieve a uniform mixture. The temperature of a muffle furnace was elevated to 380 °C with a heating rate of 5 °C min^−1^. Once the target temperature was reached, the furnace gate was quickly opened, and the boat containing the mixture was placed into the furnace chamber for a 15‐minute reaction. Afterward, the boat was removed from the furnace and allowed to cool naturally in the air. The products were then soaked in deionized water to remove nitrate impurities, collected by centrifugation, and dried in a vacuum oven at 60 °C for 12 hours.

### Synthesis of Co_3_O_4_ Precatalyst

The preparation route of Co_3_O_4_ octahedrons is identical to the synthesis of RuO_2_, except that 237.93 mg of CoCl_2_.6H_2_O (Aladdin, ≥ 98%) was used instead of 208 mg of RuCl_3_.*x*H_2_O.

### Synthesis of RuO_2_/Co_3_O_4_ Precatalyst

First, 237.93 mg of CoCl_2_.6H_2_O (Aladdin, ≥ 98%), 5 g of LiNO_3_, and a specific amount (5.2, 10.4, or 20.8 mg) of RuCl_3_.xH_2_O (Sigma‐Aldrich, ≥ 99.9%) was mixed uniformly and ground together. Following the same reaction process at 380 °C, the resulting samples were analyzed by inductively coupled plasma optical emission spectroscopy (ICP‐OES). It is determined that the molar ratios of Ru versus Co atom in samples prepared with 5.2, 10.4, and 20.8 mg of RuCl_3_.*x*H_2_O were 0.3%, 1.1%, and 2.8%, respectively. Therefore, the samples are denoted as RuO_2_/Co_3_O_4_‐0.3, RuO_2_/Co_3_O_4_‐1.1, and RuO_2_/Co_3_O_4_‐2.8.

### Electrochemical Conditions to Investigate the Reconstruction Mechanism

To investigate the potential‐ and time‐dependent reconstruction mechanism, Co_3_O_4_, RuO_2_, and RuO_2_/Co_3_O_4_ precatalyst electrodes were operated at potentials between 0 and −0.3 V versus RHE in 1 m KOH containing 100 mm NO_3_^−^ for 1 hour, and at −0.3 V for different time periods. When the potential was −0.3 V, the derived catalysts from Co_3_O_4_, RuO_2_, and RuO_2_/Co_3_O_4_ precatalysts were denoted as R‐Co(OH)_2_, R‐RuO_2_, and R‐RuO_2_/Co(OH)_2_ for further structural and compositional characterization, respectively.

## Conflict of Interest

The authors declare no conflict of interest.

## Author Contributions

A.Q.Z. conceptualized the idea for the study, performed the data curation, formal analysis, and investigation, designed the methodology for the study, and wrote the original draft. H.L. designed the methodology for the study, performed the data curation, developed the software, performed the simulation, and wrote, reviewed, and edited the manuscript. L.L.Q. performed the investigation, designed the methodology for the study, performed the formal analysis, and wrote, reviewed, and edited the manuscript. B.L. performed the supervision and funding acquisition, and wrote, reviewed, and edited the manuscript. K.L.L., C.H.L., K.L., and J.P.L. performed the investigation, designed the methodology, and performed the validation. Y.Z., D.W.L., and G.Q.G. performed the formal analysis, designed the methodology for the study, performed the validation, and wrote, reviewed, and edited the manuscript. G.H. performed the supervision, funding acquisition, and validation, and wrote, reviewed, and edited the manuscript. W.J.Z. conceptualized the idea for the study, performed the supervision, funding acquisition, and project administration, and wrote, reviewed, and edited the manuscript.

## Supporting information



Supporting Information

## Data Availability

The data that support the findings of this study are available from the corresponding author upon reasonable request.

## References

[1] a) D. R. MacFarlane , P. V. Cherepanov , J. Choi , B. H. R. Suryanto , R. Y. Hodgetts , J. M. Bakker , F. M. Ferrero Vallana , A. N. Simonov , Joule 2020, 4, 1186; 10.1038/s41467-020-19130-z PMC7641139 33144566

[2] a) W. Chen , X. Yang , Z. Chen , Z. Ou , J. Hu , Y. Xu , Y. Li , X. Ren , S. Ye , J. Qiu , J. Liu , Q. Zhang , Adv. Funct. Mater. 2023, 33, 2300512;

[3] a) L. Bai , F. Franco , J. Timoshenko , C. Rettenmaier , F. Scholten , H. S. Jeon , A. Yoon , M. Ruscher , A. Herzog , F. T. Haase , S. Kuhl , S. W. Chee , A. Bergmann , R. C. Beatriz , J. Am. Chem. Soc. 2024, 146, 9665; 38557016 10.1021/jacs.3c13288 PMC11009949

[4] a) A. Zhu , H. Liu , S. Bu , K. Liu , C. Luan , D. Lin , G. Gan , Y. Zhou , T. Zhang , K. Liu , G. Hong , H. Li , W. Zhang , ACS Nano 2024, 18, 22344; 39106490 10.1021/acsnano.4c06637

[5] a) X. Fu , Chin. J. Catal. 2023, 53, 8;

[6] a) E. Murphy , B. Sun , M. Ruscher , Y. Liu , W. Zang , S. Guo , Y. H. Chen , U. Hejral , Y. Huang , A. Ly , I. V. Zenyuk , X. Pan , J. Timoshenko , B. R. Cuenya , E. D. Spoerke , P. Atanassov , Adv. Mater. 2024, 36, 2401133; 10.1002/adma.202401133 38619914

[7] a) D. Liu , L. Qiao , S. Peng , H. Bai , C. Liu , W. F. Ip , K. H. Lo , H. Liu , K. W. Ng , S. Wang , X. Yang , H. Pan , Adv. Funct. Mater. 2023, 33, 2303480;

[8] J. Zhang , T. Quast , B. Eid , Y.‐T. Chen , R. Zerdoumi , S. Dieckhöfer , J. R. C. Junqueira , S. Seisel , W. Schuhmann , Nat. Commun. 2024, 15, 8583. 39362855 10.1038/s41467-024-52780-x PMC11450097

[9] K. Fan , W. Xie , J. Li , Y. Sun , P. Xu , Y. Tang , Z. Li , M. Shao , Nat. Commun. 2022, 13, 7958. 36575160 10.1038/s41467-022-35664-w PMC9794814

[10] Y. Zhou , L. Zhang , Z. Zhu , M. Wang , N. Li , T. Qian , C. Yan , J. Lu , Angew. Chem., Int. Ed. 2024, 63, 202319029. 10.1002/anie.202319029 38449084

[11] X. Ouyang , W. Qiao , Y. Yang , B. Xi , Y. Yu , Y. Wu , J. Fang , P. Li , S. Xiong , Angew. Chem., Int. Ed. 2025, 64, 202422585. 10.1002/anie.202422585 39776195

[12] a) X. Liu , J. Meng , J. Zhu , M. Huang , B. Wen , R. Guo , L. Mai , Adv. Mater. 2021, 33, 2007344; 10.1002/adma.202007344 34050565

[13] W. Jang , D. Oh , J. Lee , J. Kim , J. E. Matthews , H. Kim , S.‐W. Lee , S. Lee , Y. Xu , J. M. Yu , S. W. Hwang , T. F. Jaramillo , J.‐W. Jang , S. Cho , J. Am. Chem. Soc. 2024, 146, 27417. 39177778 10.1021/jacs.4c07061

[14] A. Zhu , L. Qiao , K. Liu , G. Gan , C. Luan , D. Lin , Y. Zhou , S. Bu , T. Zhang , K. Liu , T. Song , H. Liu , H. Li , G. Hong , W. Zhang , Nat. Commun. 2025, 16, 1880. 39987094 10.1038/s41467-025-57056-6 PMC11846950

[15] a) D. He , X. Song , W. Li , C. Tang , J. Liu , Z. Ke , C. Jiang , X. Xiao , Angew. Chem., Int. Ed. 2020, 59, 6929; 10.1002/anie.202001681 32100367

[16] a) L. Qiao , D. Liu , A. Zhu , J. Feng , P. Zhou , C. Liu , K. W. Ng , H. Pan , Appl. Catal. B 2024, 340, 123219;

[17] Y. Deng , A. D. Handoko , Y. Du , S. Xi , B. S. Yeo , ACS Catal. 2016, 6, 2473.

[18] J. A. Koza , C. M. Hull , Y.‐C. Liu , J. A. Switzer , Chem. Mater. 2013, 25, 1922.

[19] Y. Wang , H. Li , W. Zhou , X. Zhang , B. Zhang , Y. Yu , Angew. Chem., Int. Ed. 2022, 61, 202202604. 10.1002/anie.202202604 35231157

[20] C. Song , Q. Zhan , F. Liu , C. Wang , H. Li , X. Wang , X. Guo , Y. Cheng , W. Sun , L. Wang , J. Qian , B. Pan , Angew. Chem., Int. Ed. 2022, 61, 202200406. 10.1002/anie.202200406 35128779

[21] W. Zhu , F. Yao , Q. Wu , Q. Jiang , J. Wang , Z. Wang , H. Liang , Energy Environ. Sci. 2023, 16, 2483.

[22] W. Gao , K. Xie , J. Xie , X. Wang , H. Zhang , S. Chen , H. Wang , Z. Li , C. Li , Adv. Mater. 2023, 35, 2202952. 10.1002/adma.202202952 36871207

[23] C. Ma , H. Zhang , J. Xia , X. Zhu , K. Qu , F. Feng , S. Han , C. He , X. Ma , G. Lin , W. Cao , X. Meng , L. Zhu , Y. Yu , A. L. Wang , Q. Lu , J. Am. Chem. Soc. 2024, 146, 20069. 38984787 10.1021/jacs.4c04023

[24] H. Sun , L. Chen , Y. Lian , W. Yang , L. Lin , Y. Chen , J. Xu , D. Wang , X. Yang , M. H. Rummerli , J. Guo , J. Zhong , Z. Deng , Y. Jiao , Y. Peng , S. Qiao , Adv. Mater. 2020, 32, 2006784. 10.1002/adma.202006784 33184955

[25] G. Zhang , G. Wang , Y. Wan , X. Liu , K. Chu , ACS Nano 2023, 17, 21328. 37870919 10.1021/acsnano.3c05946

[26] Y. Li , C. Wang , L. Yang , W. Ge , J. Shen , Y. Zhu , C. Li , Adv. Energy Mater. 2023, 14, 2303863.

[27] S. Liang , X. Teng , H. Xu , L. Chen , J. Shi , Angew. Chem., Int. Ed. 2024, 63, 202400206. 10.1002/anie.202400206 38253953

[28] a) W. Liao , J. Wang , G. Ni , K. Liu , C. Liu , S. Chen , Q. Wang , Y. Chen , T. Luo , X. Wang , Y. Wang , W. Li , T. S. Chan , C. Ma , H. Li , Y. Liang , W. Liu , J. Fu , B. Xi , M. Liu , Nat. Commun. 2024, 15, 1264; 38341446 10.1038/s41467-024-45534-2 PMC10858923

[29] L. Wang , M. Ma , C. Zhang , H. H. Chang , Y. Zhang , L. Li , H. Y. Chen , S. Peng , Angew. Chem., Int. Ed. 2024, 63, 202317220. 10.1002/anie.202317220 38153674

[30] Q. Hu , K. Yang , O. Peng , M. Li , L. Ma , S. Huang , Y. Du , Z. X. Xu , Q. Wang , Z. Chen , M. Yang , K. P. Loh , J. Am. Chem. Soc. 2024, 146, 668. 38154089 10.1021/jacs.3c10516

[31] a) L. Barrera , R. Silcox , K. Giammalvo , E. Brower , E. Isip , R. Bala Chandran , ACS Catal. 2023, 13, 4178;

[32] R. Liu , H. Zhao , X. Zhao , Z. He , Y. Lai , W. Shan , D. Bekana , G. Li , J. Liu , Environ. Sci. Technol. 2018, 52, 9992. 30067342 10.1021/acs.est.8b02740

[33] W. Liu , M. Xia , C. Zhao , B. Chong , J. Chen , H. Li , H. Ou , G. Yang , Nat. Commun. 2024, 15, 3524. 38664388 10.1038/s41467-024-47765-9 PMC11045753

[34] J. Wei , S. N. Qin , J. L. Liu , X. Y. Ruan , Z. Guan , H. Yan , D. Y. Wei , H. Zhang , J. Cheng , H. Xu , Z. Q. Tian , J. F. Li , Angew. Chem., Int. Ed. 2020, 59, 10343. 10.1002/anie.202000426 32207867

[35] W. He , J. Zhang , S. Dieckhofer , S. Varhade , A. C. Brix , A. Lielpetere , S. Seisel , J. R. C. Junqueira , W. Schuhmann , Nat. Commun. 2022, 13, 1129. 35236840 10.1038/s41467-022-28728-4 PMC8891333

[36] K. Yang , S. H. Han , C. Cheng , C. Guo , T. Li , Y. Yu , J. Am. Chem. Soc. 2024, 146, 12976. 38567925 10.1021/jacs.3c13517

[37] F. Y. Chen , Z. Y. Wu , S. Gupta , D. J. Rivera , S. V. Lambeets , S. Pecaut , J. Y. T. Kim , P. Zhu , Y. Z. Finfrock , D. M. Meira , G. King , G. Gao , W. Xu , D. A. Cullen , H. Zhou , Y. Han , D. E. Perea , C. L. Muhich , H. Wang , Nat. Nanotechnol. 2022, 17, 759. 35501378 10.1038/s41565-022-01121-4

[38] a) X. Wang , J. J. Wang , H. Hu , C. Yin , L. Y. Chang , Y. Zhu , J. Wang , M. Yang , Adv. Mater. 2025, 37, 2504505; 10.1002/adma.202504505 40304534

[39] a) J. Y. Fang , Q. Z. Zheng , Y. Y. Lou , K. M. Zhao , S. N. Hu , G. Li , O. Akdim , X. Y. Huang , S. G. Sun , Nat. Commun. 2022, 13, 7899; 36550156 10.1038/s41467-022-35533-6 PMC9780304

[40] J. Dai , Y. Tong , L. Zhao , Z. Hu , C. T. Chen , C. Y. Kuo , G. Zhan , J. Wang , X. Zou , Q. Zheng , W. Hou , R. Wang , K. Wang , R. Zhao , X. K. Gu , Y. Yao , L. Zhang , Nat. Commun. 2024, 15, 88. 38167739 10.1038/s41467-023-44469-4 PMC10762114

[41] H. Niu , Z. Zhang , X. Wang , X. Wan , C. Shao , Y. Guo , Adv. Funct. Mater. 2020, 31, 2008533.

[42] K. Liu , H. Yang , Y. Jiang , Z. Liu , S. Zhang , Z. Zhang , Z. Qiao , Y. Lu , T. Cheng , O. Terasaki , Q. Zhang , C. Gao , Nat. Commun. 2023, 14, 2424. 37105957 10.1038/s41467-023-38018-2 PMC10140298

[43] A. Popova , R. Rattanakom , Z.‐Q. Yu , Z. Li , K. Nakagawa , T. Fujioka , Water Res. 2023, 244, 120484. 37611359 10.1016/j.watres.2023.120484

[44] a) R. Zhang , Y. Guo , S. Zhang , D. Chen , Y. Zhao , Z. Huang , L. Ma , P. Li , Q. Yang , G. Liang , C. Zhi , Adv. Energy Mater. 2022, 12, 2103872;

