# Supplementary material for: Tuning Active Hydrogen on Reconstructed RuO2/Co(OH)2 Catalysts for Selective Ammonia Synthesis
Source: Adv Mater. 2025 Sep 25;38(3):e15346. doi: 10.1002/adma.202515346 (PMC12801363; doi:10.1002/adma.202515346)
Supplement: Supplementary file 1 — Supporting Information [file ADMA-38-e15346-s001.docx]

Supporting Information

Tuning Active Hydrogen on Reconstructed RuO_2_/Co(OH)_2_ Catalysts for Selective Ammonia Synthesis

*Anquan Zhu ^1^, Heng Liu ^*,2,3^, Lulu Qiao ^4^, Bin Liu ^*,5^, Kunlun Liu ^1^, Chuhao Luan ^1^, Kai Liu ^1^, Yin Zhou ^1^, Dewu Lin ^6^, Guoqiang Gan ^7^, Jiapei Li ^1^, Guo Hong ^*,1,8^, Wenjun Zhang ^*,1,8^*

^1^ Dr. A. Q. Zhu, K. L. Liu, C. H. Luan, Dr. K. Liu, Dr. Y. Zhou, J. P. Li, Prof. G. Hong, Prof. W. J. Zhang

Department of Materials Science and Engineering, & Center of Super-Diamond and Advanced Films, City University of Hong Kong, 83 Tat Chee Avenue, Kowloon, Hong Kong, China

Email: [apwjzh@cityu.edu.hk](mailto:apwjzh@cityu.edu.hk) (W. J. Zhang); [guohong@cityu.edu.hk](mailto:guohong@cityu.edu.hk) (G. Hong)

^2, 3^ Dr. H. Liu

Advanced Institute for Materials Research (WPI-AIMR), Tohoku University, Sendai 980-8577, Japan; State Key Laboratory of Powder Metallurgy, Central South University, Changsha, China

Email: [heng.liu.e1@tohoku.ac.jp](mailto:heng.liu.e1@tohoku.ac.jp) (H. Liu)

^4^ Dr. L. L. Qiao

Shenzhen Key Laboratory of Micro/Nano-Porous Functional Materials (SKLPM), Department of Chemistry, Department of Materials Science and Engineering and SUSTech-Kyoto University Advanced Energy Materials Joint Innovation Laboratory (SKAEM-JIL), Southern University of Science and Technology (SUSTech), Shenzhen, 518055 China

^5^ Prof. Bin Liu

State Key Laboratory of Chemical Resource Engineering, Beijing Advanced Innovation Center for Soft Matter Science and Engineering, College of Chemistry, Beijing University of Chemical Technology, Beijing 100029, P. R. China

Email: [binliu@buct.edu.cn](mailto:binliu@buct.edu.cn) (B. Liu)

^6^ Dr. D. W. Lin

State Key Laboratory of Advanced Waterproof Materials, School of Advanced Materials, Peking University, Shenzhen Graduate School, Shenzhen 518055, China

^7^ Dr. G. Q. Gan

Key Laboratory of Subsurface Hydrology and Ecological Effects in Arid Region, Ministry of Education, School of Water and Environment, Changan University, Xi'an, 710064, China

^8^ Prof. G. Hong, Prof. W. J. Zhang

The Shenzhen Research Institute, City University of Hong Kong, Shenzhen 518057, China

# Experimental Section

## 1.1. Materials Characterization

The phase components were determined by x-ray powder diffraction patterns on an x-ray diffractometer (XRD; D/max 2550, Rigaku Corporation) with Cu *Kα* radiation (*λ* = 0.15405 nm). The Raman spectroscopy was performed on a WITec alpha300 R Raman microscope with a laser excitation wavelength of 532 nm. Morphologies and structures were investigated by a field emission scanning electron microscope (Thermal Fisher Quattro S Environmental SEM) with an acceleration voltage of 15 kV and transmission electron microscopy (TEM, JEM-2100F JEOL Ltd. Japan) with an acceleration voltage of 200 kV. Surface chemical structures were analyzed with x-ray photoelectron spectroscopy (ESCALAB Xi^+^, Thermo Fisher Scientific), and the binding energies of all elements were calibrated through the C 1*s* peak (Binding Energy=284.8 eV) as the standard. The content of Co and Ru in the RuO_2_/Co_3_O_4_ composites and the reconstructed RuO_2_/Co(OH)_2_ catalyst were identified using inductively coupled plasma optical emission spectrometer (ICP-OES, Optima 8000).

## 1.2. Electrodes Preparation

Prior to being used as a current collector, carbon paper (CP) was cut into small pieces with a 0.75 × 1 cm^2^ geometric area. These pieces were then sonicated and washed with ethanol, aqua regia, and deionized water to eliminate surface impurities. After drying in a vacuum oven, these CP pieces were saved for further use. Meanwhile, 10 mg of catalysts (or 20 wt.% Pt/C catalyst) and 30 μL of 5 wt.% Nafion solution were added into 470 μL of isopropanol. After continuous sonication for 1 hour, the mixture formed a uniformly dispersed ink. Using a pipette, the catalyst ink was transferred and coated onto the surface of the small CP pieces (loading area: 0.5 × 1 cm^2^). The working electrodes were successfully made after drying at 60 °C for 1 hour in a vacuum oven, with a loading mass measured to be approximately 1.5 mg cm^−2^.

## 1.3. Electrochemical Measurements

All the electrochemical tests were performed in an H-type cell linked with three-electrode system controlled by a Chenhua electrochemical workstation (CHI 660E). A commercial Pt plate and a Hg/HgO electrode filled with 1 M KOH solution serve as the counter electrode and reference electrode, respectively. The catholytes varied in the concentrations of KOH (0-2 M) and KNO_3_ (0-500 mM), while the electrolyte in the anodic chamber was 1 M KOH. Before the measurements, the catholytes were purified with high-purity argon. Cyclic voltammetry (CV) curves were carried out at various scan rates (20, 40, 60, 80, and 100 mV s^−1^) at a non-faradaic potential range. The electrochemical double-layer capacitances (*C*_dl_) were obtained by converting the CV curves. Linear sweep voltammetry (LSV) curves with 80% *iR*-compensation were carried out at a scan rate of 5 mV s^−1^ in 1 M KOH with or without 100 mM NO_3_^−^. The open-circuit potential (OCP) measurements of various catalysts were conducted in 1 M KOH without and with different concentrations of nitrate. Chronoamperometry tests were carried out at various potentials for 1 hour, and the reacted electrolytes with liquid products were collected for chromogenic analysis. The potentials in this study were calibrated to the reversible hydrogen electrode (RHE) potential according to the Nernst equation S1^[1]^:

$E_{\mathrm{RHE}}=E_{Hg/HgO}+E_{Hg/HgO}^{0}+0.059\times\mathrm{pH}$ (Equation S1)

Where $E_{Hg/HgO}$ is measured potential; $E_{Hg/HgO}^{0}$is the standard potential (0.098 V) of Hg/HgO electrode in 1 M KOH at room temperature.

## 1.4. *In*-*situ* Electrochemical Impedance Spectroscopy (EIS) Measurements

The *in-situ* electrochemical impedance spectroscopy (EIS) measurements were performed over a potential range from 0 to −0.4 V vs. RHE. The frequency range for these measurements was from 100 kHz to 0.01 Hz, with an AC voltage amplitude of 5 mV. The electrolytes used for these measurements were 1 M KOH with and without 100 mM NO_3_^−^.

## 1.5. NH_4_^+^ Quantification by UV-vis Test

Ammonia (NH_4_^+^) concentration was determined using the indophenol blue method. In a typical test, 20 µL of the electrolyte, collected after electrolysis at different potentials, was mixed with 2 mL deionized (DI) water. Subsequently, 2 mL of 1 M NaOH solution containing 5 wt.% salicylic acid and 5 wt.% sodium citrate, 1 mL of 0.05 M NaClO solution, and 200 µL of 1 wt.% sodium nitroferricyanide were sequentially added to the mixture. The NH_4_^+^ concentration was determined by UV-vis spectroscopy at 655 nm. The absorbance spectra for NH_4_^+^ concentration were calibrated using standard NH_4_Cl solution with varying concentrations (0-500 mg L^−1^ NH_4_^+^).

## 1.6. NO_2_^−^ Determination by UV-vis Test

Nitrite was analyzed using the Griess test. To prepare the Griess reagent, 0.8 g of N-(1-naphthyl) ethyldiamine dihydrochloride, 16 g of sulfonamide, and 80 mL of H_3_PO_4_ were dissolved in 400 mL of DI water. In a typical measurement procedure, 1 mL of Griess reagent was mixed with 3 mL of DI water and 100 µL of catholyte. The sulfonamide reacts with NO_2_^−^ to form the diazonium salt, which then reacts with the amine to form an azo dye. The concentration-dependent absorbance spectra were calibrated using standard KNO_2_ solutions with varying concentrations (0-200 mg L^−1^ NO_2_^−^).

## 1.7. Calculation of The Faradaic Efficiencies (FEs), Yield Rate, and The Half-Cell Energy Efficiencies (EEs) for Liquid Products

For the calculation of Faradaic efficiencies (FEs) of NH_3_^[2]^:

${FE}_{\mathrm{NH}_{3}}={(8\times F\times c(NH_{3})\times V)}/{(M_{\mathrm{NH}_{3}}}\times Q)\times100\%$ (Equation S2)

The yield rate for NH_3_:

${Yield rate}_{NH_{3}}={(c(NH_{3})\times V)}/{(t\times S)}$ (Equation S3)

The half-cell EEs were calculated as follows:

${EE}_{\mathrm{NH}_{3}}={(1.23-E_{\mathrm{NH}_{3}}^{0})\times{FE}_{\mathrm{NH}_{3}}}/{(1.23-E)}$ (Equation S4)

The FEs for NO_2_^−^:

${FE}_{\mathrm{NO}_{2}^{-}}={(2\times F\times c(NO_{2}^{-})\times V)}/{(M_{\mathrm{NO}_{2}^{-}}}\times Q)\times100\%$ (Equation S5)

Where *F* is the Faradaic constant (96485 C mol^−1^); $c(NH_{3})/c\left( NO_{2}^{-} \right)$is the measured concentration of produced NH_3_ or NO_2_^−^; *V* is the electrolyte’s volume (30 mL); $M_{\mathrm{NH}_{3}}$/$M_{\mathrm{NO}_{2}^{-}}$ is the molar mass of NH_3_ or NO_2_^−^; *Q* is the total charge passing through the electrode; *t* is the electrolysis time; *S* is the geometric area of the working electrode (cm^2^); 1.23 V represents the equilibrium potential of water oxidation; *E* is the applied potentials relative to RHE with 80% *iR*-compensation;$E_{\mathrm{NH}_{3}}^{0}$is the equilibrium potentials of electrochemical nitrate reduction (eNO_3_RR) to ammonia. The theoretical equilibrium potential (vs. SHE) of eNO_3_RR can be calculated based on the following equation:

$E_{0}^{0}=\frac{\sum\upsilon_{1}\mu_{1}-\upsilon_{2}\mu_{2}}{23060n}$ (Equation S6)

Where *ν*_1_ and *ν*_2_ are the coefficients of reactants and products (*e. g.*, the *ν*_1_ of H_2_O is 6, while the *ν*_2_ of OH^−^ is 9), respectively. *µ*_1_ and *µ*_2_ are the energies of the reactant and product molecules, respectively (all energy values can be found in “Atlas of Electrochemical Equilibria in Aqueous Solutions”). The final value of $E_{0}^{0}$for the eNO_3_RR to ammonia is calculated to be −0.1314 V (vs. SHE). Hence, $E_{NH_{3}}^{0}$can be calculated by this equation:

$E_{NH_{3}}^{0}=E_{0}^{0}+\frac{0.0591}{n}ln\frac{[NO_{3}^{-}]}{{[OH^{-}]}^{9}}$ (Equation S7)

Where [NO_3_^−^] is the concentration of nitrate in the electrolyte, ranging from 0 to 500 mM; [OH^−^] is the concentration of hydroxide ions in the electrolyte, ranging from 0 to 2 M; *n* is the number of electrons transferred during the eNO_3_RR. Thus the $E_{NH_{3}}^{0}$ can be calculated in accordance with different concentration combinations of NO_3_^−^ and OH^−^.

## 1.8. Gaseous Hydrogen Product Measurement

The electrochemical hydrogen production was evaluated in an H-type cell with a Chenhua CHI 660E electrochemical workstation. A Pt plate and an Hg/HgO electrode served as counter and reference electrode, respectively. The electrolytes in the cathode and anode chambers were 1 M KOH with and without 100 mM NO_3_^−^, respectively. The electrolytes were purged with high-purity argon for 30 minutes to remove dissolved O_2_ and H_2_. The volume of electrolytes in each chamber were maintained at 30 mL. During the constant potential test, high-purity argon was continuously applied to the cathode chamber at a flow rate of 50 sccm. The gas outlet was connected to a gas chromatography (GC, Shimadzu GC-2010 plus) for online analysis of the gas products. The produced hydrogen was detected using a thermal conductivity detector (TCD). The FEs of H_2_ were calculated as follows^[3]^:

$FE_{H_{2}}=\frac{2FP_{0}}{RT}\times\frac{1}{\alpha}\times peak area\times\left( flow rate \right)\times100\%$ (Equation S8)

Where *F* represents the Faraday constant (96485 C mol^−1^); *R* is the gas constant (8.314 J mol^−1^ k^−1^); *P*_0_ is the ambient pressure (101.325 kPa); *T* is the room temperature (298.15 K); *α* is the conversion factor for H_2_ on the calibration of the GC with a standard sample.

## 1.9. ^15^N Isotope-Labelling Experiments and ^1^H Nuclear Magnetic Resonance (NMR) Measurements

The K^15^NO_3_ (99 atm. %) and K^14^NO_3_ were used as the nitrogen sources for isotopic labeling nitrate reduction experiments to identify the source of ammonia. The electrolyte consists of 1 M KOH with 100 mM K^15^NO_3_ and K^14^NO_3_. After the eNO_3_RR at −0.3 vs. RHE, 400 μL of the reacted electrolyte containing the generated ^15^NH_4_^+^ and ^14^NH_4_^+^ products was extracted and adjusted to approximately pH=2 with 80 μL of concentrated HCl for further quantification. The quantification was performed using ^1^H NMR (300 MHz) with H_2_O as the solvent, 50 μL of DMSO-*d*_6_, and 10 μL of maleic acid (5 g L^−1^) as the internal standard.

## 1.10. Electron Spin Resonance (ESR) Measurements

5, 5-dimethyl-1-pyrroline N-oxide (DMPO) was employed to capture the instable hydrogen radical, forming the DMPO-H adduct for ESR spectra analysis. Electrolysis was conducted for 20 minutes at −0.3 V vs. RHE in an H-type cell, with the electrolyte bubbled with argon. After the reaction, 1 mL of the electrolyte was mixed with 10 μL of DMPO and transferred into a glass tube. The ESR measurement was performed using a Bruker magnettech ESR 5000 spectrometer, operating at a frequency of 9.45 GHz with a sweep width of 200 G and a power of 20 mW.

## 1.11. *In*-*situ* Raman Spectroscopy Measurements

*In*-*situ* Raman spectroscopy measurements were performed using a GOOSE UNION cell, with a Pt wire and an Ag/AgCl electrode served as the counter electrode and reference electrode, respectively. The electrolyte was 1 M KOH with 100 mM NO_3_^−^. The potential- (potential range: 0.1 to −0.7 V vs. RHE) and time-dependent (at −0.3 V) Raman spectroscopy of RuO_2_, Co_3_O_4_, RuO_2_/Co_3_O_4_ precatalysts during eNO_3_RR were recorded to analyze dynamic structural transformations. Additionally, the structural information of water molecule at the catalyst/electrolyte interface was investigated by collecting and analyzing Raman signals during eNO_3_RR operated at −0.3 V vs. RHE.

**1.12. Electrochemical *in*-*situ* Fourier Transform Infrared Spectroscopy (FTIR) Measurements**

*In*-*situ* electrochemical FTIR spectroscopy was conducted in a solution of 1 M KOH containing 100 mM NO_3_^−^. The spectrum obtained at open circuit potential (OCP) serves as the reference. The potential settings range from 0.1 V to −0.5 V vs. RHE, with intervals of −0.1 V.

## 1.13. Online Differential Electrochemical Mass Spectrometry (DEMS) Measurements

For the online DEMS test, the electrolyte consisted of 1 M KOH containing 100 mM NO_3_^−^. The working electrodes were glassy carbon electrodes coated with R-Co(OH)_2_ and R-RuO_2_/Co(OH)_2_ catalysts. A Pt wire and an Ag/AgCl electrode served as the counter electrode and reference electrode, respectively. CV measurements were performed over a potential window ranging from −0.6 V and 0.4 V vs. RHE, with an electrolyte flow rate of 3 mL/min. After completing CV cycling, the measurement was paused for 200 seconds allow for the removal of any possible bubbles on the working electrodes. The signals of various intermediates were collected along during each operation using a Linglu QAS100 mass spectrometer, continuing for a total of eight cycles. The intermediates were carried to the detector using helium as the carrier gas.

## 1.14. Extended Applications

## *1.14.1. The measurement of removing NO_3_^−^ in alkaline wastewater*

The electrolytes were 1 M KOH containing 100, 500, and 1000 ppm of NO_3_^−^. The catalyst was R-RuO_2_-Co(OH)_2_. After conducting catalysis at −0.3 V for 20 hours, the electrolytes were collected for UV-vis measurement.

## *1.14.2. Assembly and performance testing of Zn-NO_3_^−^ battery*

The Zn-NO_3_^−^ battery was assembled and tested in an H-type cell. R-RuO_2_/Co(OH)_2_ was used as cathode, and a polished Zn plate (2 × 1 cm^2^) served as the anode. The catholyte was 1 M KOH with 100 mM NO_3_^−^, while the anodic electrolyte contained 6 M KOH with 0.2 M Zn(Ac)_2_. The cathode and anode were separated by a FUMASEP FBM-PK bipolar membrane with a thickness of approximately 130 μm. The LSV curves were measured from OCP to 0 V vs. Zn/Zn^2+^ with a scan rate of 5 mV s^−1^. The assembled Zn-NO_3_^−^ batteries using various catalysts were operated at current densities ranging from 10 to 50 mA cm^−2^. Liquid products were collected for further UV-vis test to determine yield rates and FEs of NH_3_. Galvanostatic tests were conducted using CHI 660E workstation and a NEWARE battery testing system at room temperature^[4]^.

The power density (*P*) was calculated using the Equation S9:

*P* = *j* × *V* (Equation S9)

Where *j* and *V* are the discharge current density and voltage, respectively.

The electrochemical reactions in the Zn-nitrate battery are shown as follows: ^[4]^ ^[5]^ ^[5b]^ ^[6]^

Discharging process:

Cathodic reaction: NO_3_^−^ + 7H_2_O + 8e^−^→NH_4_OH + 9OH^−^

Anodic reaction: Zn + 2OH^−^→ZnO + H_2_O + 2e^−^

Overall reaction: 4Zn + NO_3_^−^ + 3H_2_O→4ZnO + NH_4_OH + OH^−^

Charging process:

Cathodic reaction: 4OH^−^→O_2_ + 2H_2_O + 4e^−^

Anodic reaction: ZnO + H_2_O + 2e^−^→Zn + 2OH^−^

Overall reaction: ZnO + 2OH^−^→Zn + O_2_ + H_2_O + 2e^−^

Considering that the cathodic and anodic chambers were separated by a bipolar membrane, the electrode potential can be calculated as follows:

$\text{E}_{\text{cathode}}\text{ =}-\frac{\text{1}}{\text{n}\text{F}}\text{(}{\text{∆}\text{G}}_{\text{cat}}\text{+}\text{R}\text{Tln}\frac{{\text{[}\text{OH}\text{]}}^{9}\text{[}\text{NH}_{\text{4}}\text{OH]}}{\text{[}\text{NO}_{\text{3}}\text{]}}\text{)}$ (Equation S10)

$\text{E}_{\text{anode}}\text{=}-\frac{\text{1}}{\text{n}\text{F}}\text{(}{\text{∆}\text{G}}_{\text{ano}}\text{+}\text{R}\text{Tln}\frac{\text{1}}{{\text{[}\text{OH}\text{]}}^{\text{2}}})$ (Equation S11)

Where *n* is the electron transfer number; *F* is the Faraday constant (96485 C mol^−1^); $\text{∆}\text{G}$ is the standard molar Gibbs free energy change of chemical reaction at 298 K (${\text{∆}\text{G}}_{\text{cat}}$=275.4 kJ mol^−1^, ${\text{∆}\text{G}}_{\text{ano}}$=−972.92 kJ mol^−1^); *R* is the gas constant (8.314 J mol^−1^ K^−1^); *T* is the reaction temperature (298 *K*), respectively. Given that the concentrations of [OH^−^] at the cathode and anode are 1 and 6 mol L^−1^, respectively; the concentration of [NO_3_^−^] is 0.1 mol L^−1^; the assumed concentration of [NH_4_OH] is 10^−3^ mol L^−1^ at the cathode. The overall reaction potential *E*_overall_ can be calculated as:

$E_{\mathrm{overall}}=E_{\mathrm{anode}}-E_{\mathrm{cathode}}=1.61 V$ (Equation S12)

## 1.15. Theoretical Calculations

Spin-polarized density functional theory (DFT) calculations were performed using the Vienna ab initio simulation package (VASP)^[7]^, which utilizes the projector augmented wave (PAW) method^[8]^. The electron exchange and correlation interactions were computed using the Generalized Gradient Approximation (GGA) methodology^[9]^, specifically, parameterized by the Revised Perdew–Burke–Ernzerhof (RPBE) functional^[10]^. Kohn-Sham wave functions^[11]^ were expanded in a plane-wave basis^[12]^ set with an energy cutoff of 400 eV. The convergence criteria for geometric relaxation were defined such that the forces acting on each atom were less than 0.05 eV Å^−1^. To separate the adjacent slab models, a 15 Å vacuum layer was selected. The bulk structures, computational parameters for bulk structures, and the Hubbard U values in the DFT+U method^[13]^ were referenced from the Materials Project database^[14]^. Considering lattice matching and experimental observations, Co(OH)_2_ (001) and rutile RuO_2_ (101) were selected for the adsorption study and nanocomposite building, which are realized using the Atomic Simulation Environment (ASE) Libraries^[15]^. For the binding energy calculations, the top two layers were allowed to relax while the remaining layers were fixed to their bulk positions. The computational hydrogen electrode (CHE) method proposed by Nørskov *et al*^[16]^. was employed to consider the energy of H^+^ and e^−^ coupling. Zero-point energy (ZPE) and entropic corrections were derived from a prior study^[17]^ conducted at 298.15 K.

## 1.16. Statistical Analysis

Origin was used as the software for data-processing. The error bars in this work are presented as mean±standard deviation with three individual measurements.

# Figures and Tables


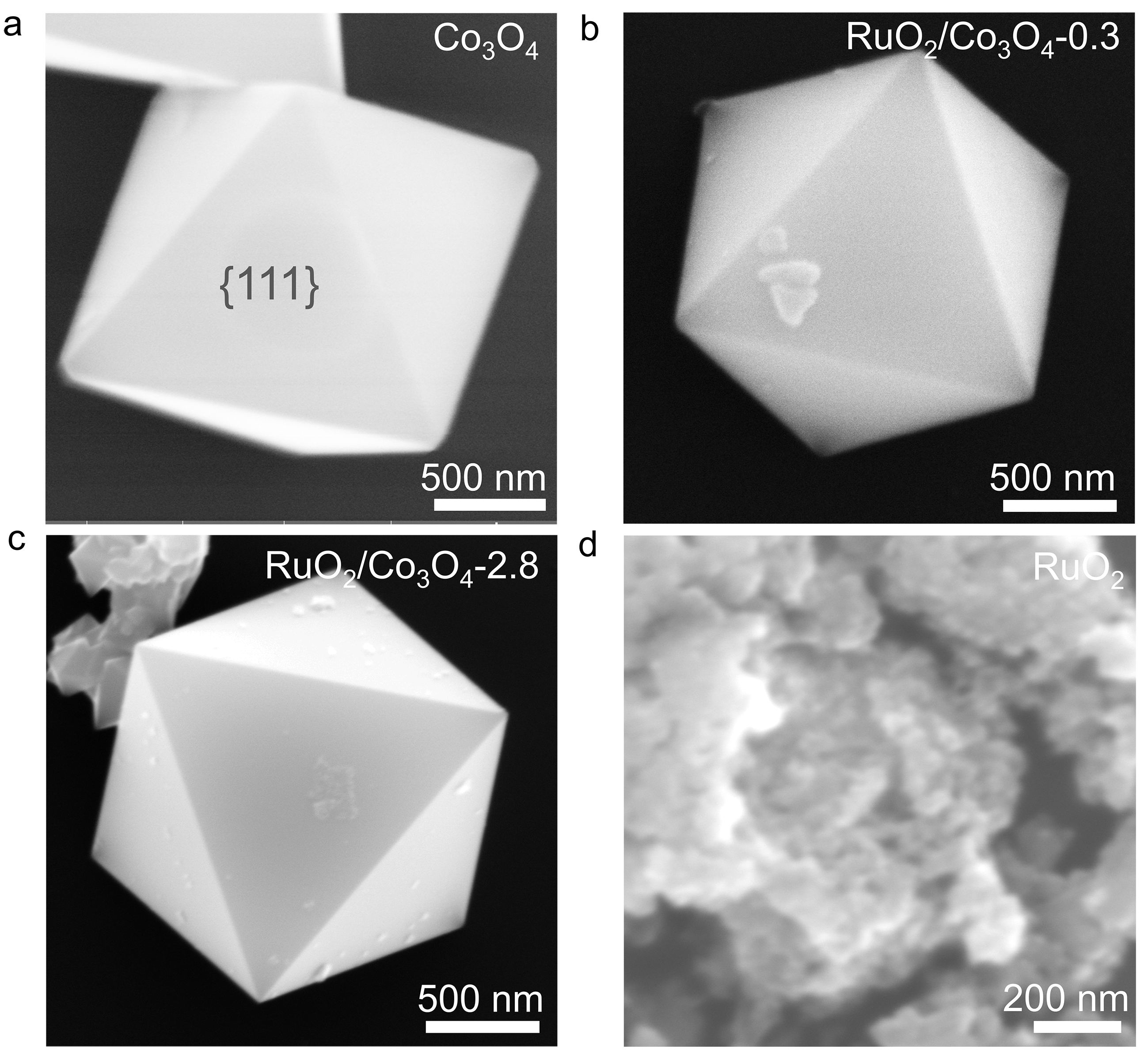


Figure S1. SEM images of (a) Co_3_O_4_, (b) RuO_2_/Co_3_O_4_-0.3, (c) RuO_2_/Co_3_O_4_-2.8, and (d) RuO_2_ precatalysts.


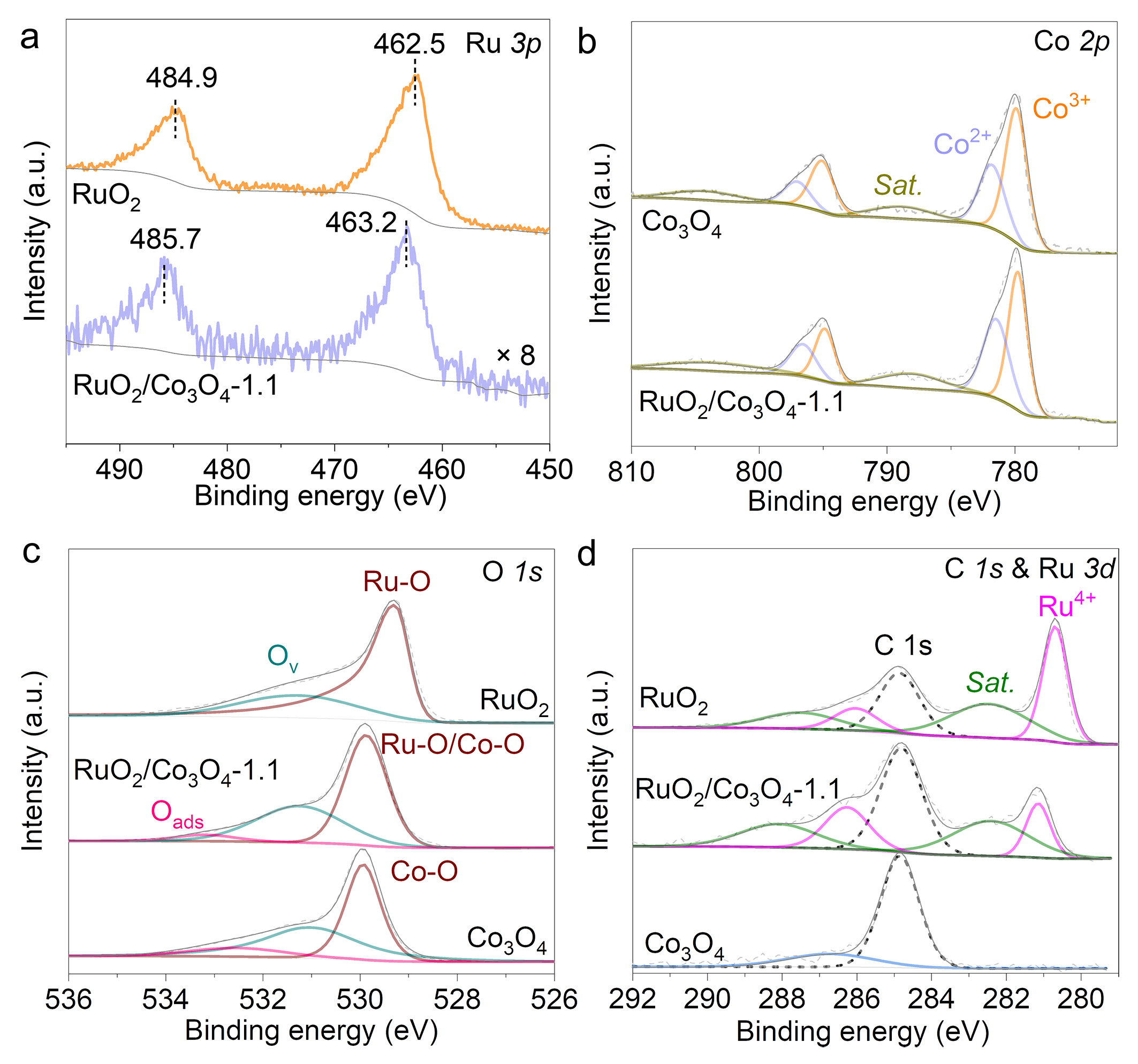


Figure S2. High-resolution XPS spectra of (a) Ru 3*p*, (b) Co 2*p*, (c) O 1*s*, (d) C 1*s* and Ru 3*d* for various precatalysts.


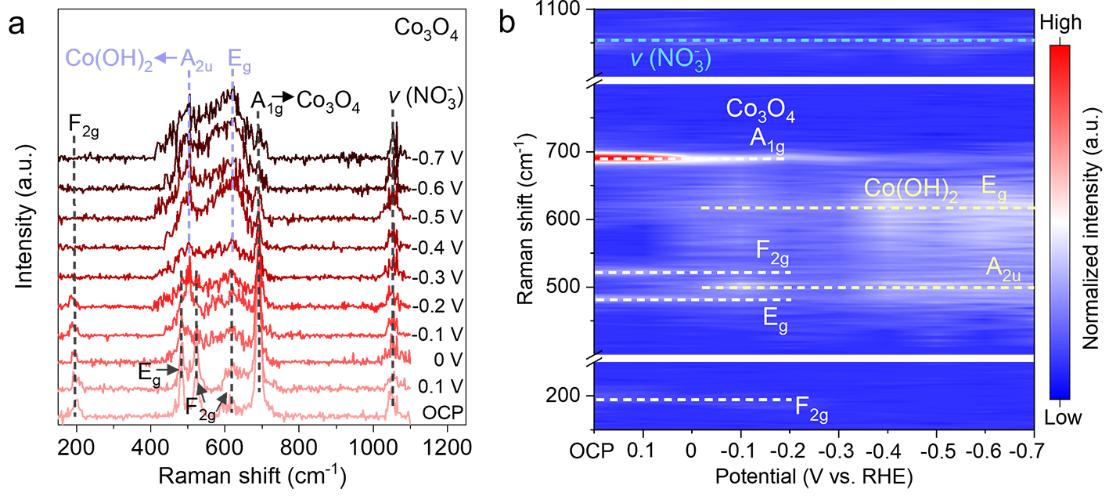


Figure S3. (a, b) Potential-dependent *in*-*situ* Raman spectra of Co_3_O_4_ precatalyst during eNO_3_RR, with the electrolyte as 1 M KOH containing 100 mM NO_3_^−^.

For spinel Co_3_O_4_, as the applied potential became more negative, the intensity of typical Raman vibrations associated with *A*_1g_ (690 cm^−1^), *F*_2g_ (617 cm^−1^), *F*_2g_ (520 cm^−1^), *E*_g_ (482 cm^−1^), and *F*_2g_ (196 cm^−1^) diminished and eventually disappeared below −0.2 V vs*.* RHE^[18]^. Meanwhile, two broadened Raman vibrations emerged at 506 cm^−1^ and 621 cm^−1^, attributed to the *A*_2u_ and *E*_g_ modes of Co(OH)_2_^[19]^, indicating a phase change from Co_3_O_4_ to Co(OH)_2_ at potentials below −0.2 V (Figure S3).


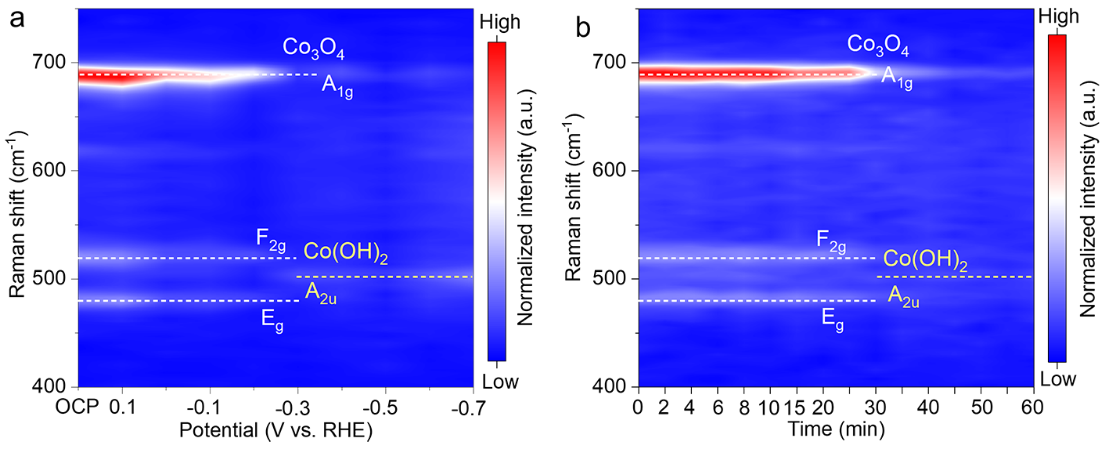


Figure S4. (a) Potential-dependent, and (b) time-dependent *in*-*situ* Raman spectra of RuO_2_/Co_3_O_4_-1.1 precatalyst at −0.3 V vs. RHE during eNO_3_RR, with the electrolyte as 1 M KOH containing 100 mM NO_3_^−^.


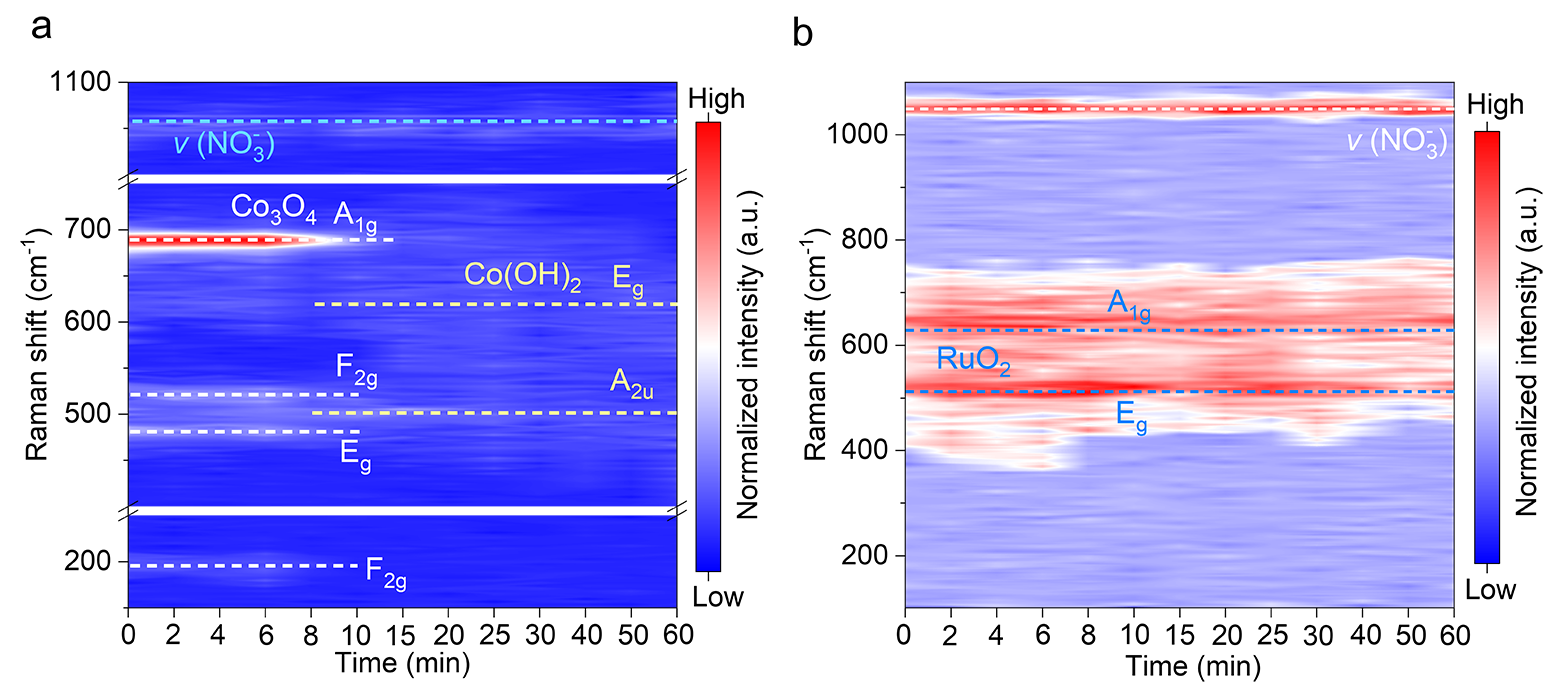


Figure S5. Time-dependent *in*-*situ* Raman spectra of (a) Co_3_O_4_ and (b) RuO_2_ precatalysts at −0.3 V vs. RHE during eNO_3_RR, with the electrolyte as 1 M KOH containing 100 mM NO_3_^−^.


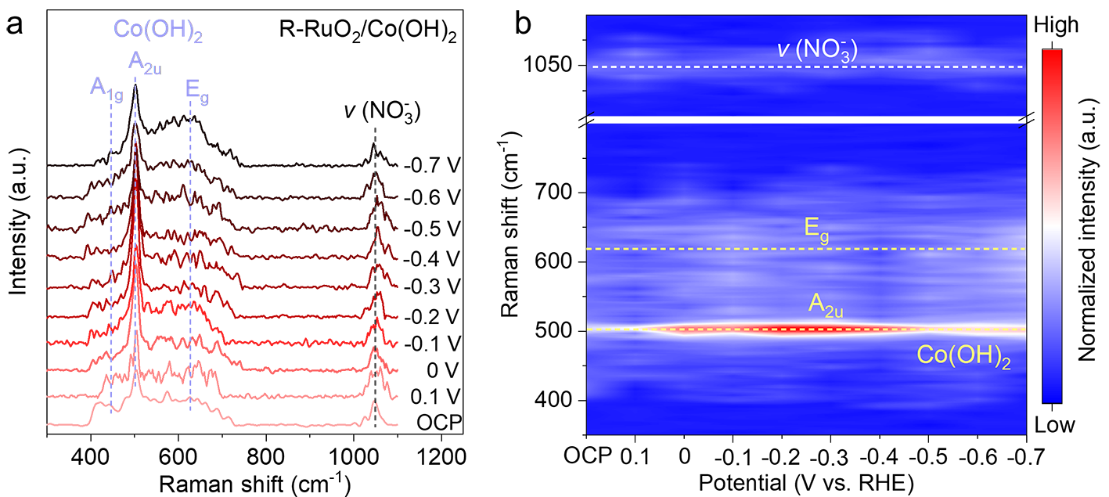


Figure S6. (a, b) Potential-dependent *in*-*situ* Raman spectra of R-RuO_2_/Co(OH)_2_ during eNO_3_RR, with the electrolyte as 1 M KOH containing 100 mM NO_3_^−^.


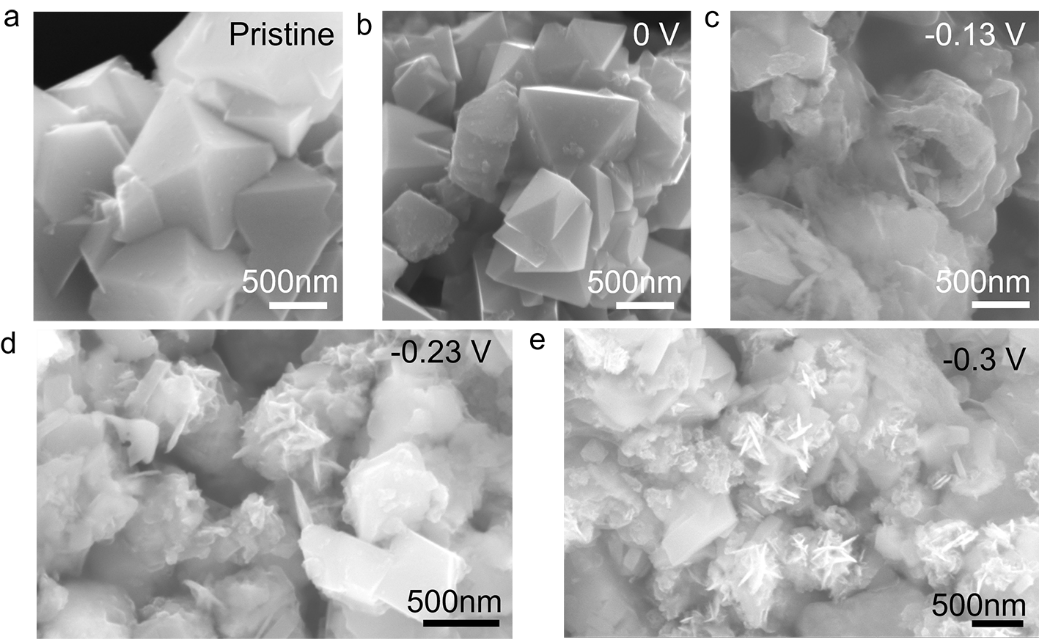


Figure S7. SEM images of (a) RuO_2_/Co_3_O_4_-1.1 and the precatalyst after reconstruction at (b) 0 V, (c) −0.13 V, (d) −0.23 V, and (e) −0.3 V for 1 hour, with the electrolyte as 1 M KOH containing 100 mM NO_3_^−^.


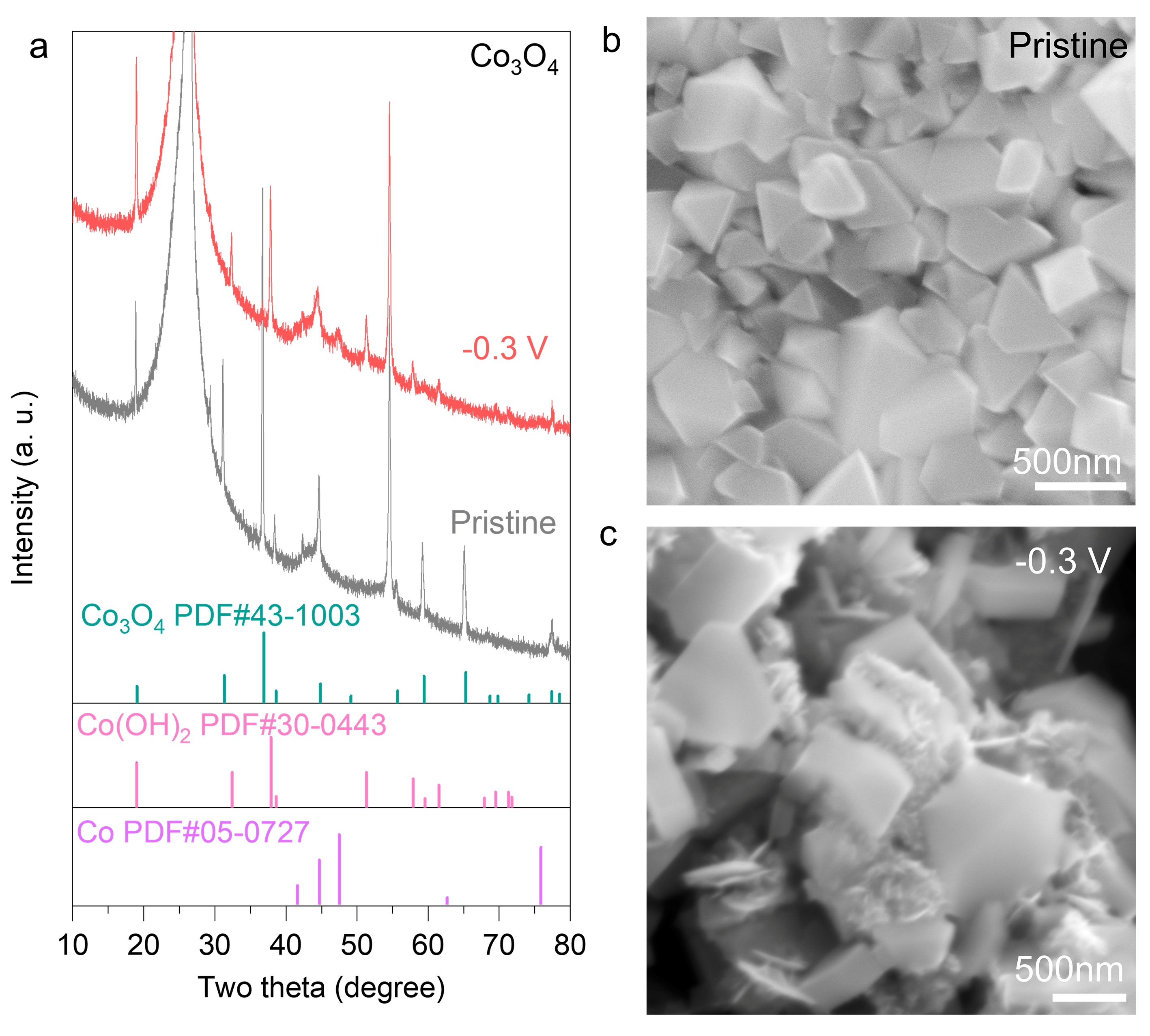


Figure S8. (a) XRD patterns, and (b, c) SEM images of Co_3_O_4_ precatalyst before and after reconstruction at −0.3 V.


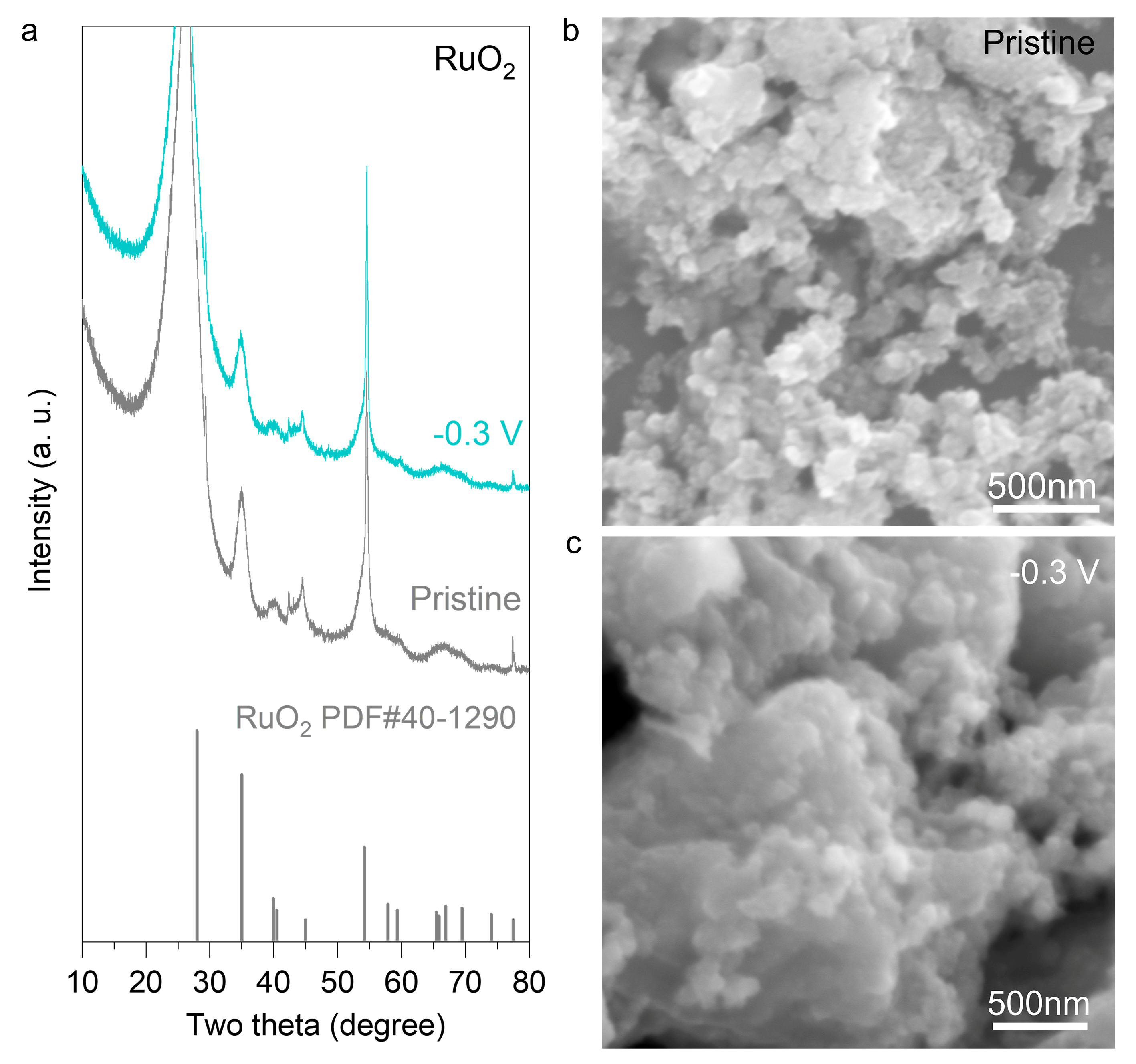


Figure S9. (a) XRD patterns, and (b, c) SEM images of RuO_2_ precatalyst before and after reconstruction at −0.3 V.


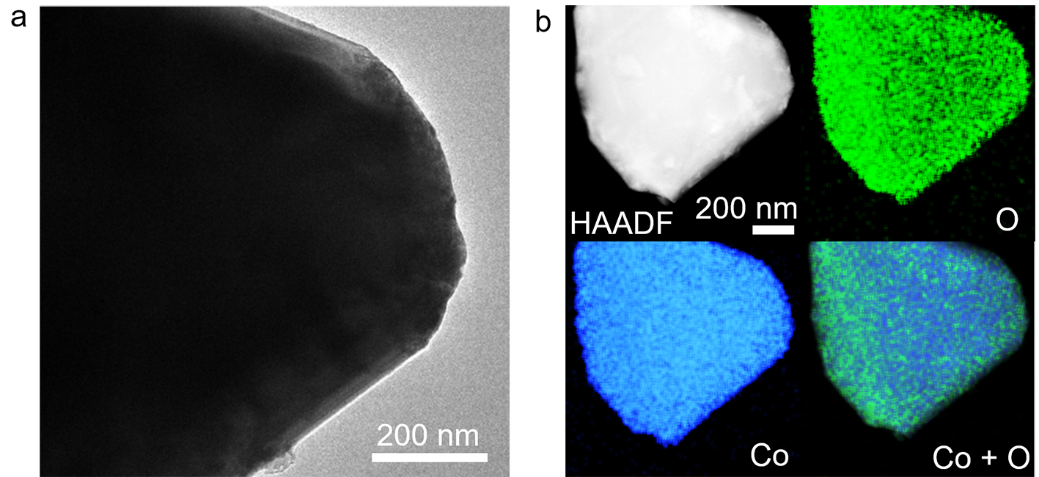


Figure S10. (a) TEM image, and (b) elemental mapping of Co_3_O_4_ precatalyst.


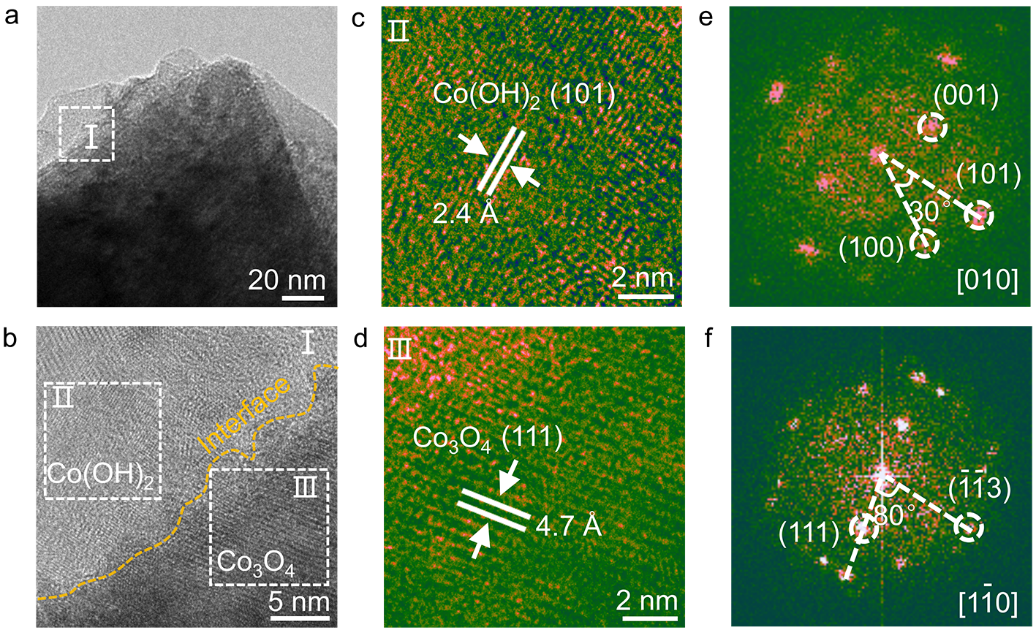


Figure S11. (a) TEM, (b-d) HRTEM images, and (e, f) corresponding FFT patterns of the Co_3_O_4_ precatalyst after reconstruction at −0.3 V.


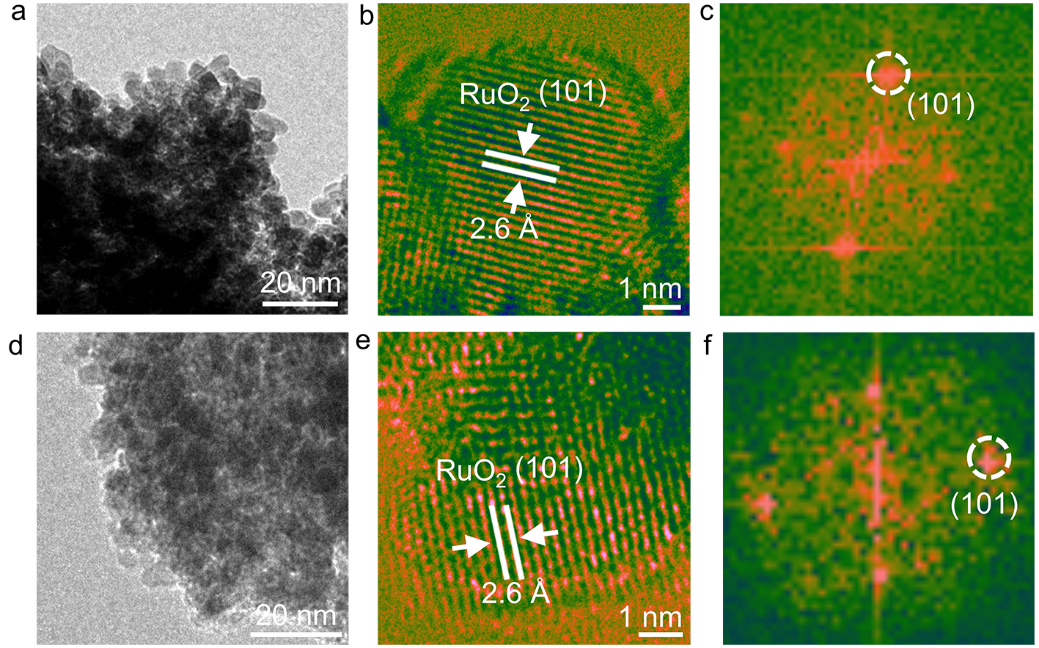


Figure S12. (a) TEM, (b) HRTEM images, and (c) the corresponding FFT pattern of pristine RuO_2_. (d) TEM, (e) HRTEM images, and (f) the corresponding FFT pattern of the reconstructed RuO_2_ at −0.3 V.


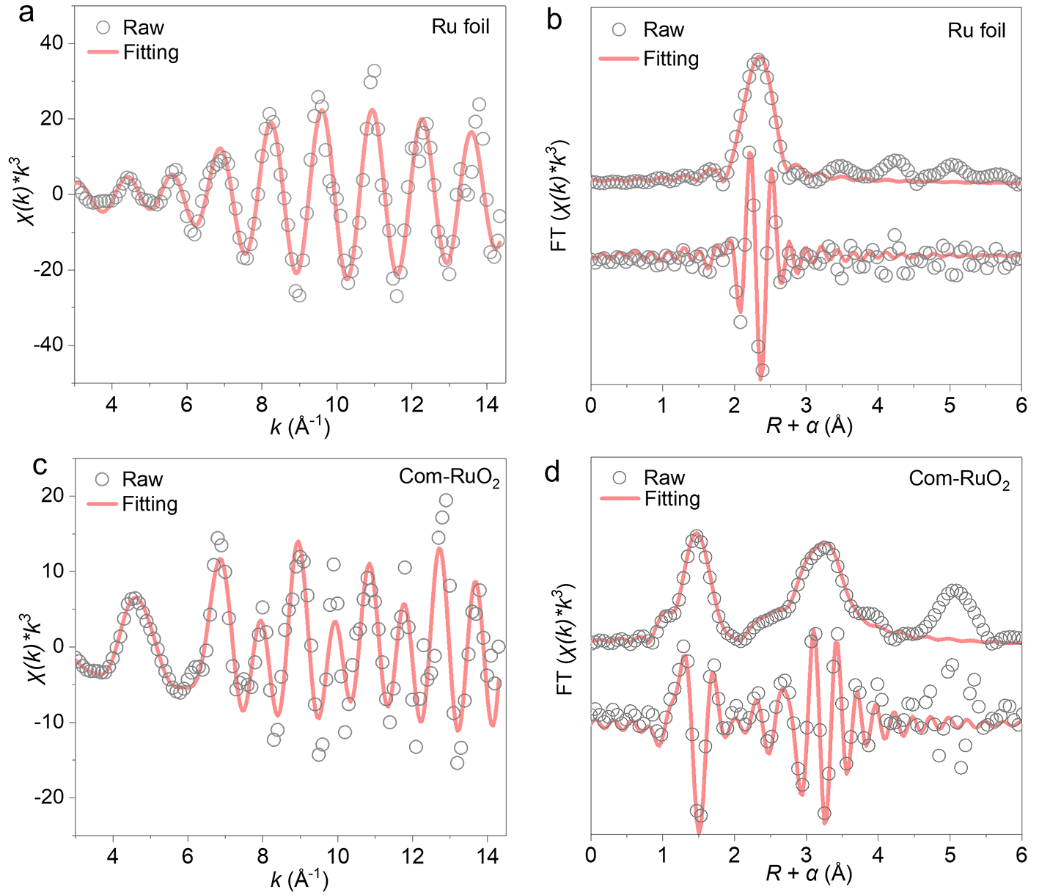


Figure S13. *K*^3^-weighted Ru *K*-edge fitting curves of EXAFS profile in *K*-space and *R*-space of (a, b) Ru foil, and (c, d) commercial RuO_2_.


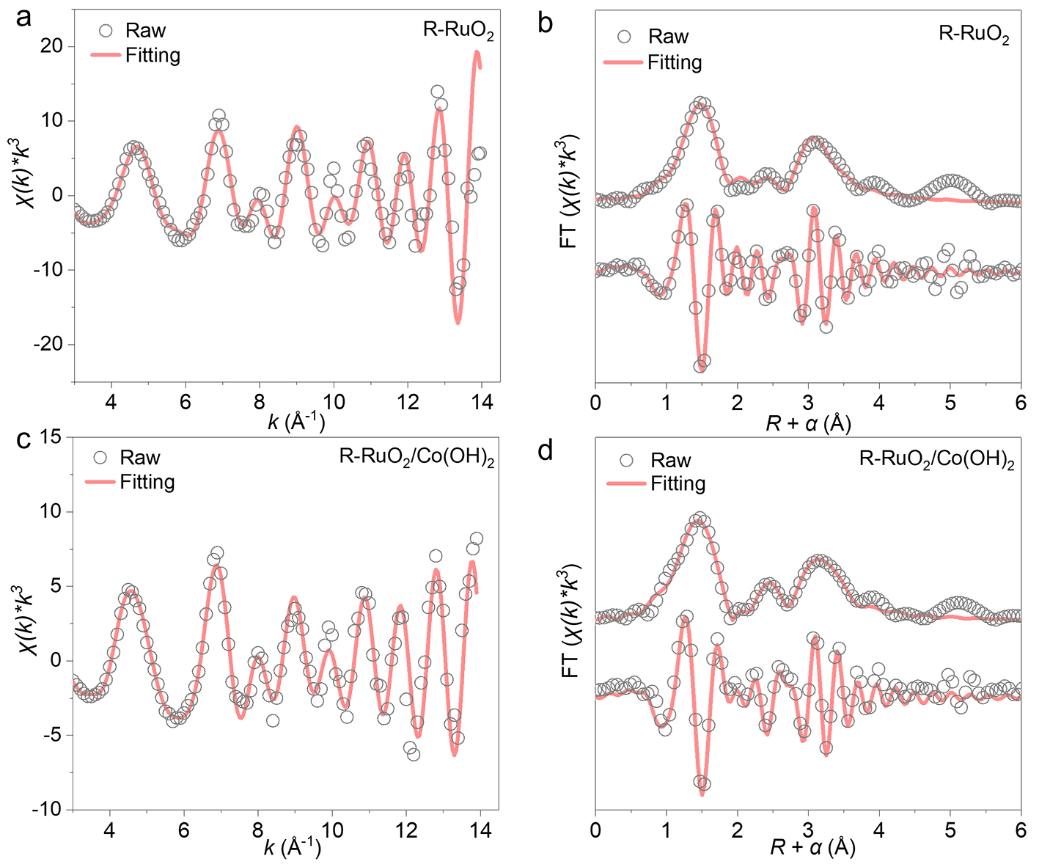


Figure S14. *K*^3^-weighted Ru *K*-edge fitting curves of EXAFS profile in *K*-space and *R*-space of (a, b) R-RuO_2_, and (c, d) R-RuO_2_/Co(OH)_2_.


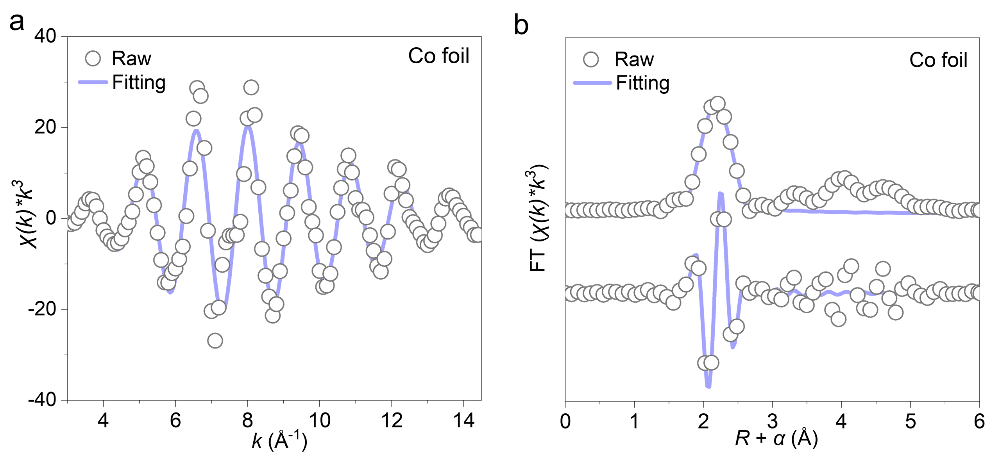


Figure S15. *K*^3^-weighted Co *K*-edge fitting curves of EXAFS profile in (a) *K*-space and (b) *R*-space of Co foil.


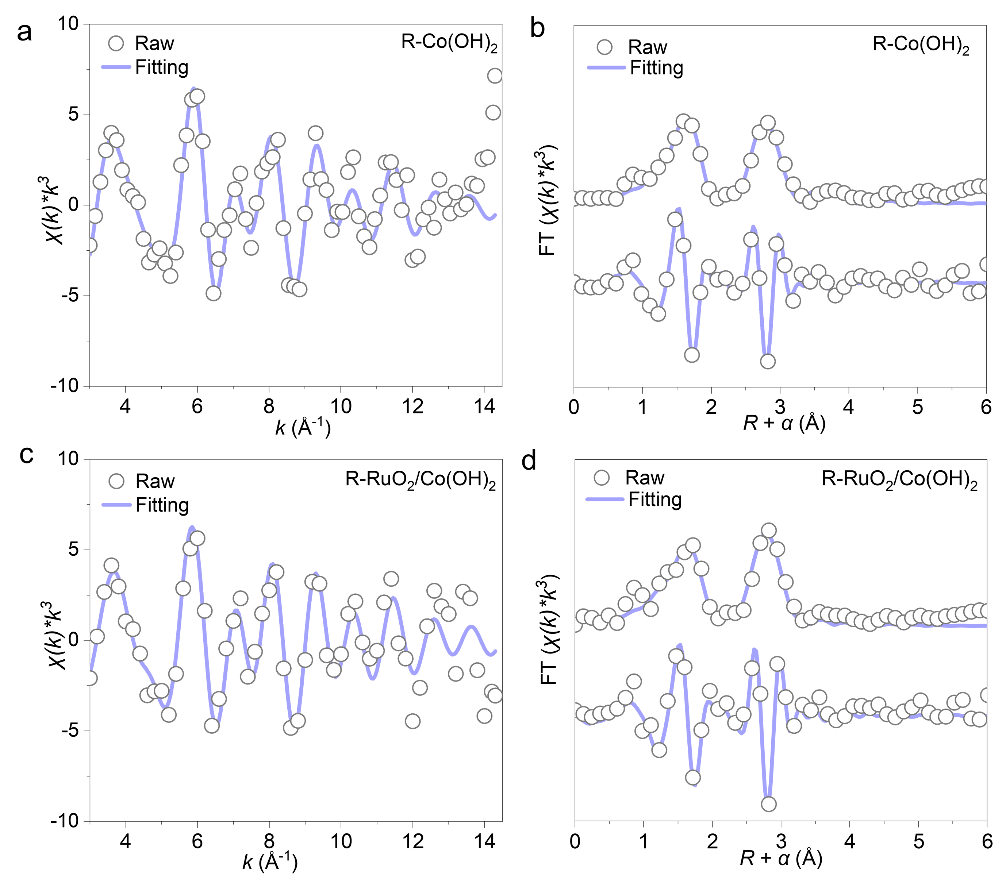


Figure S16. *K*^3^-weighted Co *K*-edge fitting curves of EXAFS profile in *K*-space and *R*-space of (a, b) R-RuO_2_, and (c, d) R-RuO_2_/Co(OH)_2._


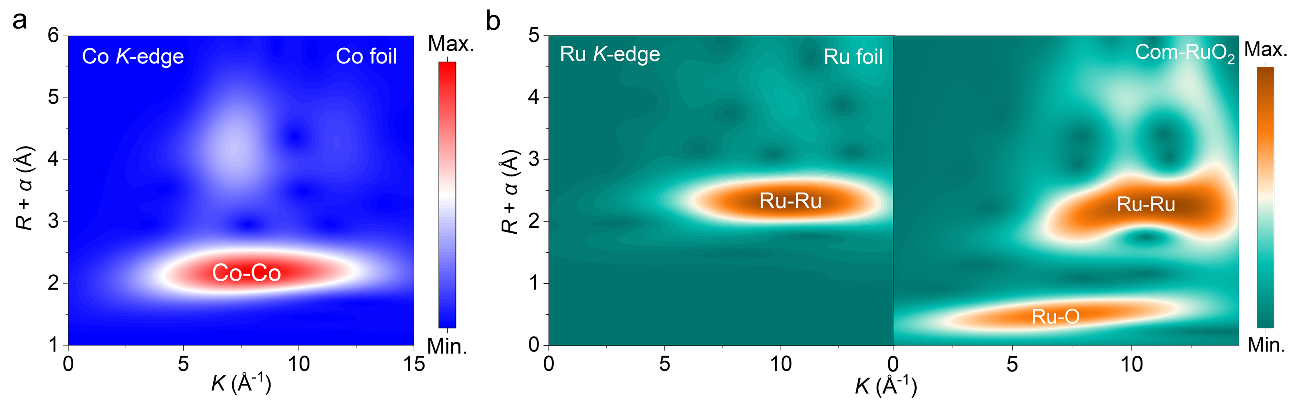


Figure S17. (a) Co *K*-edge WT-EXAFS spectra of Co foil. (b) Ru *K*-edge WT-EXAFS spectra of Ru foil and commercial RuO_2_.


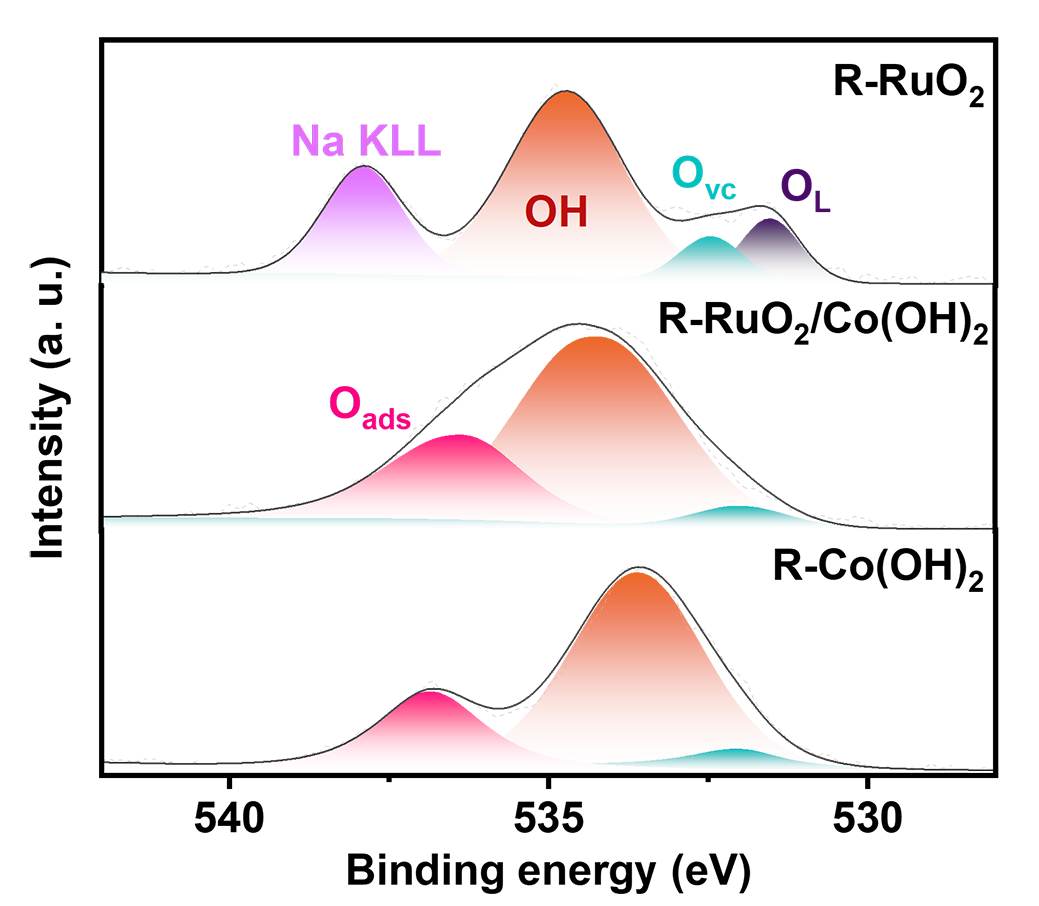


Figure S18. High-resolution XPS spectra of O 1*s* in R-RuO_2_, R-RuO_2_/Co(OH)_2_ and R-Co(OH)_2_.


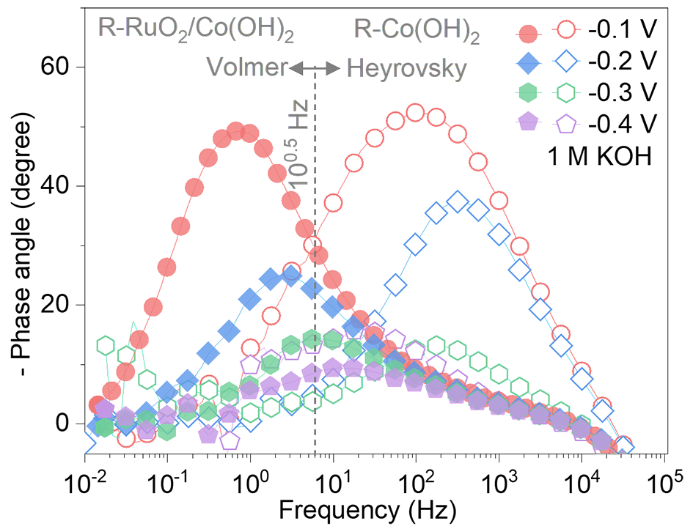


Figure S19. Bode plots at various potentials over R-Co(OH)_2_ and R-RuO_2_/Co(OH)_2_, with the electrolyte being 1 M KOH.

HER processes involve three steps: the Volmer step (H_2_O + M + e^−^ → M-*H + OH^−^), the Heyrovsky step (H_2_O + M-*H + e^−^ → M + H_2_ + OH^−^), and the Tafel step ( 2M-*H→ 2M + H_2_)^[20]^. Unlike the Tafel step, the Volmer and Heyrovsky reactions involve the electron transfer, thus can be reflected at the low (≤10^0.5^ Hz) and middle (10^0.5^-10^2.5^ Hz) frequency ranges of the Bode plots (Figure S19).


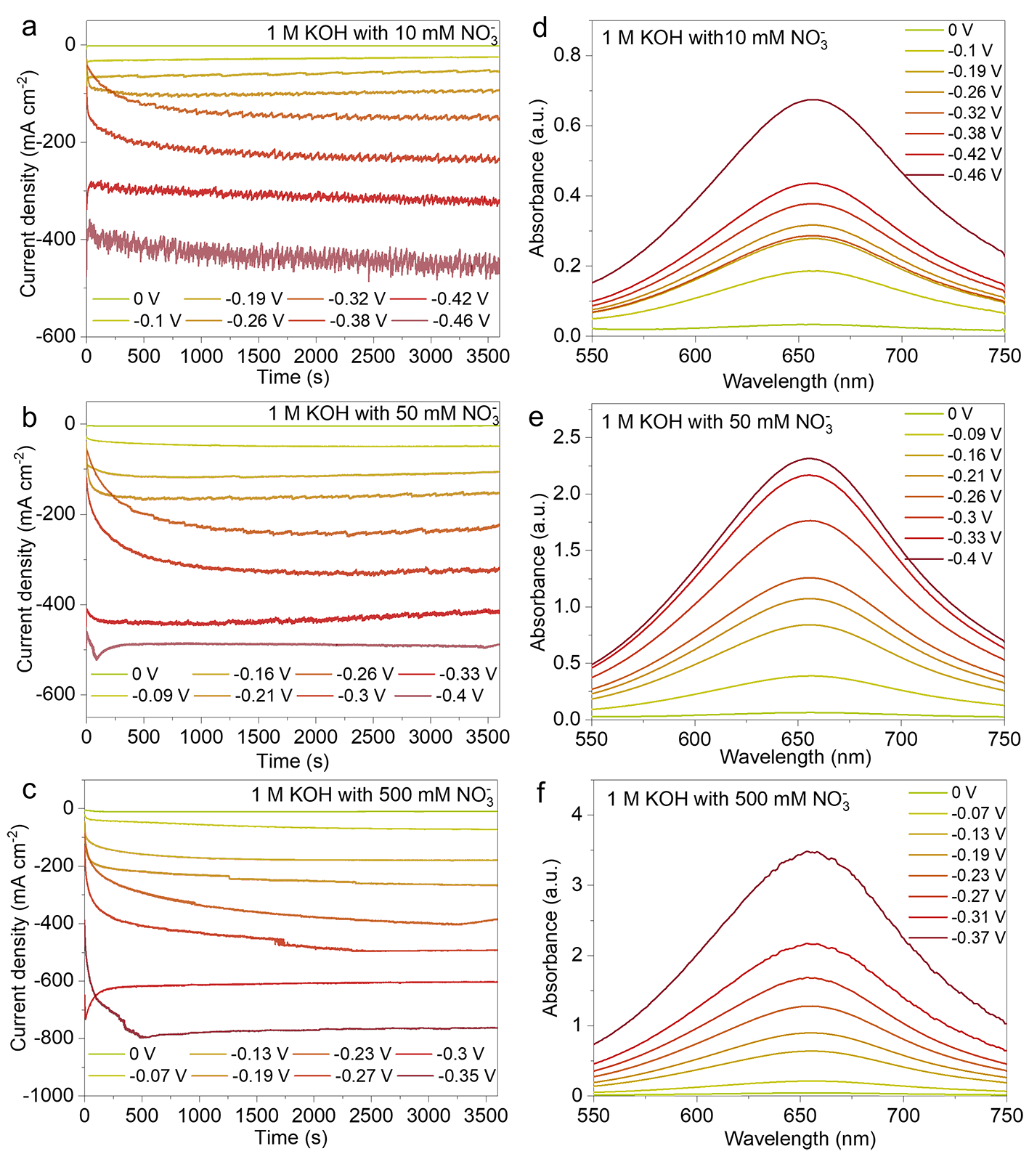


Figure S20. (a-c) The *j*-t curves at various potentials over R-RuO_2_/Co(OH)_2_ in 1 M KOH with various concentrations of NO_3_^−^. (d-f) The corresponding absorbance spectra.


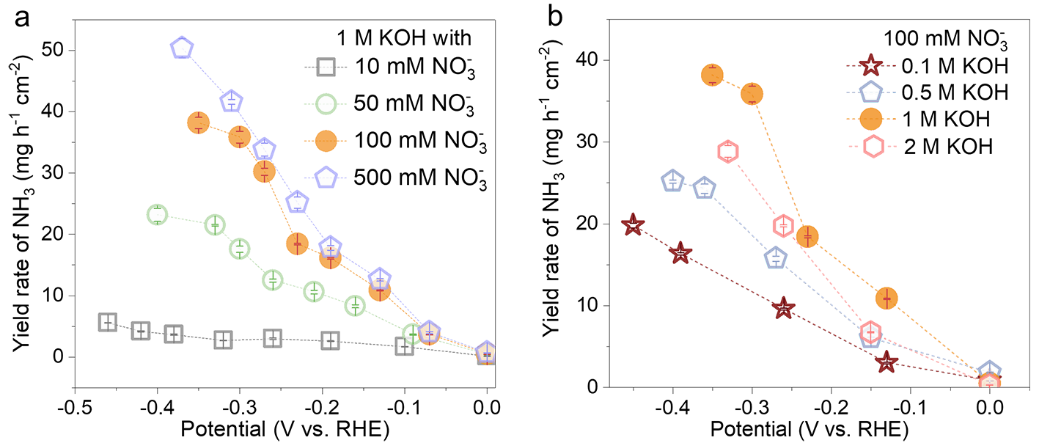


Figure S21. Potential-dependent NH_3_ yield rates over R-RuO_2_/Co(OH)_2_ in (a) 1 M KOH with varying concentrations of nitrate, and (b) 100 mM NO_3_^−^ with varying concentrations of KOH. Error bars represent the standard deviation from three independent measurements.


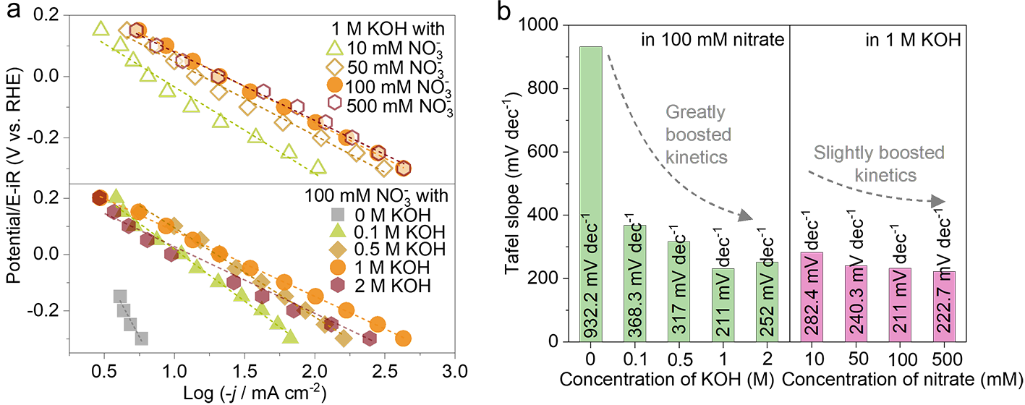


Figure S22. (a, b) Tafel slopes of R-RuO_2_/Co(OH)_2_ during eNO_3_RR.


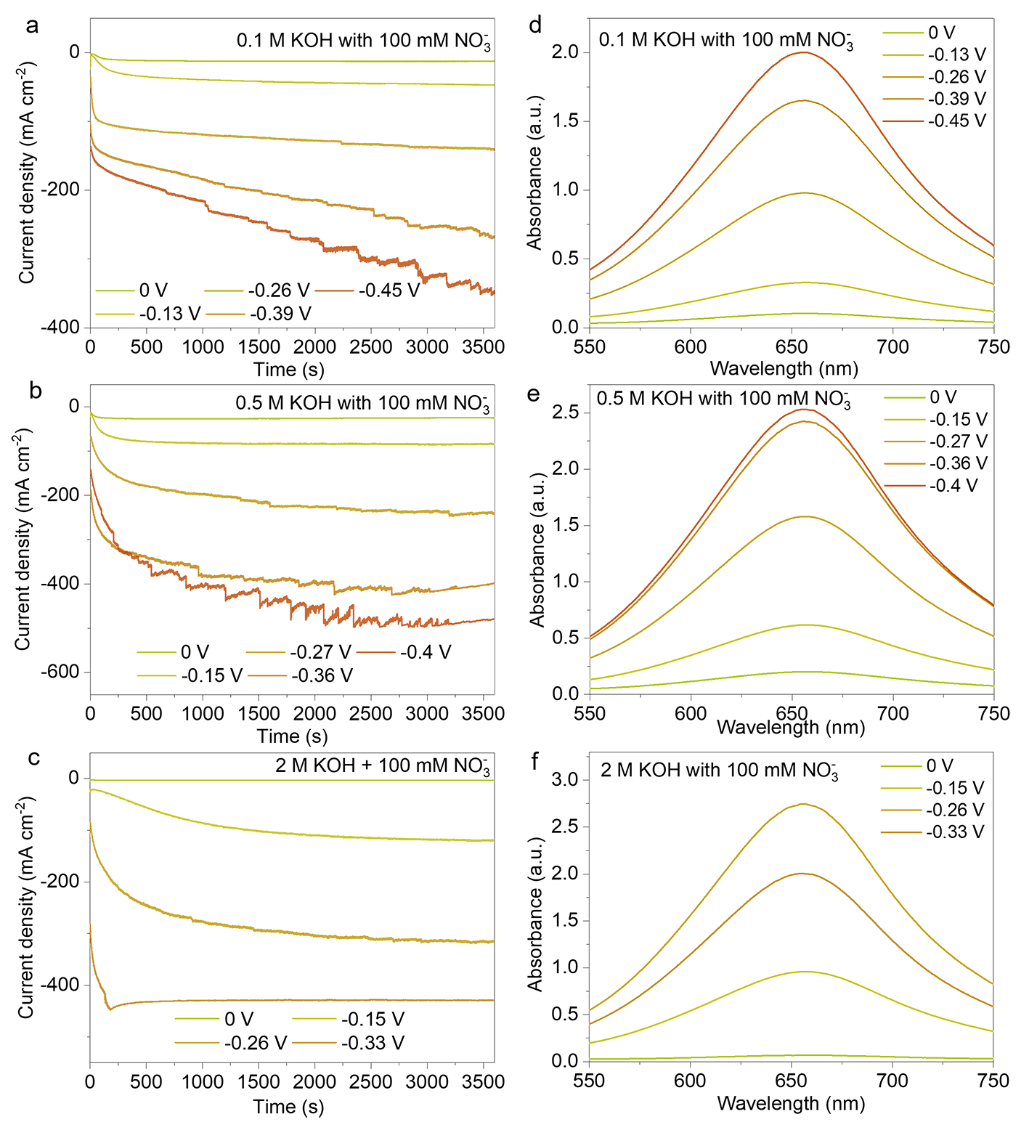


Figure S23. (a-c) The *j*-t curves at different applied potentials over R-RuO_2_/Co(OH)_2_ in 100 mM NO_3_^−^ with various concentrations of KOH. (d-f) The corresponding absorbance spectra.


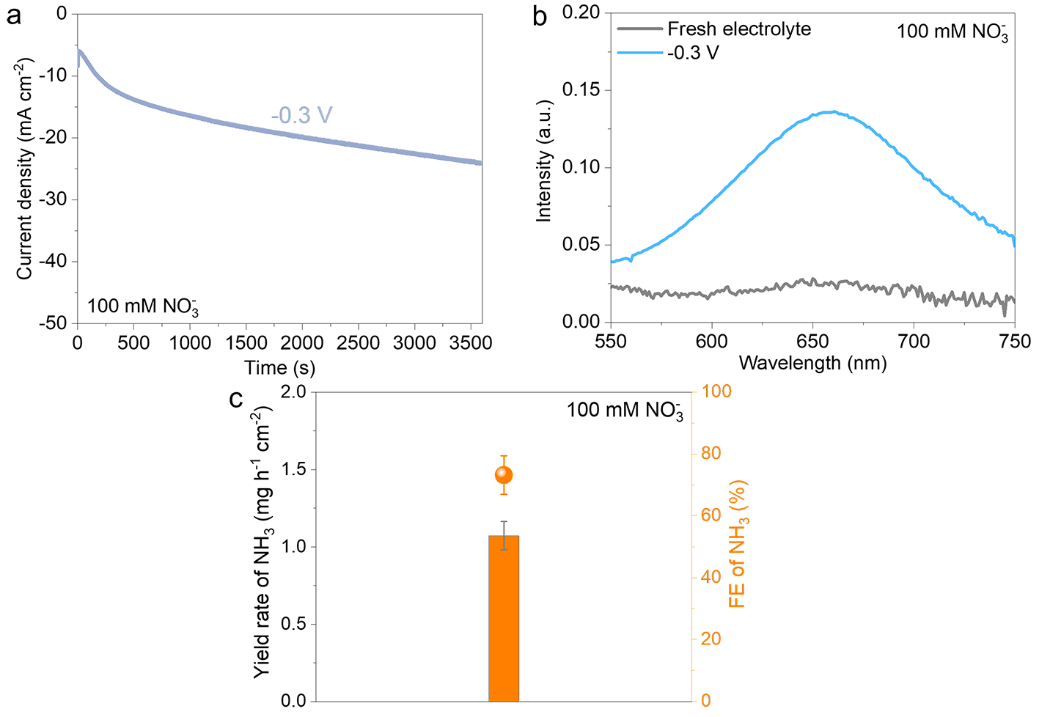


Figure S24. (a) The *j*-t curve of R-RuO_2_/Co(OH)_2_ in 100 mM NO_3_^−^ at −0.3 V. (b) The absorbance spectra of 100 mM NO_3_^−^ before and after catalysis at −0.3 V. (c) The corresponding yield rate and FE of NH_3_.


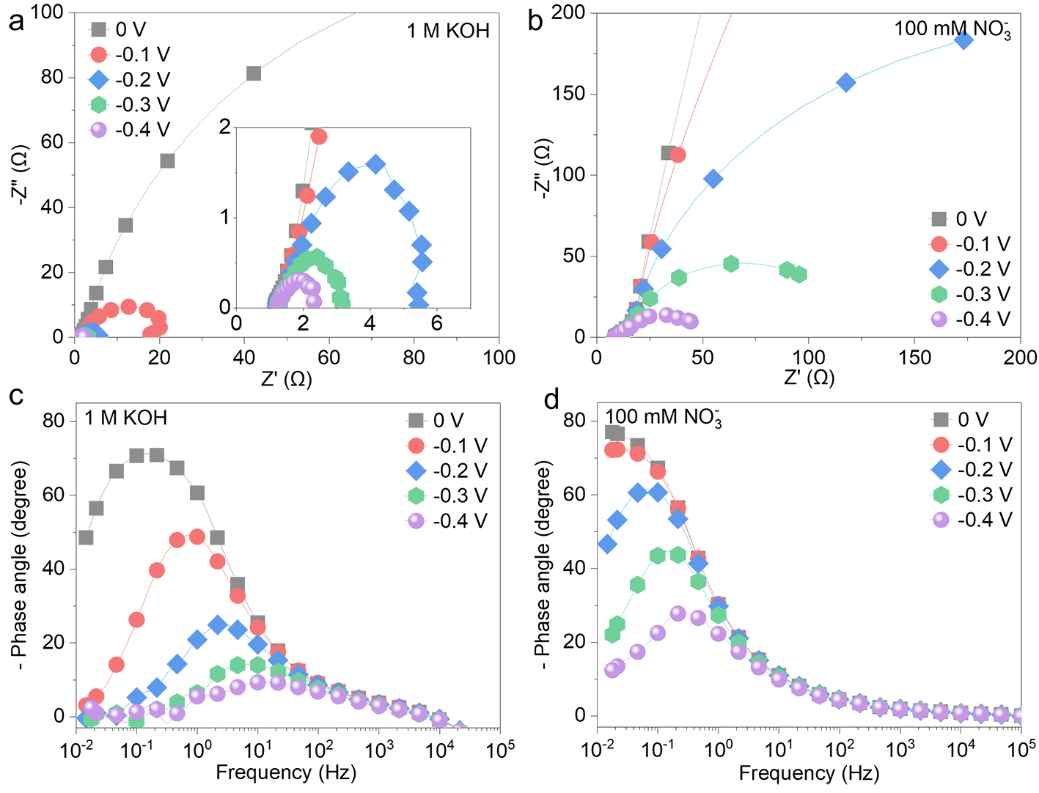


Figure S25. The potential-dependent EIS spectra of R-RuO_2_/Co(OH)_2_ in (a) 1 M KOH and (b) 100 mM NO_3_^−^. Bode plots of R-RuO_2_/Co(OH)_2_ in (c) 1 M KOH and (d) 100 mM NO_3_^−^, respectively.


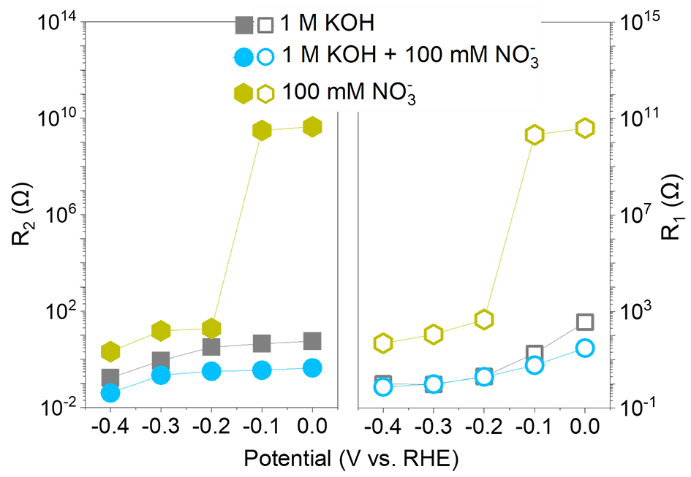


Figure S26. The fitted *R*_1_ and *R*_2_ values in various electrolytes from the *in*-*situ* EIS data.


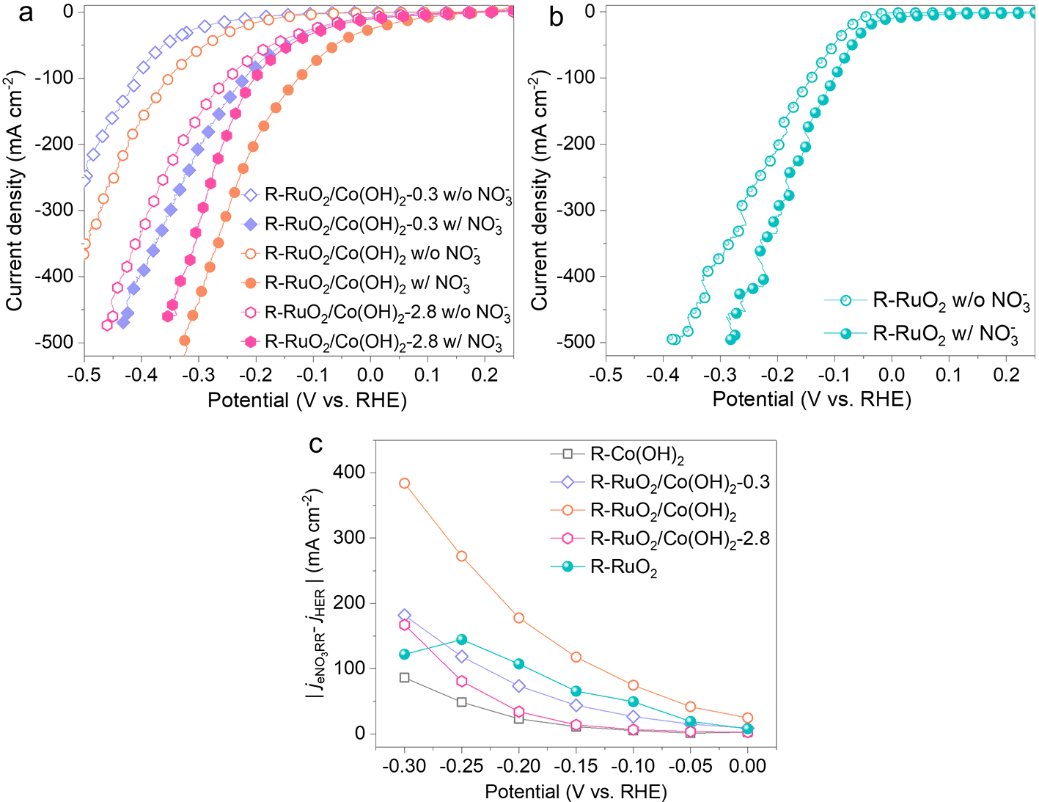


Figure S27. LSV curves of (a) various reconstructed RuO_2_/Co(OH)_2_ catalysts, and (b) the reconstructed RuO_2_ in 1 M KOH with (w/) and without (w/o) 100 mM NO_3_^−^. (c) The current density difference of eNO_3_RR and HER at various potentials.


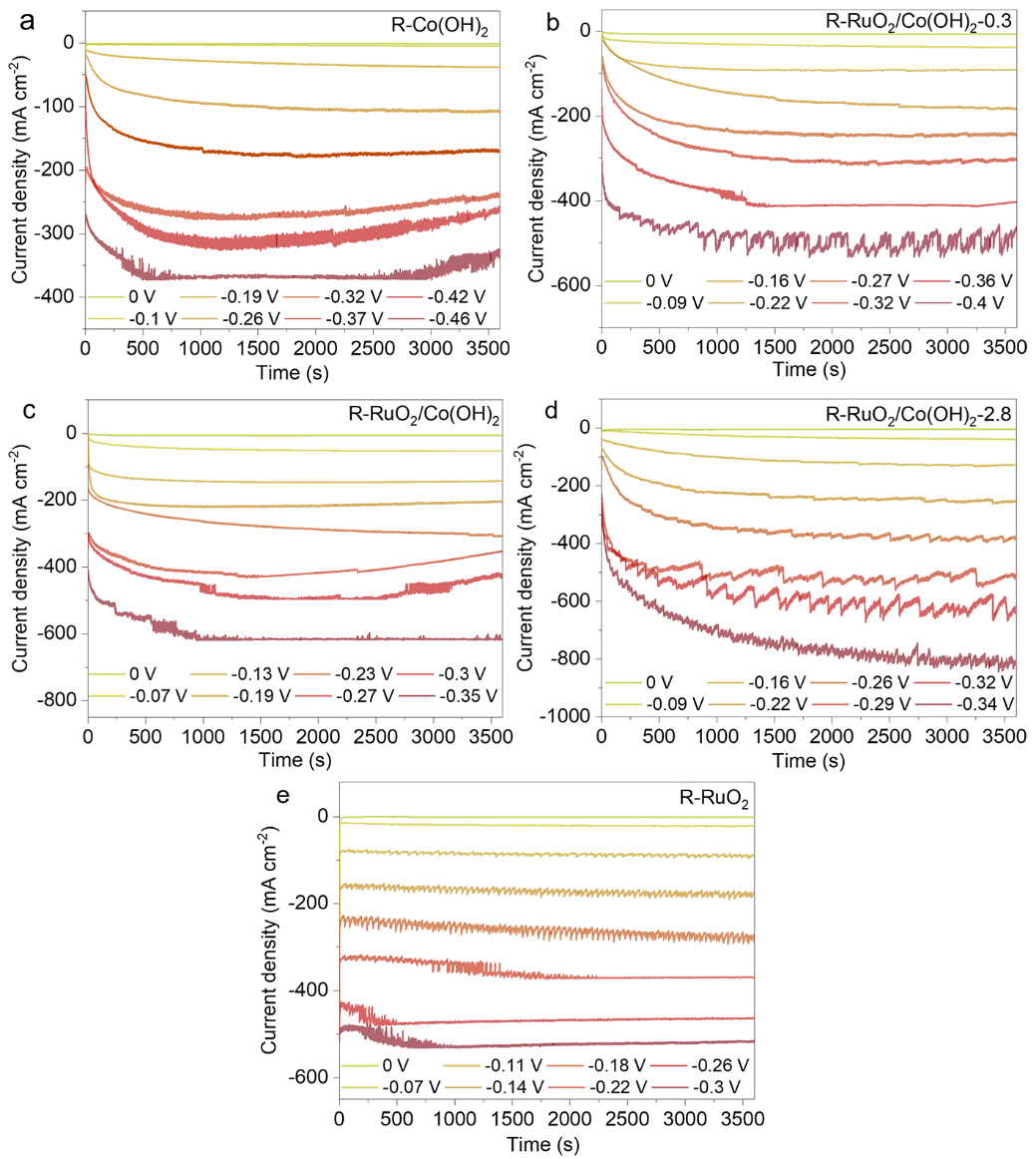


Figure S28. Current density-time (*j*-t) curves at various potentials in 1 M KOH with 100 mM NO_3_^−^ over (a) R-Co(OH)_2_, (b) R-RuO_2_/Co(OH)_2_-0.3, (c) R-RuO_2_/Co(OH)_2_, (d) R-RuO_2_/Co(OH)_2_-2.8, and (e) R-RuO_2_.


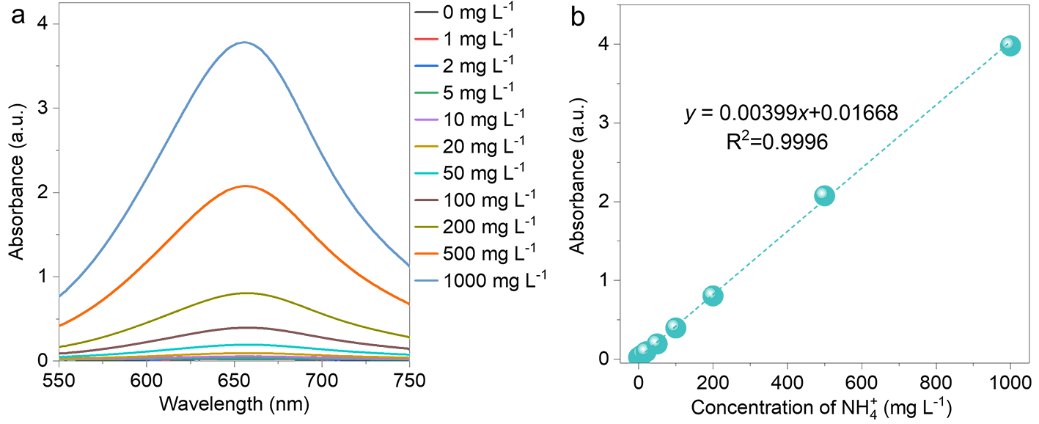


Figure S29. (a) Absorbance curves of standard NH_4_Cl, and (b) the absorbance intensity-concentration correlation at 655 nm of NH_4_^+^.


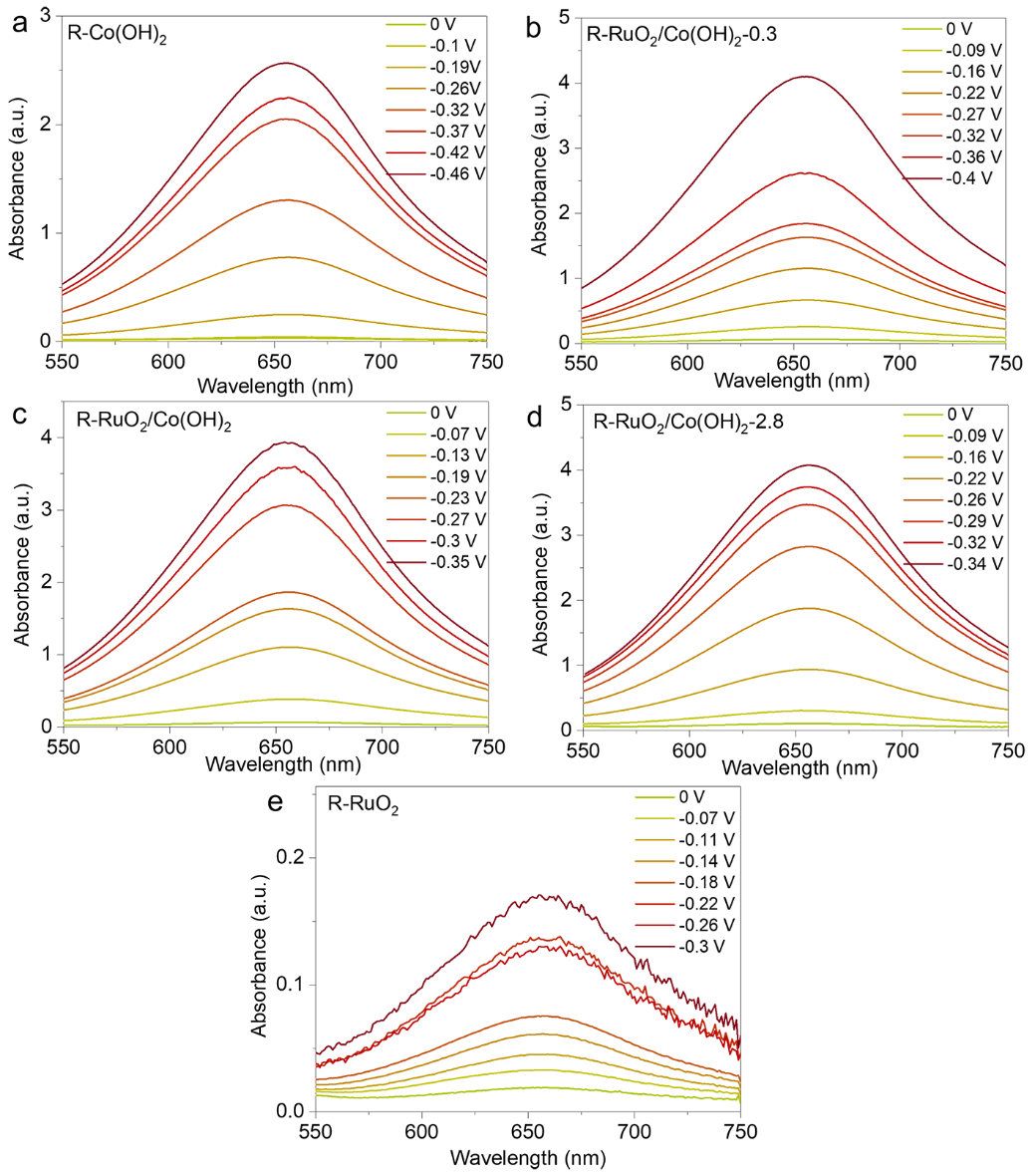


Figure S30. The measured absorbance curves of ammonia at various potentials over (a) R-Co(OH)_2_, (b) R-RuO_2_/Co(OH)_2_-0.3, (c) R-RuO_2_/Co(OH)_2_, (d) R-RuO_2_/Co(OH)_2_-2.8, and (e) R-RuO_2_.


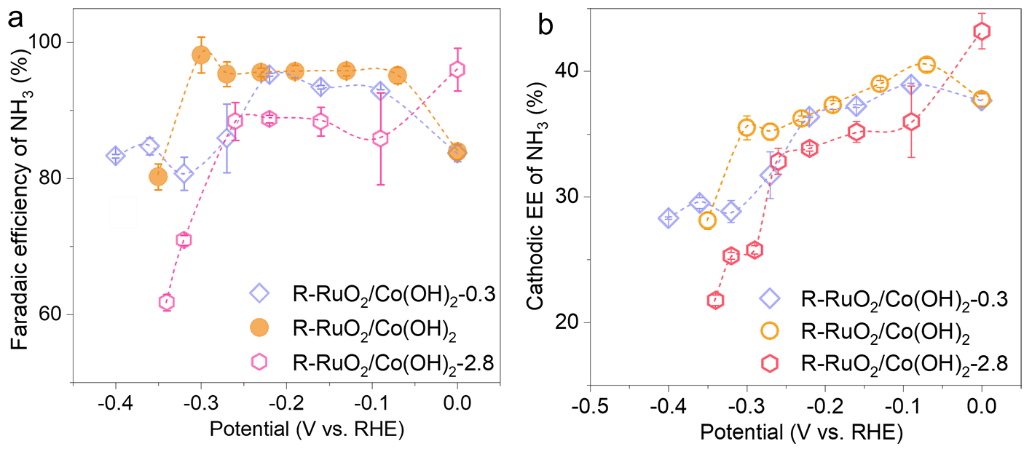


Figure S31. (a) The FEs, and (b) EEs of NH_3_ at different potentials. Error bars represent the standard deviation from three independent measurements.


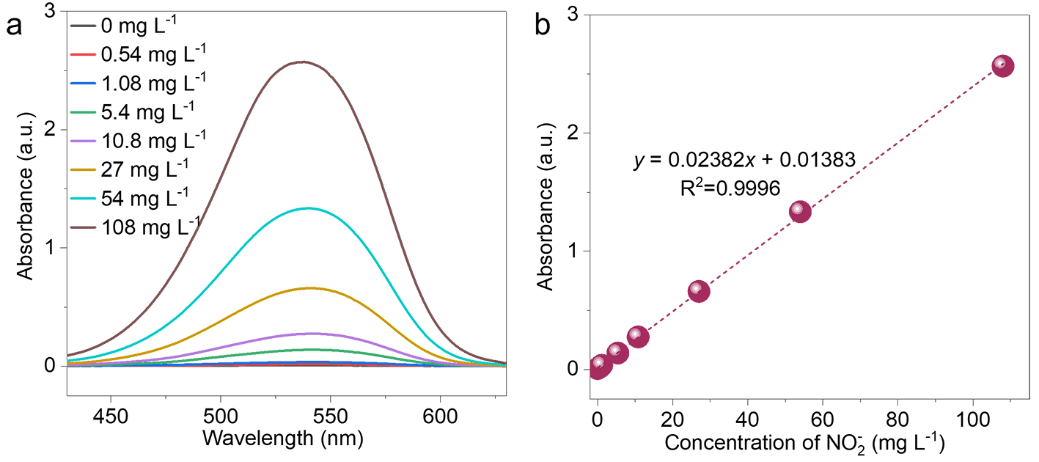


Figure S32. (a) Absorbance curves of standard NO_2_^−^, and (b) the absorbance intensity-NO_2_^−^ concentration correlation.


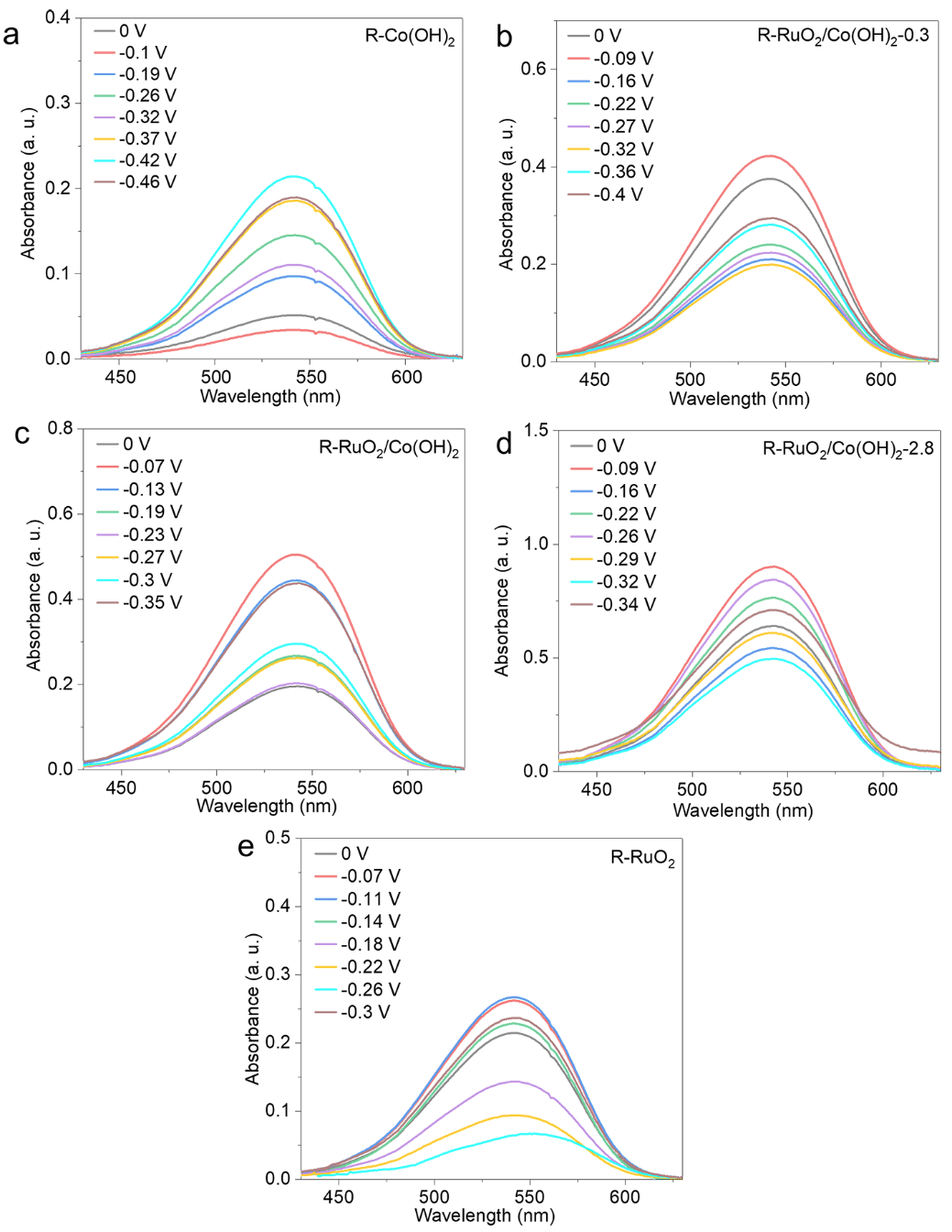


Figure S33. The measured absorbance curves of NO_2_^−^ at various potentials over (a) R-Co(OH)_2_, (b) R-RuO_2_/Co(OH)_2_-0.3, (c) R-RuO_2_/Co(OH)_2_, (d) R-RuO_2_/Co(OH)_2_-2.8, and (e) R-RuO_2_.


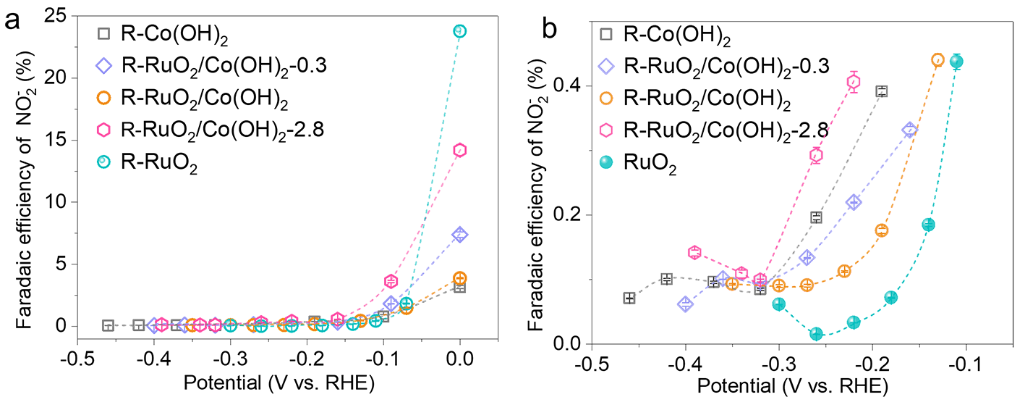


Figure S34. (a) The FEs, and (b) EEs of NO_2_^−^ at different potentials.


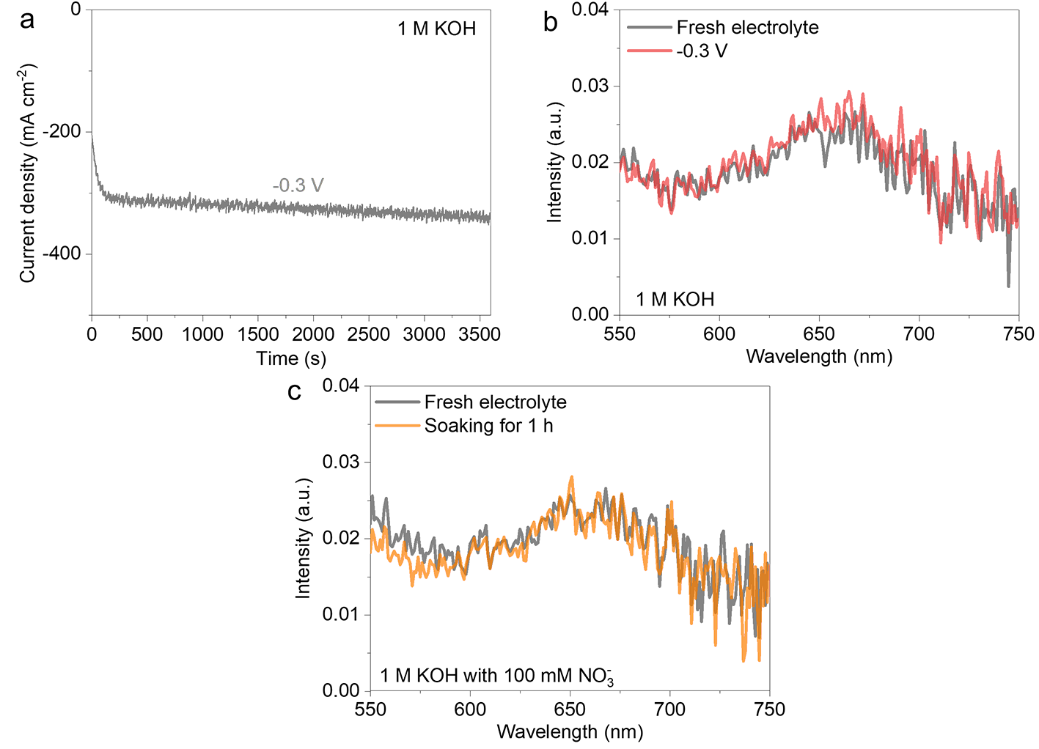


Figure S35. (a) The *j*-t curve of R-RuO_2_/Co(OH)_2_ in 1 M KOH at −0.3 V. (b) Absorbance curves of 1 M KOH before and after catalysis over R-RuO_2_/Co(OH)_2_ at −0.3 V for 1 hour. (c) Absorbance curves of 1 M KOH with 100 mM NO_3_^−^ before and after soaking with the R-RuO_2_/Co(OH)_2_ catalyst for 1 hour.


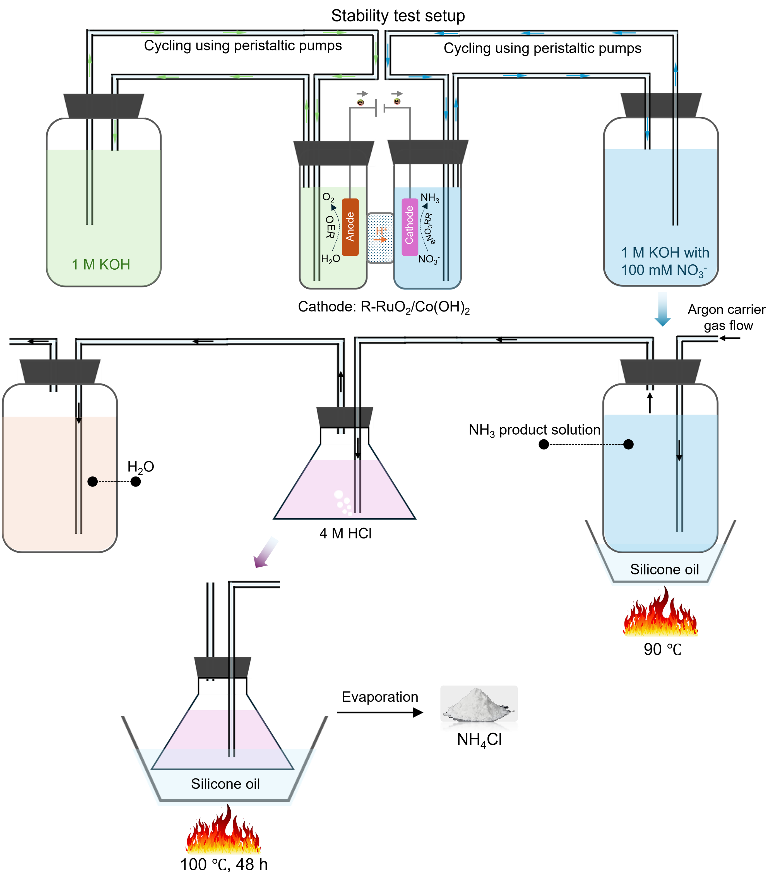


Figure S36. Schematic illustration of the synthesis and extraction of produced ammonia catalyzed by the R-RuO_2_/Co(OH)_2_ catalyst *via* eNO_3_RR, where the product is the NH_4_Cl(s) powder.


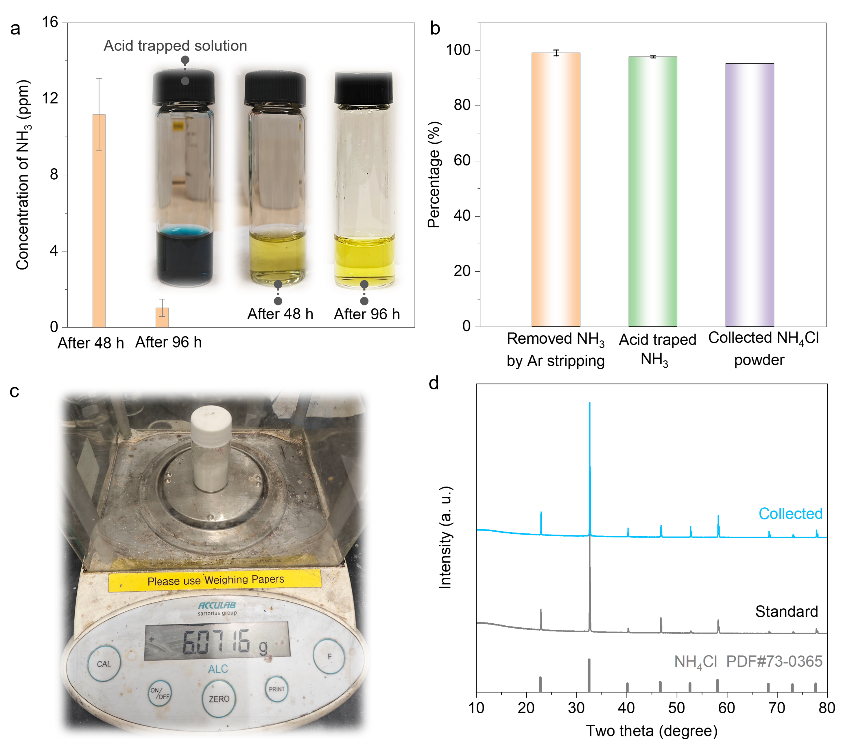


Figure S37. (a) the residual concentration of ammonia in the reacted electrolyte after the extraction from argon stripping. (b) The percentage of NH_3_ during different operations. (c) a snapshot of the collected NH_4_Cl powder. (d) XRD patterns of the standard and collected NH_4_Cl. Error bars in Figure a and b represent the standard deviation from three independent measurements.


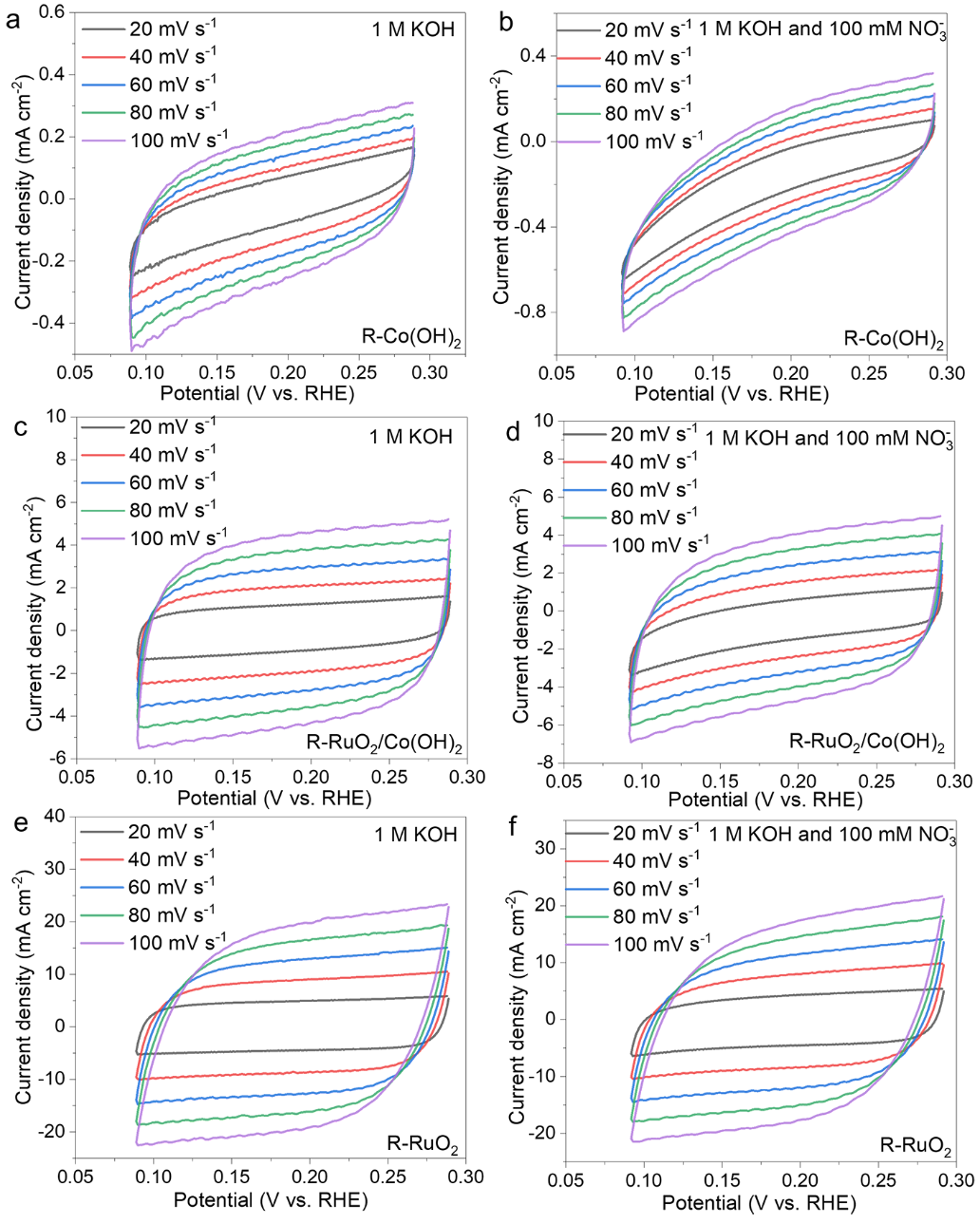


Figure S38. CV curves of (a, b) R-Co(OH)_2_, (c, d) R-RuO_2_/Co(OH)_2_, (e, f) R-RuO_2_ with various scan rates.


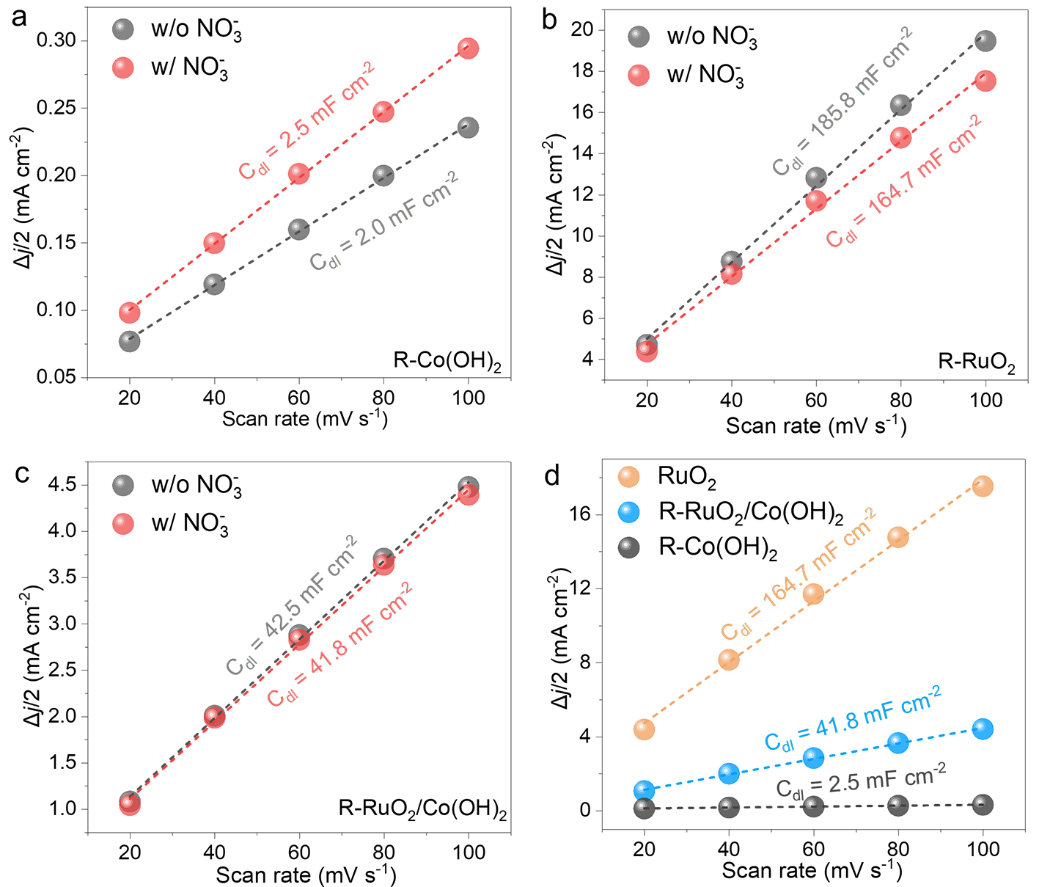


Figure S39. The calculated *C*_dl_ values of (a) R-Co(OH)_2_, (b) R-RuO_2_, (c) R-RuO_2_/Co(OH)_2_ in 1 M KOH with and without 100 mM NO_3_^−^. (d) *C*_dl_ values of various catalysts in 1 M KOH with 100 mM NO_3_^−^.


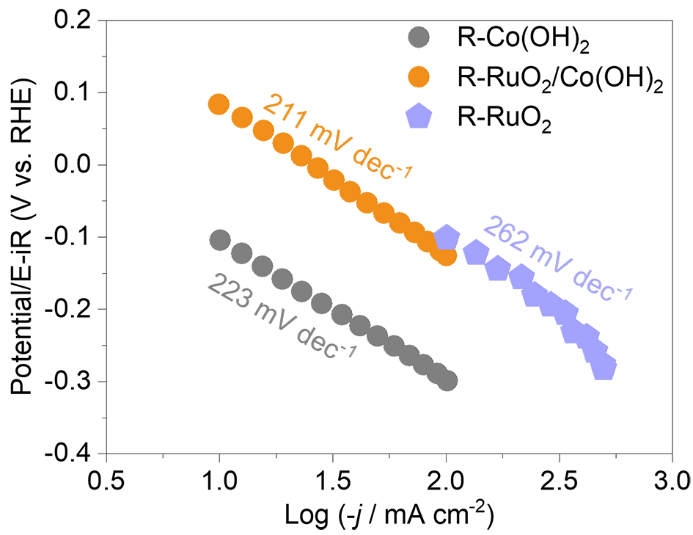


Figure S40. Tafel slope of various catalysts in 1 M KOH containing 100 mM NO_3_^−^.


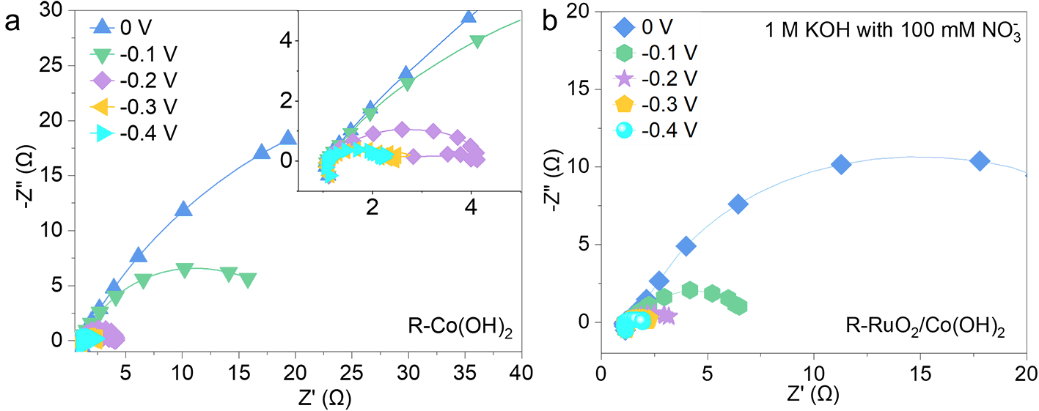


Figure S41. Potential-dependent EIS spectra of (a) R-Co(OH)_2_, and (c) R-RuO_2_/Co(OH)_2_ in 1 M KOH with 100 mM NO_3_^−^.


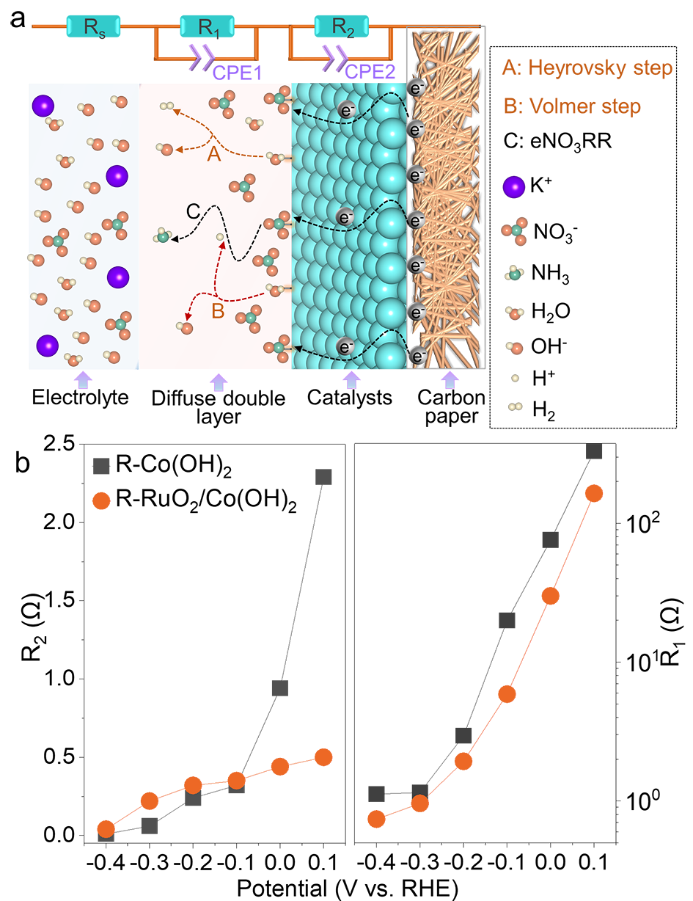


Figure S42. (a) The equivalent circuit model used in fitting the electrochemical impedance spectra (EIS) and schematic diagram of the proposed reactions. (b) The fitted *R*_1_ and *R*_2_ values for R-Co(OH)_2_ and R-RuO_2_/CO(OH)_2_ in 1 M KOH with 100 mM NO_3_^−^.


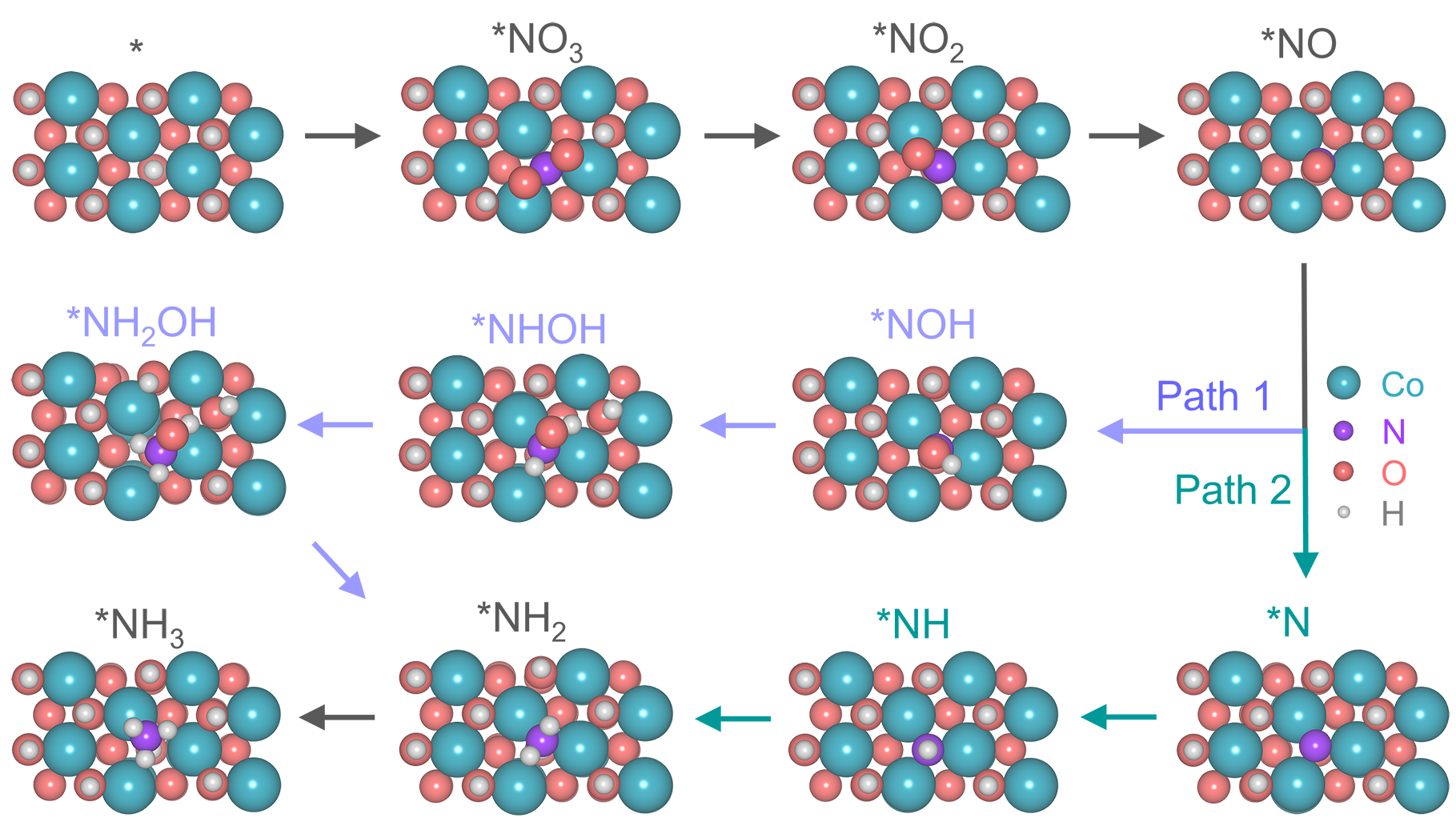


Figure S43. Structural diagrams in top view of Co(OH)_2_ with the interaction of various intermediates.


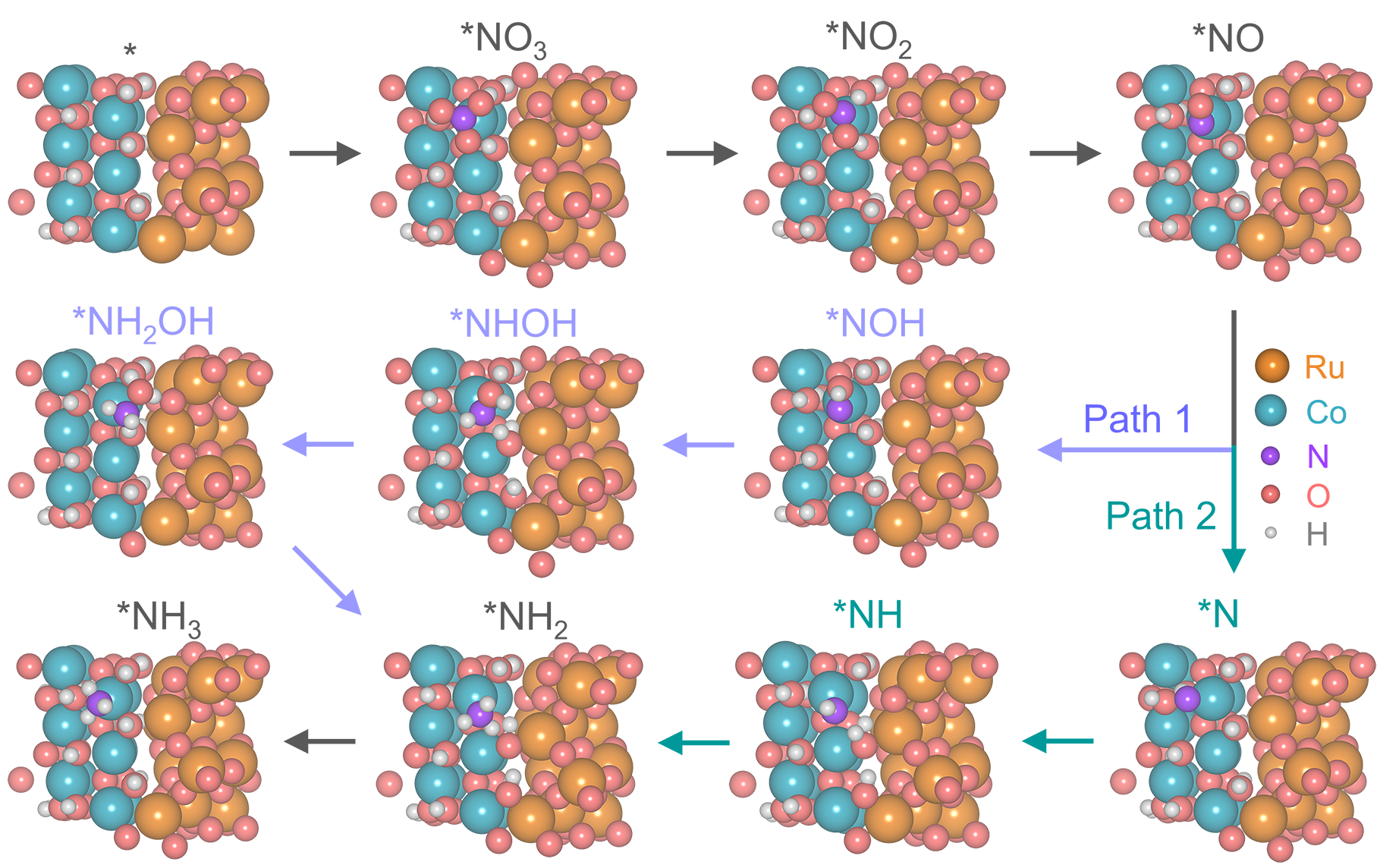


Figure S44. Structural diagrams in top view of RuO_2_/Co(OH)_2_ with the interaction of various intermediates.


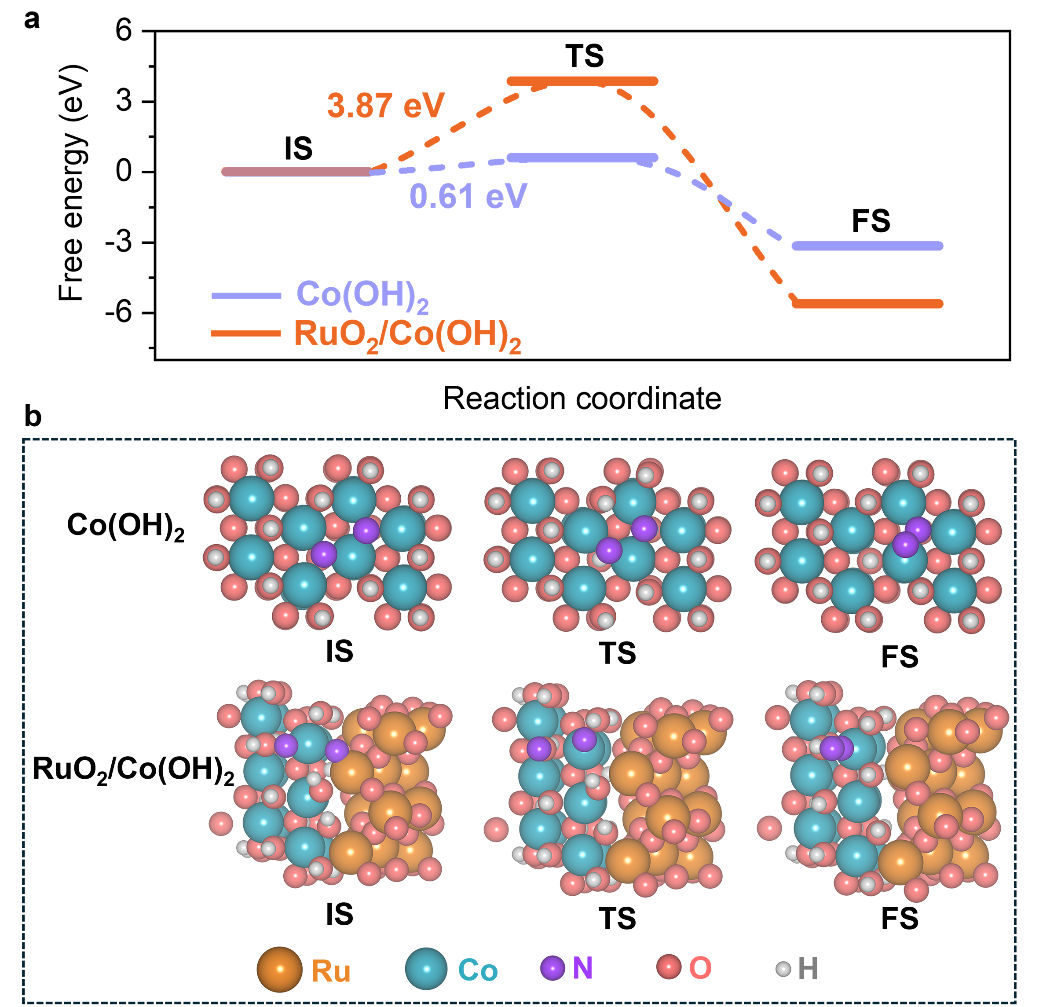


Figure S45. (a) Calculated kinetic barrier of N_2_ formation from *N–*N coupling on Co(OH)_2_ and RuO_2_/Co(OH)_2_, and (b) the corresponding structural diagrams for each step (IS: initial state; TS: transition state; FS: final state).


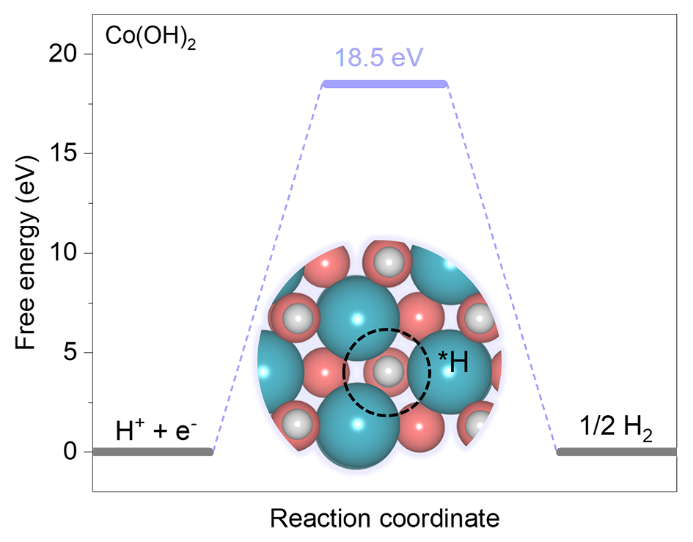


Figure S46. Calculated free energy of *H adsorption on Co(OH)_2_ surface.


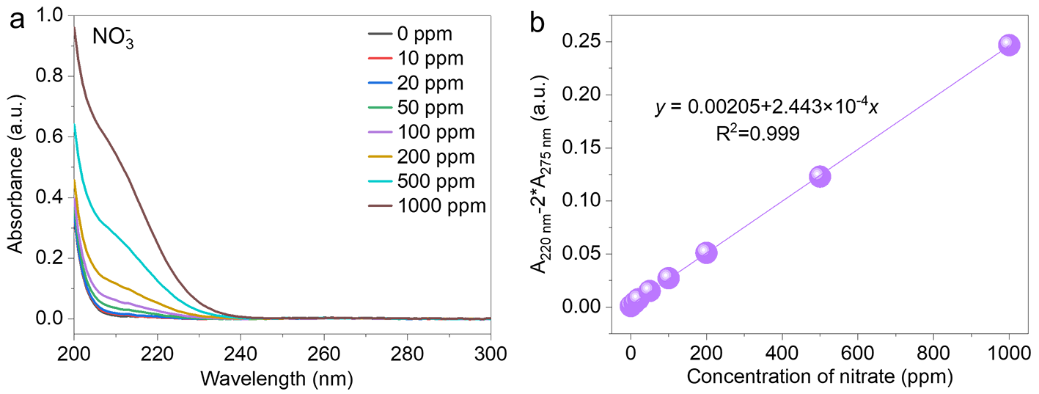


Figure S47. (a) Absorbance curves of standard NO_3_^−^, and (b) the absorbance intensity-concentration correlation of NO_3_^−^.


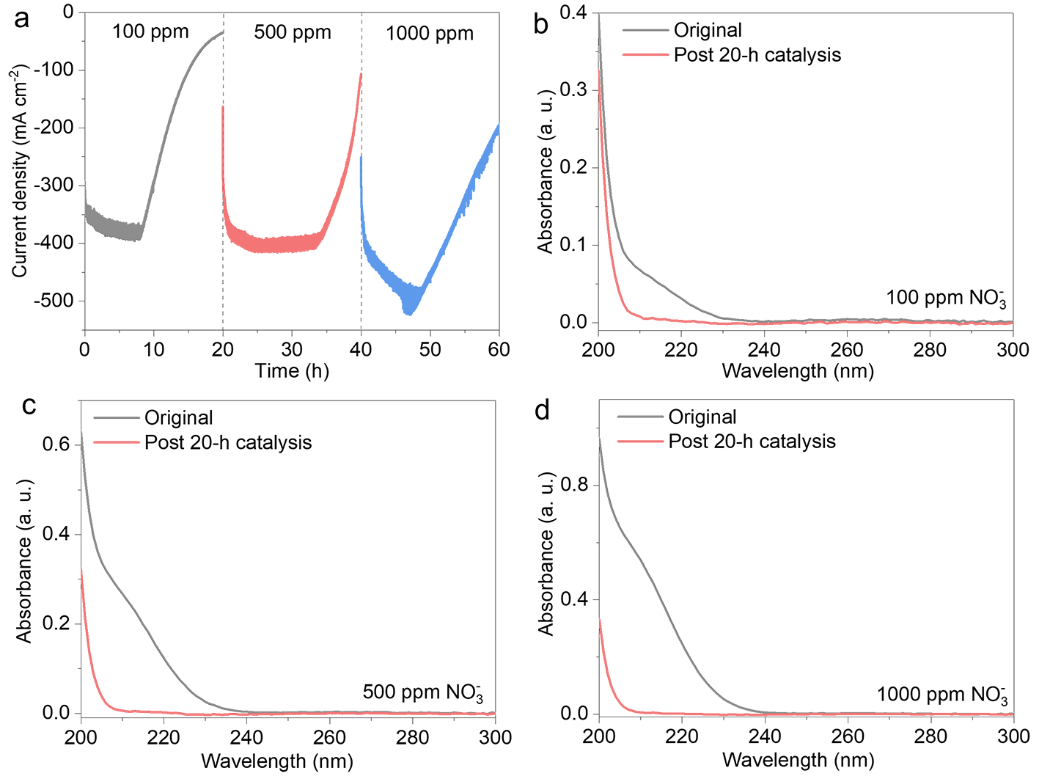


Figure S48. (a) The *j*-t curves of R-RuO_2_/Co(OH)_2_ at −0.3 V during eNO_3_RR with the electrolyte being 1 M KOH containing 100, 500, and 1000 ppm NO_3_^−^. (b-d) The absorbance spectra of NO_3_^−^ before and after catalysis at −0.3 V.


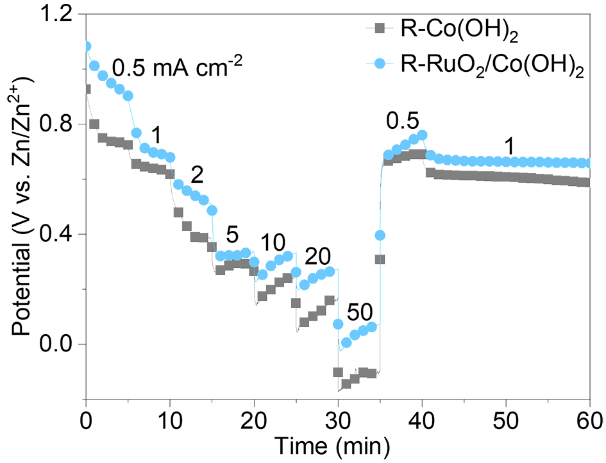


Figure S49. Rate performance of the Zn-NO_3_^−^ batteries with R-Co(OH)_2_, and R-RuO_2_/Co(OH)_2_ cathodes.


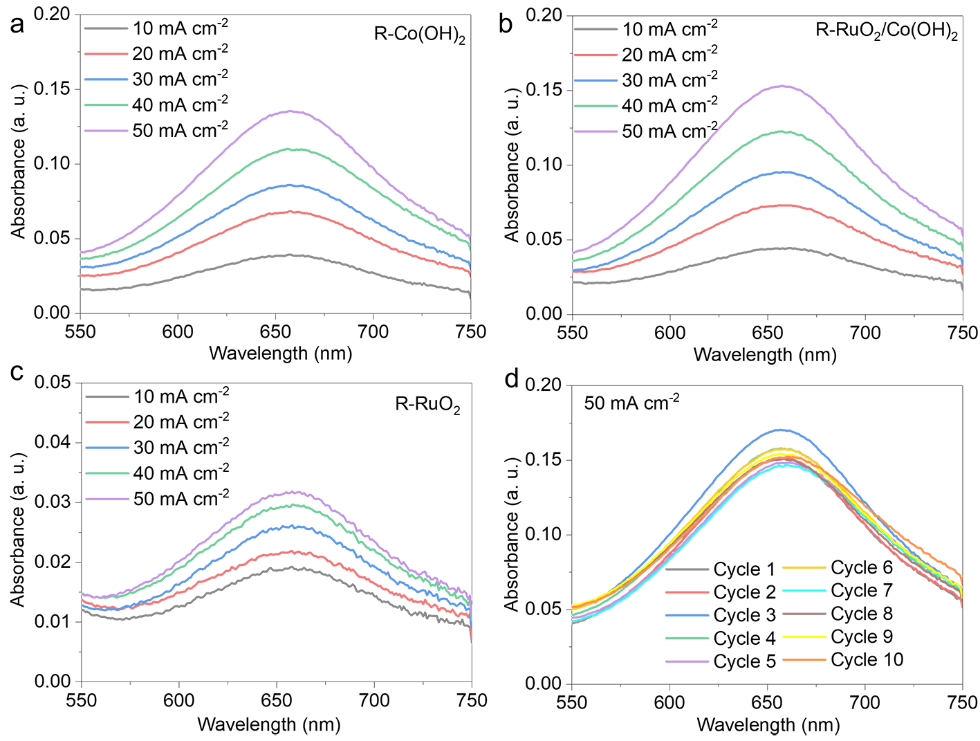


Figure S50. Absorbance spectra of ammonia for (a) R-Co(OH)_2_, (b) R-RuO_2_/Co(OH)_2_, and (c) R-RuO_2_ in Zn-NO_3_^−^ batteries after catalysis at various current densities. (d) Absorbance spectra of NH_3_ for ten continuous cycles at 50 mA cm^−2^ in a Zn-NO_3_^−^ battery with the RuO_2_/Co(OH)_2_ cathode.


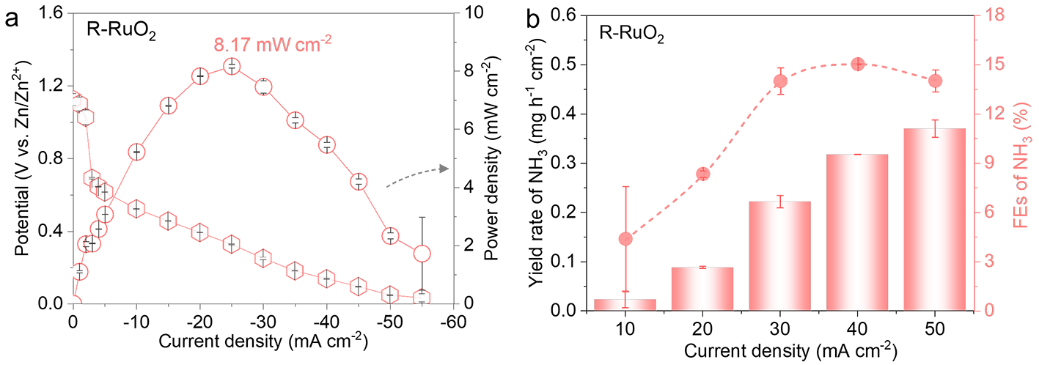


Figure S51. (a) The discharge curve and corresponding power density of the Zn-NO_3_^−^ battery with the R-RuO_2_ cathode. (b) NH_3_ yield rates and FEs at various current densities in a Zn-NO_3_^−^ battery with the R-RuO_2_ cathode. Error bars represent the standard deviation from three independent measurements.


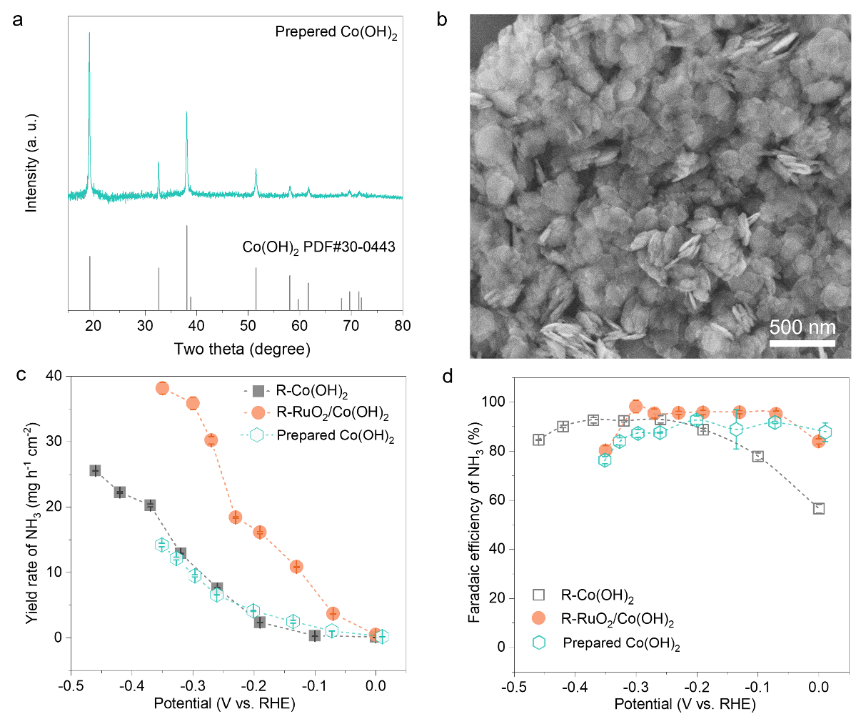


Figure S52. (a) XRD pattern and (b) SEM image of the directly prepared Co(OH)_2_ nanostructure. (c) yield rates and (d) FEs of NH_3_ over various catalysts. Error bars in Figure c and d represent the standard deviation from three independent measurements.


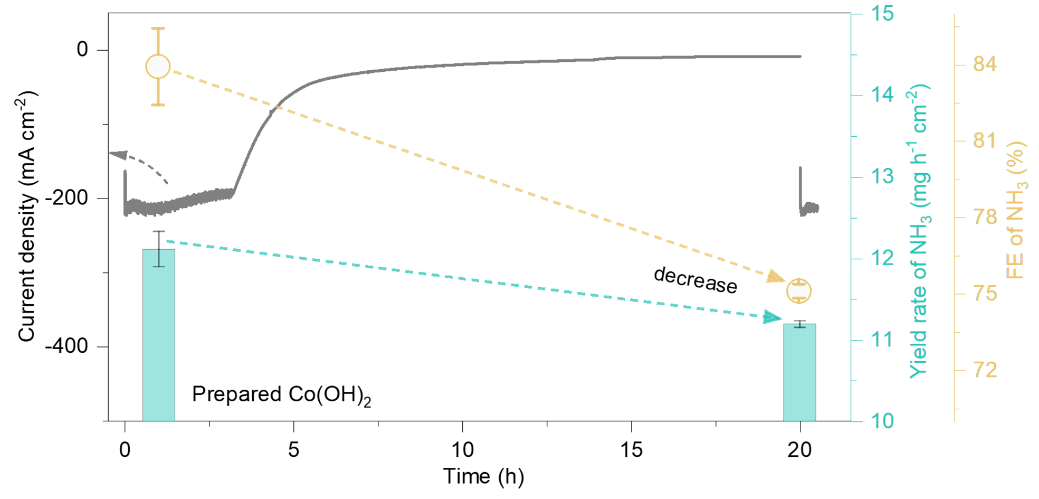


Figure S53. Stability test and the corresponding eNO_3_RR performance of the directly prepared Co(OH)_2_ catalyst. Error bars represent the standard deviation from three independent measurements.


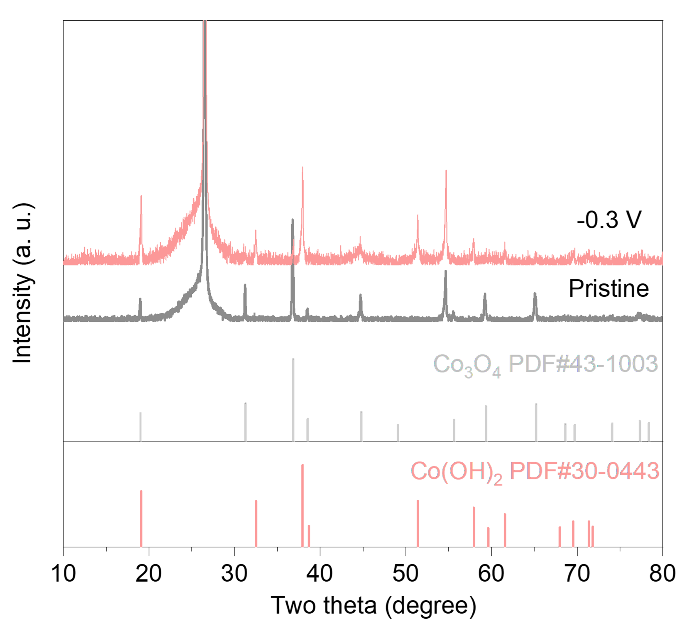


Figure S54. XRD patterns of the RuO_2_/Co_3_O_4_-1.1 sample before and after reconstruction at −0.3 V.


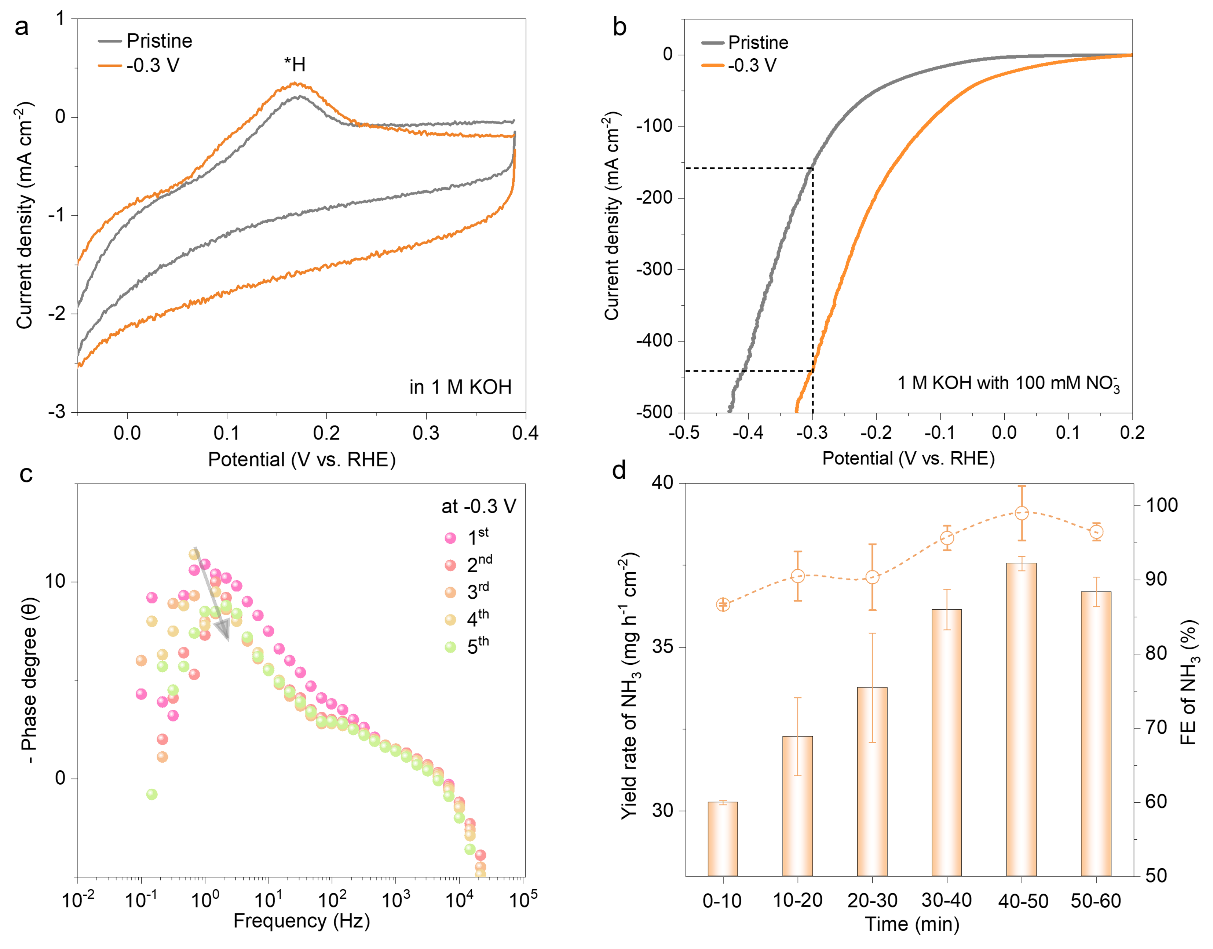


Figure S55. (a) CV curves of the pristine RuO_2_/Co_3_O_4_-1.1 and the catalyst after treated at −0.3 V, measured in 1 M KOH solution. (b) LSV curves of the pristine RuO_2_/Co_3_O_4_-1.1 and the catalyst after treated at −0.3 V. (c) Time-dependent Bode plots of the RuO_2_/Co_3_O_4_-1.1 at −0.3 V. (d) The NH_3_ yield rates and FEs at −0.3 V of the pristine RuO_2_/Co_3_O_4_-1.1 catalyst in 1 M KOH containing 100 mM NO_3_^−^, the electrolyte is refreshed for each 10 minutes. Error bars represent the standard deviation from three independent measurements.


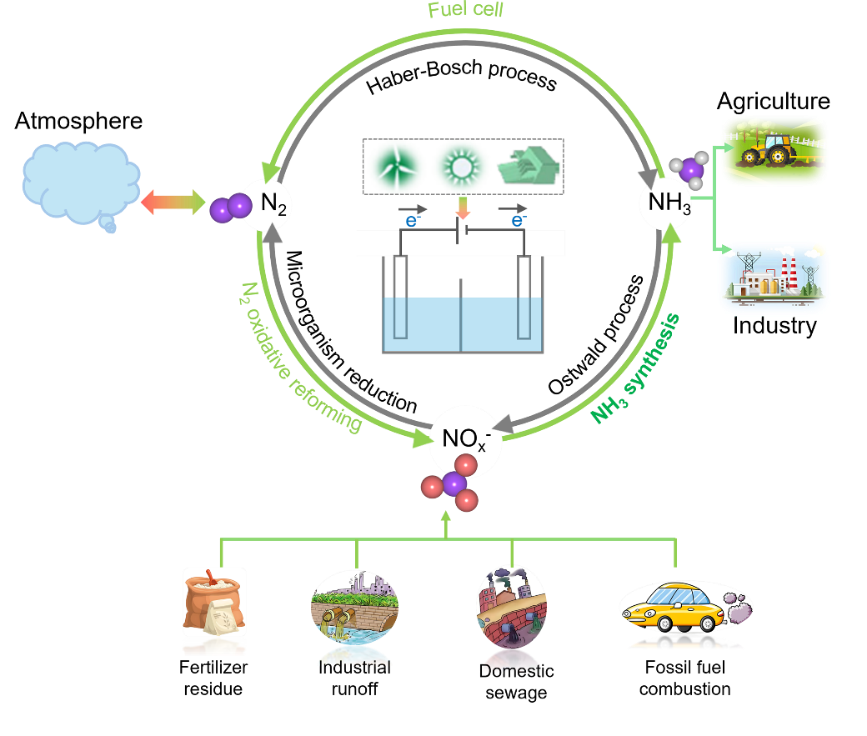


Figure S56. Nitrogen cycles. The conventional nitrogen cycle (grey arrow) based on heterogeneous catalysis and an electrocatalytic reverse artificial nitrogen cycle (green arrow) involving N_2_ oxidative reforming to NO_x_^−^ and its reverse electroreduction to ammonia using renewable energy. It shows the utilization of NH_3_ and the resources of NO_x_^−^ species.


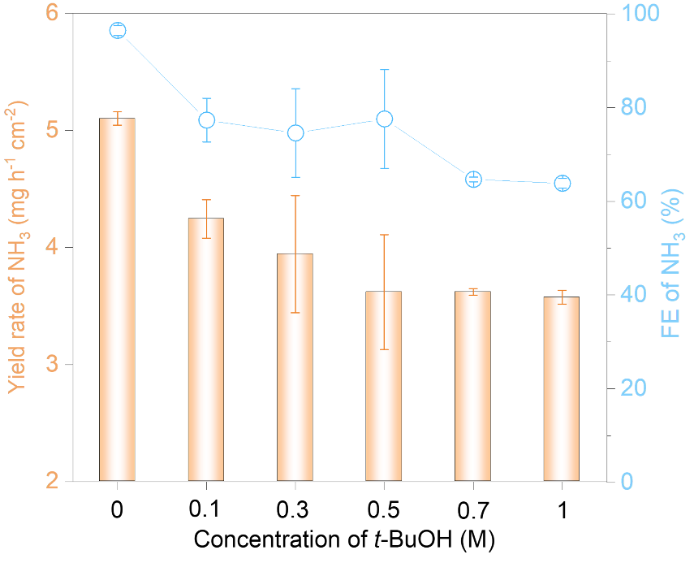


Figure S57. Yield rates and FEs of NH_3_ of the R-RuO_2_/Co(OH)_2_ catalyst in 1 M KOH containing 100 mM NO_3_^−^ with varying concentrations of *t*-BuOH additives. The current density was maintained at 100 mA cm^−2^. Error bars represent the standard deviation from three independent measurements.


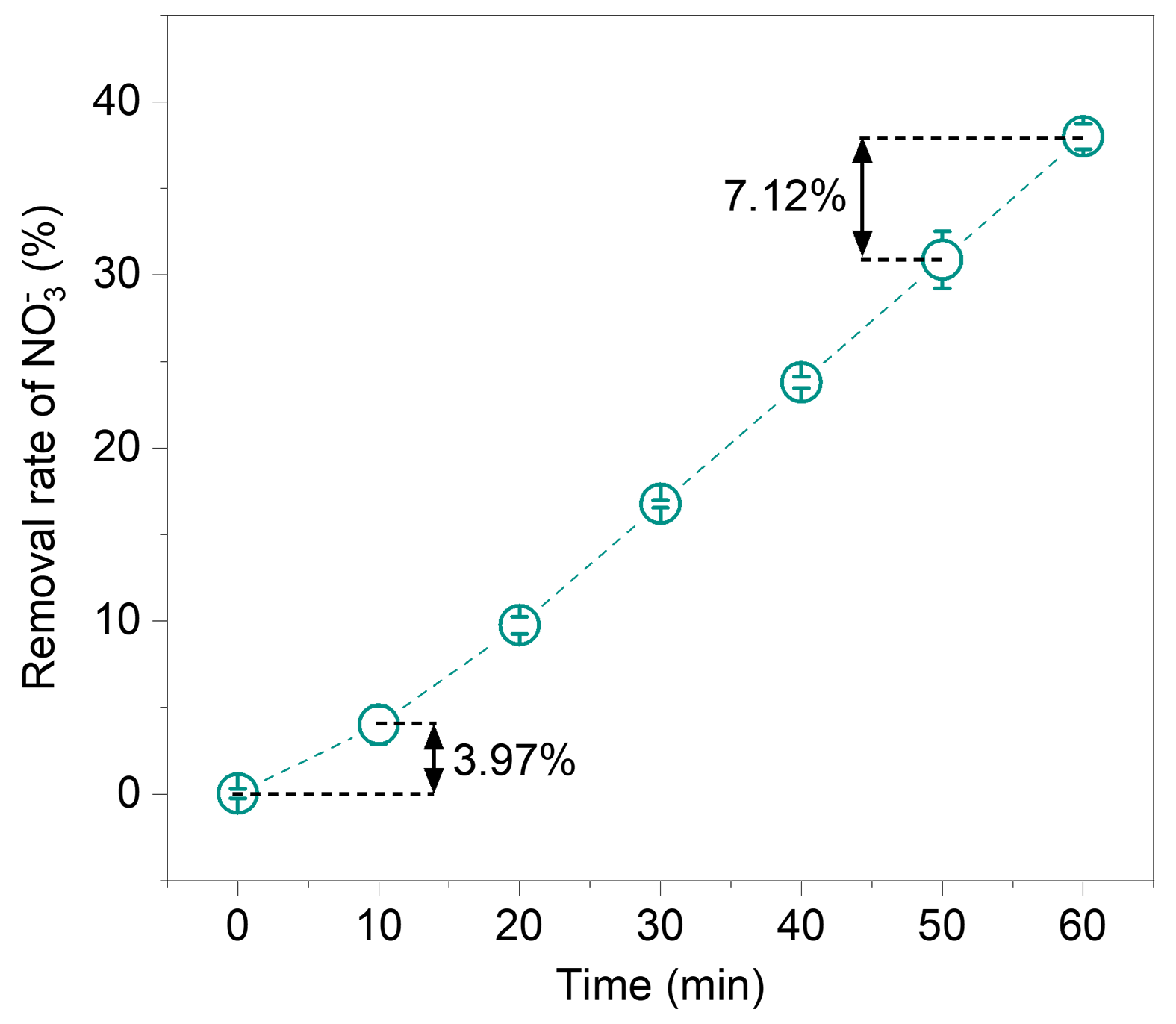


Figure S58. Time-dependent removal rate of nitrate for the Co_3_O_4_ precatalyst during the first hour at −0.3 V. The volume of the initial electrolytes was 30 mL, and 1 mL of the reacted electrolyte (1 M KOH containing 100 mM NO_3_^−^) was extracted for a 10-min catalysis interval. Error bars represent the standard deviation from three independent measurements.


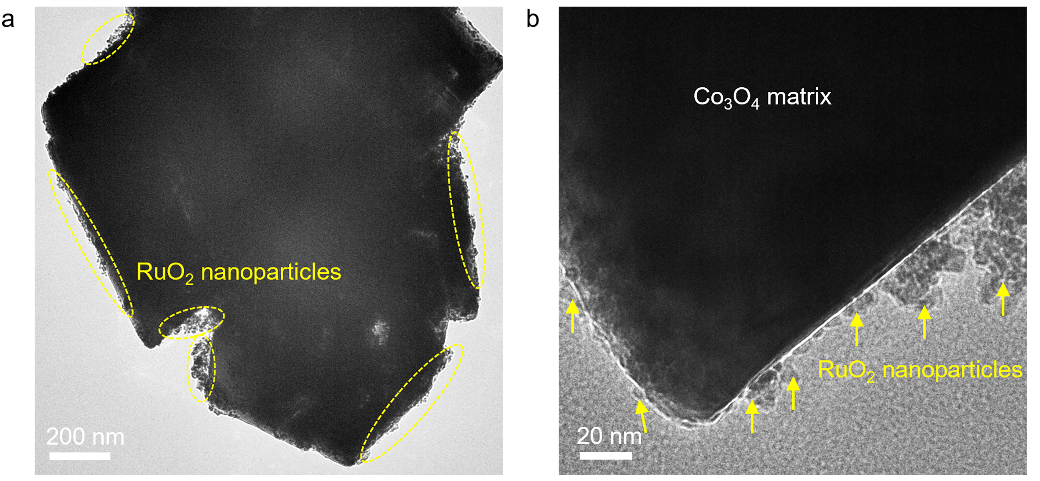


Figure S59. (a and b) TEM images of the RuO_2_/Co_3_O_4_-1.1 precatalyst.


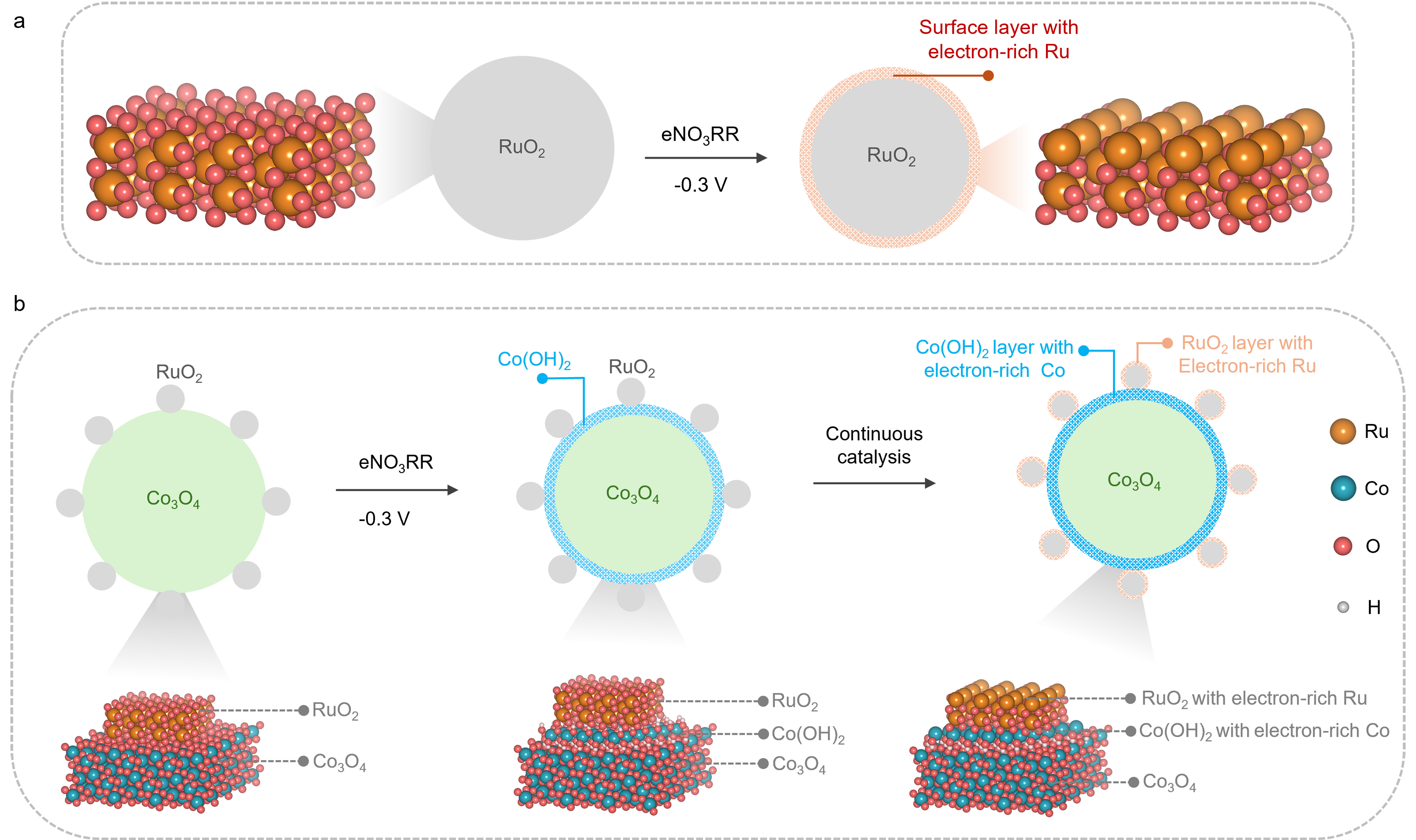


Figure S60. Diagrams of dynamic evolution for (a) RuO_2_ and (b) RuO_2_/Co_3_O_4_ precatalysts during eNO_3_RR.


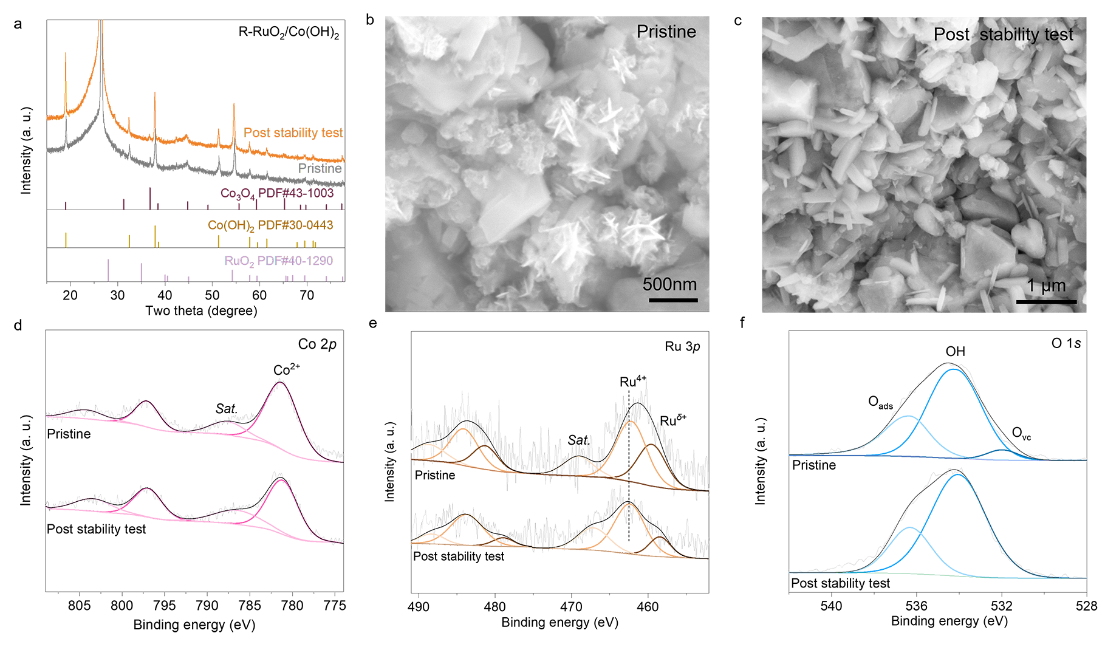


Figure S61. (a) XRD patterns of the R-RuO_2_/Co(OH)_2_ catalyst before and after stability test at −0.3 V for 100 hours. SEM images of the R-RuO_2_/Co(OH)_2_ catalyst (b) before and (c) after the stability test. High-resolution XPS spectra of (d) Co 2*p*, (e) Ru 3*p* and (f) O 1*s* for the R-RuO_2_/Co(OH)_2_ catalyst before and after the stability test.


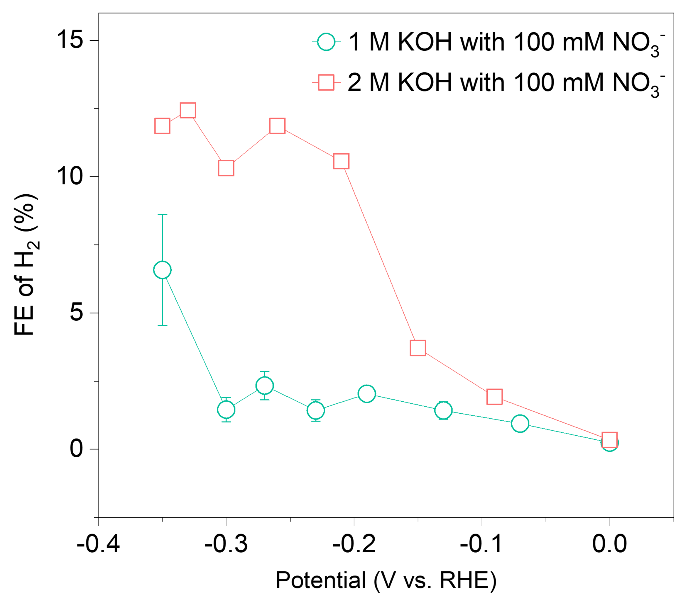


Figure S62. FEs of H_2_ in 100 mM NO_3_⁻ with 2 M and 1 M KOH over the R-RuO_2_/Co(OH)_2_ catalyst. Error bars represent the standard deviation from three independent measurements.


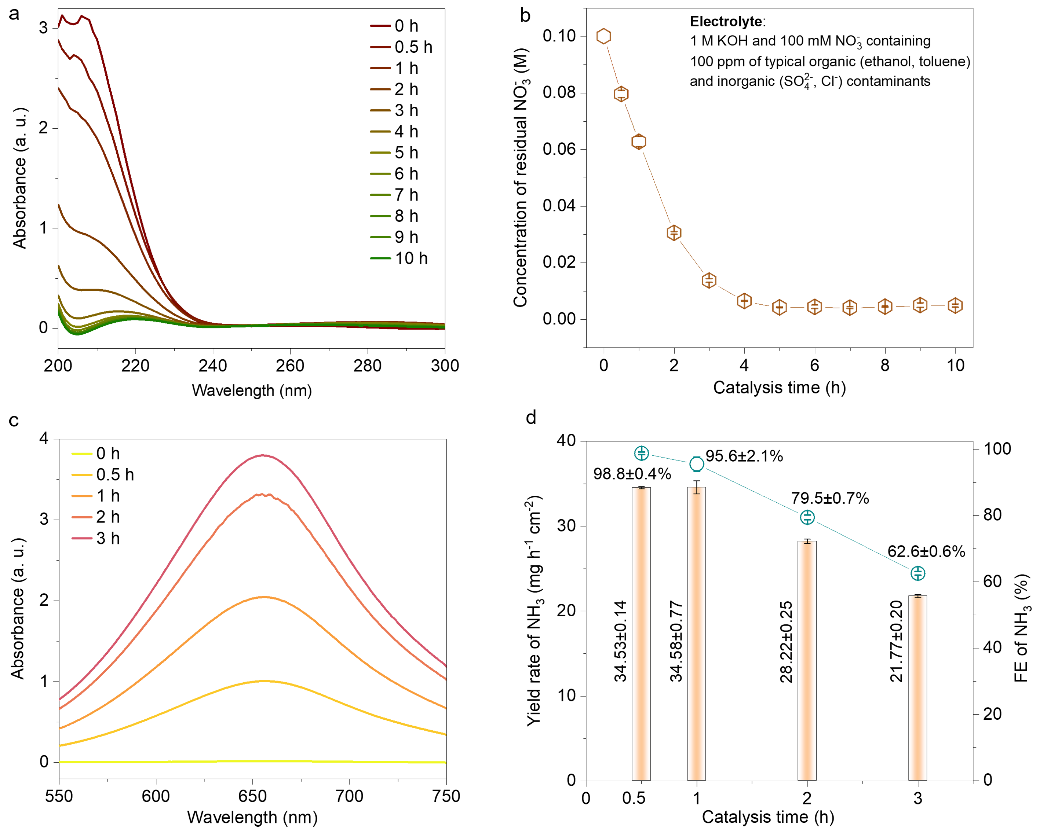


Figure S63. (a) The Uv-vis absorbance curves and (b) the concentration of residual NO_3_^−^ in the electrolyte after continuous catalysis at −0.3 V vs. RHE for different time periods (original electrolyte: 1 M KOH and 100 mM NO_3_^−^ with the mixture of 100 ppm SO_4_^2−^, 100 ppm Cl^−^, 100 ppm ethanol, and 100 ppm toluene; volume: 40 mL; 1 mL of electrolyte was extracted for spectroscopic analysis after reaction for each interval.). (c) the absorbance curves of produced NH_3_, and (d) the yield rates and FEs of NH_3_ over the R-RuO_2_/Co(OH)_2_ catalyst in 1 M KOH containing 100 mM NO_3_^−^ with mixed organic and inorganic contaminants. Error bars in Figure b and d represent the standard deviation from three independent measurements.


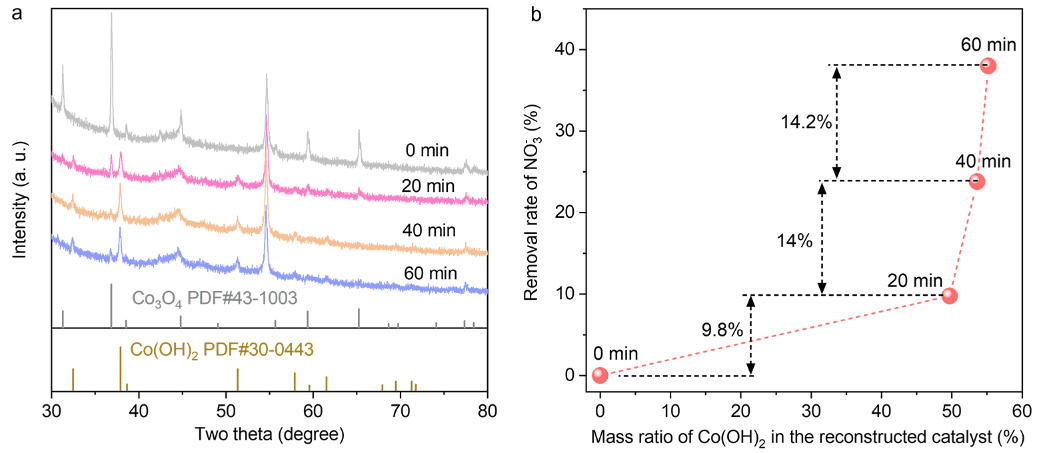


Figure S64. (a) XRD patterns of the Co_3_O_4_ precatalyst after reconstruction at −0.3 V for different time periods. (b) the relationship of removal rate of nitrate and the mass ratio of Co(OH)_2_ in the reconstructed catalysts at varying time scales.


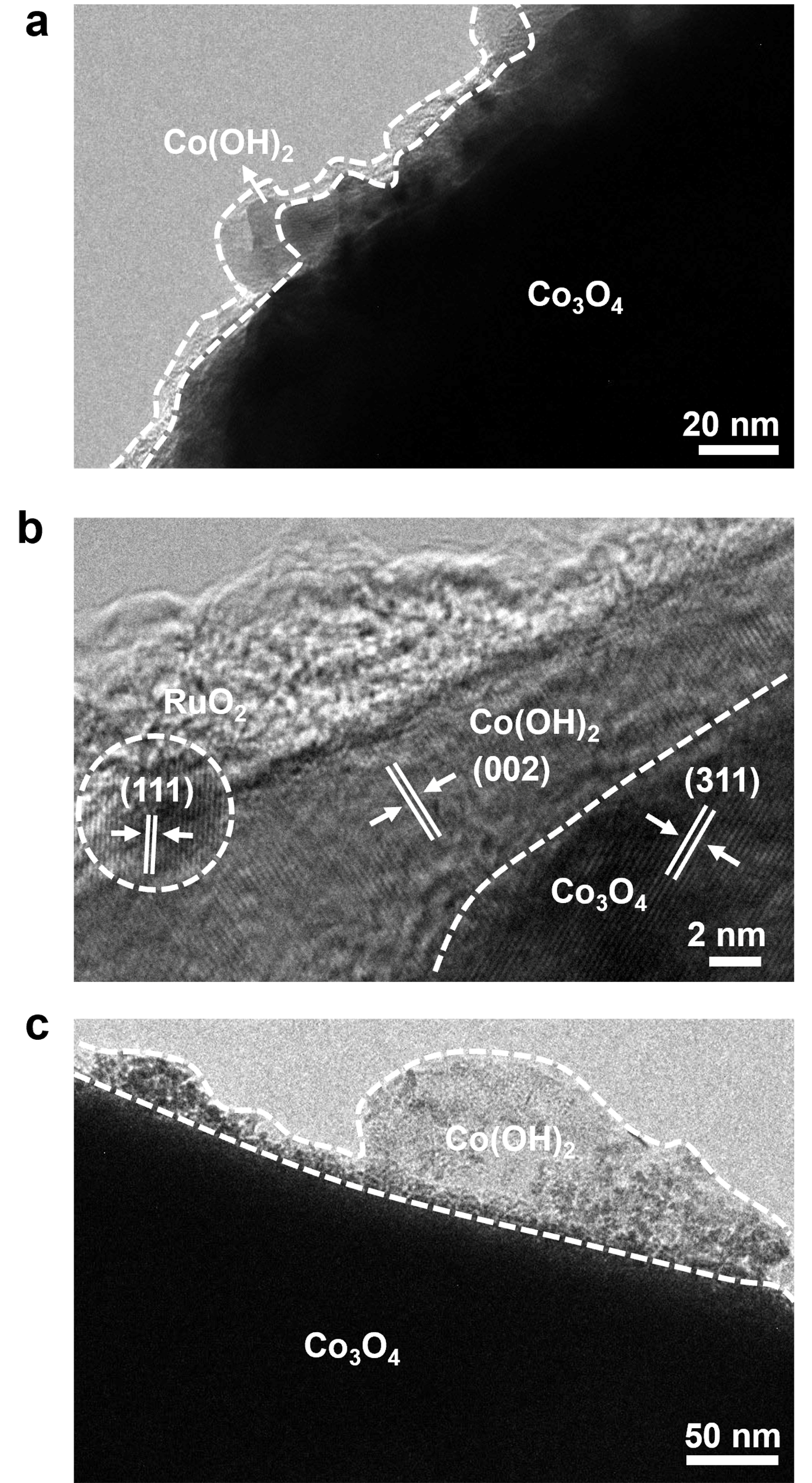


Figure S65. (a, and c) TEM images and (b) HRTEM image of the RuO_2_/Co_3_O_4_-1.1 composite after the reconstruction at −0.3 V vs*.* RHE for 1 hour.

Table S1**.** The Co and Ru content in the as-prepared RuO_2_/Co_3_O_4_ precatalysts determined by ICP-OES results.

| Samples | Co | Ru | Ru/Co ratio |
| --- | --- | --- | --- |
| RuO_2_/Co_3_O_4_-0.3 | 31.627 mg L^−1^ | 0.142 mg L^−1^ | 0.45 wt.% / 0.3 at.% |
| RuO_2_/Co_3_O_4_-1.1 | 3.217 mg L^−1^ | 0.061 mg L^−1^ | 1.9 wt.% / 1.1 at.% |
| RuO_2_/Co_3_O_4_-2.8 | 2.188 mg L^−1^ | 0.106 mg L^−1^ | 4.8 wt.% / 2.8 at.% |

Note: wt.% is the mass ratio of Co versus Ru, while at.% represents the atomic ratio of Co versus Ru.

Table S2. The fitting parameters of Co *K*-edge EXAFS profile.

| Sample | Path | *CN* | S02 | Δ*E*_0_ (eV) | *R*/Å | $\sigma^{2}$/Å^2^ | *R*-factor |
| --- | --- | --- | --- | --- | --- | --- | --- |
| Co foil | Co−Co | 12 | 0.812 | 7.86±0.34 | 2.49 | 0.006 | 0.001 |
| R-Co(OH)_2_ | Co−O | 5.3 |  | 2.01±1.02 | 2.07 | 0.009 | 0.011 |
|  | Co−Co | 5.1 |  |  | 3.16 | 0.007 |  |
| R-RuO_2_/Co(OH)_2_ | Co−O | 5.0 |  | 2.88±1.24 | 2.11 | 0.008 | 0.014 |
|  | Co−Co/Ru | 5.2 |  |  | 3.16 | 0.006 |  |

*CN*: coordination numbers; Δ*E*_0_: the inner potential correction; *R*: bond distance; *σ*^2^: Debye-Waller factors; *R* factor: goodness of fit. Ѕ02 was set to 0.812. For all the EXAFS fitting, the data ranges are presented as follows: 3.0 ≤ *k* ≤ 14.2 Å^−1^, 1 ≤ *R* ≤4 Å.

Table S3. The fitting parameters of Ru *K*-edge EXAFS profile.

| Sample | Path | *CN* | S02 | Δ*E*_0_ (eV) | *R*/Å | $\sigma^{2}$/Å^2^ | *R*-factor |
| --- | --- | --- | --- | --- | --- | --- | --- |
| Ru foil | Ru−Ru | 12 | 0.896 | −4.26±0.89 | 2.68 | 0.004 | 0.008 |
| Com-RuO_2_ | Ru−O | 6 |  | 3.43±1.31 | 1.98 | 0.003 | 0.006 |
|  | Ru−Ru_1_ | 2 |  |  | 3.18 | 0.013 |  |
|  | Ru−Ru_2_ | 8 |  |  | 3.59 | 0.002 |  |
| R-RuO_2_ | Ru−O | 5.7 |  | 5.00±1.54 | 2.02 | 0.005 | 0.017 |
|  | Ru−Ru_1_ | 1.8 |  |  | 3.12 | 0.009 |  |
|  | Ru−Ru_2_ | 7.6 |  |  | 3.52 | 0.004 |  |
| R-RuO_2_/Co(OH)_2_ | Ru−O | 5.6 |  |  | 2.02 | 0.009 | 0.005 |
|  | Ru−Ru_1_/Co | 1.7 |  | 1.64±1.37 | 3.15 | 0.012 |  |
|  | Ru−Ru_2_/Co | 7.4 |  |  | 3.55 | 0.003 |  |

*CN*: coordination numbers; Δ*E*_0_: the inner potential correction; *R*: bond distance; *σ*^2^: Debye-Waller factors; *R* factor: goodness of fit. Ѕ02 was set to 0.896. For all the EXAFS fitting, the data ranges are presented as follows: 3.0 ≤ *k* ≤ 14 Å^−1^, 1 ≤ *R* ≤ 4 Å.

Table S4. Structure and content of different kinds of surface water molecule determined by in-situ Raman spectra at −0.3 V.

| Samples |  | 4-HB-H_2_O | 2-HB-H_2_O | K^+^-H_2_O |
| --- | --- | --- | --- | --- |
| R-Co(OH)_2_ | Peak center (cm^−1^) | 3219.5 | 3418.8 | 3588.8 |
|  | Population (%) | 29.6 | 58.9 | 11.5 |
| R-RuO_2_ | Peak center (cm^−1^) | 3218.8 | 3415.9 | 3578.2 |
|  | Population (%) | 30.6 | 55.7 | 13.7 |
| R-RuO_2_/Co(OH)_2_ | Peak center (cm^−1^) | 3215.6 | 3406.6 | 3567.1 |
|  | Population (%) | 28.3 | 54.7 | 17.0 |

Table S5. The eNO_3_RR performance comparison of the R-RuO_2_/Co(OH)_2_ catalyst with other advanced catalysts.

| **Catalysts** | **Electrolyte** | **Potential**  **(V vs. RHE)** | **Yield rate of NH_3_**  **(mg h**^−^**^1^ cm**^−^**^2^)^a^** | **FE of NH_3_ (%)** | **References** |
| --- | --- | --- | --- | --- | --- |
| RuCo | 0.1 M OH^−^ with 0.1 M NO_3_^−^ | 0 | 54.57 | 97 | Nat. Catal. 2023, 6, 402-414 |
| Co-N-Ru_12_ | 0.1 M OH^−^ with 0.1 M NO_3_^−^ | −0.2 | 15.0 | 90.4 | Nat. Commun. 2024, 15, 8583 |
| Mg_0.2_Co_0.2_Ni_0.2_Cu_0.2_Zn_0.2_O | 1 M OH^−^ with 0.1 M NO_3_^−^ | −0.35 | 4.7 | 93 | Nat. Commun. 2024, 15, 260 |
| CuPd | 1 M OH^−^ with 1 M NO_3_^−^ | −0.5 | 0.02 | 92.5 | Nat. Commun. 2022, 13, 2338 |
| RuCu Das/NGA | 0.1 M OH^−^ with 0.1 M NO_3_^−^ | −0.4 | 3.1 | 95.7 | Nat. Commun. 2025, 16, 2167 |
| Cu/Cu_2_O | 1 M OH^−^ with 0.1 M NO_3_^−^ | −0.2 | 4.25 | 95 | Nat. Commun. 2025, 16, 2392 |
| CuCoSP | 0.1 M OH^−^ with 0.1 M NO_3_^−^ | −0.175 | 19.9 | 93.3 | Nat. Commun. 2022, 13, 1129 |
| Cu_3_N/GDY | 1 M OH^−^ with 0.1 M NO_3_^−^ | −0.9 | 35.28 | 98.1 | J. Am. Chem. Soc. 2024, 146, 14898–14904 |
| (Cu_0.6_Co_0.4_)Co_2_O_4_ | 1 M OH^−^ with 0.1 M NO_3_^−^ | −0.45 | 18.53 | 96.5 | J. Am. Chem. Soc. 2024, 146, 2967–2976 |
| Cu_2_O@CoO | 1 M OH^−^ with 0.1 M NO_3_^−^ | −0.9 | 15.27 | 99 | ACS Nano 2024,18, 20258−20267 |
| RuO_x_/Pd | 1 M OH^−^ with 0.1 M NO_3_^−^ | −0.5 | 23.5 | 98.6 | ACS Nano 2023,17,1081−1090 |
| Ru SA-NC | 1 M OH^−^ with 0.5 M NO_3_^−^ | −0.4 | 11.9 | 72.8 | ACS Nano 2023,17,3483−3491 |
| WN/WO_3_ | 1 M OH^−^ with 0.1 M NO_3_^−^ | −0.7 | 8.4 | 88.9 | ACS Nano 2023,17,25091−25100 |
| (Cu_0.25_Ni_0.25_Fe_0.25_Co_0.25_)Sn_5_ HEI | 1 M OH^−^ with 0.1 M NO_3_^−^ | −0.3 | 7.65 | 98.71 | Adv. Mater. 2025, doi:10.1002/adma.202501886 |
| Cu/Cu_x_O/GDY | 1 M OH^−^ with 0.1 M NO_3_^−^ | −0.8 | 25.4 | 99.8 | Adv. Mater. 2024, 36, 2405660 |
| (Co_0.2_Ni_0.2_Zn_0.2_Mg_0.2_Cu_0.2_)Fe_2_O_4_ | 1 M OH^−^ with 0.1 M NO_3_^−^ | −0.5 | 35.7 | 98.1 | Adv. Mater. 2024, 36, 2403958 |
| Cu_9_S_5_ | 1 M OH^−^ with 0.1 M NO_3_^−^ | −0.3 | 6.29 | 90.4 | Adv. Energy Mater. 2025, 15, 2403354 |
| 5%Ce-Cu/MoO_2_@C | 1 M OH^−^ with 0.1 M NO_3_^−^ | −0.4 | 7.88 | 92 | Adv. Funct. Mater. 2025, 35, 2422025 |
| Cu/Mo-WO_3_ | 0.1 M OH^−^ with 0.1 M NO_3_^−^ | −0.7 | 5.25 | 98.6 | Adv. Funct. Mater. 2025, 35, 2420282 |
| Fe(OH)_2_/Fe@CNTs | 1 M OH^−^ with 0.1 M NO_3_^−^ | −0.4 | 6.29 | 95.1 | Adv. Funct. Mater. 2025, doi:10.1002/adfm.202501079 |
| Cu_1_/Cl-CeO_2_@C | 0.5 M OH^−^ with 0.1 M NO_3_^−^ | −0.5 | 9.528 | 98.8 | Adv. Funct. Mater. 2025, doi:10.1002/adfm.202502073 |
| NF/Ni_3_N-Cu | 1 M OH^−^ with 0.1 M NO_3_^−^ | −0.3 | 20.23 | 98.7 | Angew. Chem. Int. Ed. 2025, 64, e202422585 |
| Cr-Cu_2_O NSAs | 1 M OH^−^ with 0.1 M NO_3_^−^ | −0.175 | 6.81 | 86.65 | Angew. Chem. Int. Ed. 2024, 63, e202411796 |
| Ni_6_@CuFe-LDH | 1 M OH^−^ with 0.1 M NO_3_^−^ | −0.5 | 15.27 | 96.8 | J. Am. Chem. Soc. 2025, 147, 22785–22795 |
| Ag/Co_3_O_4_/CoOOH | 1 M OH^−^ with 0.1 M NO_3_^−^ | −0.25 | 4.31 | 94.3 | Adv. Sci. 2023, 10, 2303789 |
| Cu_2_O/Co_3_O_4_ | 1 M OH^−^ with 0.1 M NO_3_^−^ | −0.3 | 12.76 | 85.4 | Angew. Chem. Int. Ed. 2023, 62, e2022148 |
| Pd- Co_3_O_4_ | 0.1 M OH^−^ with 0.1 M NO_3_^−^ | −0.6 | 12.675 | 98.7 | Small 2023, 19, 2303424 |
| Ov- Co_3_O_4_ | 0.1 M OH^−^ with 0.1 M NO_3_^−^ | −0.5 | 12.157 | 96.9 | Sustainable Energy Fuels 2022, 6, 4130-4136 |
| NiCo_2_O_4_/CC | 0.1 M OH^−^ with 0.1 M NO_3_^−^ | −0.3 | 8.2 | 99 | Small 2022, 18, 2106961 |
| ZnCo_2_O_4_ | 0.1 M OH^−^ with 0.1 M NO_3_^−^ | −0.6 | 8 | 98.33 | Materials today physics 2022, 23, 100619 |
| V_Co_- Co_3_O_4_ | 0.1 M OH^−^ with 0.1 M NO_3_^−^ | −0.4 | 6.55 | 97.2 | ACS Applied Materials & Interfaces, 2022, 14, 46595-46602 |
| **R-RuO_2_/Co(OH)_2_** | **1 M OH^−^ with 0.1 M NO_3_^−^** | **−0.3** | **35.9** | **98.1** | **This work** |

Note: “a” represents the yield rates of NH_3_ at the potential showing maximum FE of NH_3_.

Table S6. Performance comparison of Zn-NO_3_^−^ batteries with various catalysts as cathodes.

| Catalyst | Electrolyte | OCP | Power density (mW cm^−2^) | Yield and FEs of NH_3_ | Stability | Reference |
| --- | --- | --- | --- | --- | --- | --- |
| PdCuAg | 0.1 M KOH +0.01 M NO_3_^−^/6 M KOH | 1.52 | 4.8 | 0.44 mg h^−1^ cm^−2^, 92.4% at 6 mA cm^−2^ | 60 h at 6 mA cm^−2^ | *Adv. Energy. Mater. 2023, 13, 2303054* |
| Fe/Ni_2_P | 0.5 M K_2_SO_4_ + 0.05 M NO_3_^−^/1 M KOH | 1.22 | 3.25 | 0.38 mg h^−1^ cm^−2^, 73% at 10 mA cm^−2^ | 3.5 h at 5 mA cm^−2^ | *Adv. Energy. Mater. 2022, 12, 2103872* |
| Cu-LC-10 | 0.5 M Na_2_SO_4_ + 4500 ppm NO_3_^−^/ 1M KOH | 13 | 3.01 | NA | 9 h at 7 mA cm^−2^ | *Adv. Funct. Mater. 2024, 13, 2315324* |
| 0.6W-o-CoP | 1 M KOH + 0.1 M NO_3_^−^/1 M KOH | 0.7 | 9.27 | 2.38 mg h^−1^ cm^−2^, 75.6% at 40 mA cm^−2^ | NA | *Adv. Mater. 2023, 23044508* |
| Cu-TABQ | 0.5 M K_2_SO_4_ + 50 mM NO_3_^−^/6 M KOH + 0.2 M K(Ac)_2_ | NA | 12.3 | 73% at 35 mA cm^−2^ | 18.5 h at 10 mA cm^−2^ | *Angew. Chem. Int. Ed. 2023, 62, e202309930* |
| Pd/TiO_2_ | 5 M LiCl + 0.25 M NO_3_^−^/ 5 M KOH | 0.81 | 0.87 | 0.48 mg h^−1^ cm^−2^, 81.3% at 7.5 mA cm^−2^ | NA | *Energy Environ. Sci. 2021, 14, 3938* |
| O-CoP/C@Cu_3_P/CF | 1 M KOH + 0.1 M NO_3_^−^/1 M KOH + 0.02 M Zn(Ac)_2_ | 0.78 | 40.1 | 2.42 mg h^−1^ cm^−2^ at 25 mA cm^−2^ | 30 h at 10 mA cm^−2^ | *Energy Environ. Sci. 2024, 17, 4582* |
| CoO/CuO-NA/CF | 0.5 M NaOH + 500 ppm NO_3_^−^/6 M KOH | 1.1 | 4.3 | 0.61 mg h^−1^ cm^−2^, 82% at 8 mA cm^−2^ | 10 h at 0.5 mA cm^−2^ | *Nanoscale, 2023, 15, 19577* |
| RuFe NF | 0.5 M Na_2_SO_4_ + 0.1 M NO_3_^−^/1 M KOH + 0.02 M Zn(Ac)_2_ | 1.37 | 1.9 | NA | 12 h at 0.5 mA cm^−2^ | *PANS 2023, e2306461120* |
| \| **R-RuO_2_/Co(OH)_2_** \| \| --- \| | **1 M KOH + 0.1 M NO_3_^−^/6 M KOH + 0.2 M Zn(Ac)_2_** | **1.44 (89.4% of the theoretical potential)** | **6.44** | **3.40±0.03 mg h^−1^ cm^−2^, 90.7****±0.7% at 50 mA cm^−2^** | **260 h at 1 mA cm^−2^** | ***This work*** |

Table S7. The comparison of Zn-NO_3_^−^ battery with other Zn-based batteries.

| Classification | Theoretical Energy density (Wh kg^−1^) | Practical Energy density (Wh kg^−1^) | Cost analysis | | Safety and sustainability impact | Advantages | Reference |
| --- | --- | --- | --- | --- | --- | --- | --- |
|  |  |  | System cost | Maintenance cost |  |  |  |
| Zn-NO_3_^−^ battery | 1051 | 679.5 | Low materials cost and moderate cost of the device assembly | Low (aqueous electrolytes) | High safety (no thermal runaway risk), and high sustainability (achieving the elimination of nitrogen-containing pollutants, NH_3_ synthesis and energy supply) | High energy density, low cost and environmentally friendly, suitable for coupling of wastewater treatment with distributed energy | *Cell Reports Physical Science* 2025, 6, 102606 |
| Zn-air  battery | 1350 | 1086 | Low materials cost but high cost of the device assembly | Moderate (waterproof management) | High safety (no thermal runaway risk), and high sustainability (need O_2_ supply) | Highest theoretical energy density | *Small* 2018, 14, 18019292;  *Small Sci*. 2024, 4, 2300094 |
| Zn-I_2_  battery | ~585.4 | 10-171.3 | Moderate materials cost and low cost of the device assembly | Low | Moderate safety, and moderate sustainability (Toxic I_2_ gas, hard recycling of I_2_) | Long lifetime | *Adv. Energy Mater.* 2025, 15, 2404426;  *J. Am. Chem. Soc.* 2025, 147, 16350−163614;  *Nano Energy* 2025, 138, 110884 |
| Zn-Br_2_ battery | 440 | 60-75 | Moderate materials cost and low cost of the device assembly | Low | Low safety, and low sustainability (easy leakage of Br_2_, toxic) | Long lifetime | *Adv. Mater.* 2022, 34, 2108856;  *Energy Storage Materials* 2023, 61, 102894;  *Adv. Mater.* 2022, 34, 2108856 |
| Zn-Mn battery | 135 | 100-110 | Low materials cost and low cost of the device assembly | Moderate (refilling electrolytes) | High safety (no thermal runaway risk), and high sustainability | Mature technology | *Adv. Mater.* 2005, 17, 2753–2756;  *Nano Lett.* 2021, 21, 1446−1453 |
| Zn-Ni  battery | 200-350 | 172.8 | Moderate materials cost and moderate cost of the device assembly | High (system maintenance regularly) | Moderate safety and moderate sustainability (hard recycling of Ni) | Application for extreme environment | *Adv. Mater.* 2016, 28, 8732–8739 |

Table S8. Performance comparison of collecting ammonia products by the argon stripping and acid trapping method.

| **Catalysts** | **Removed NH_3_ from reacted electrolytes** | **Acid trapped NH_3_** | **Collected NH_4_Cl(s)/NH_4_NO_3_ (s)** | **Residual NH_4_^+^ in reacted electrolytes** | **References** |
| --- | --- | --- | --- | --- | --- |
| CoW/CF | 99.4% | NA | 97.9% | NA | *Adv. Energy Mater.* 2024, 14, 2303321 |
| MAT-CoNi/CF | 90.29% | NA | 90.89% | NA | *Adv. Mater*. 2024, 36, 2404774 |
| Pd-Cl/Cu_2_O | 95.2% | 93.1% | 80.5% | NA | *Nat. Commun*. 2024, 15, 1264 |
| Ni(OH)_x_/Cu | NA | NA | 69.8% | NA | *Nat. Commun*. 2024, 15, 3524 |
| Ru-CuNW | 99.7% | 97% | ~85% | 0.55 ppm | *Nat. Nanotechnol.* 2022, 17, 759-767 |
| R-RuO_2_/Co(OH)_2_ | 99±1% | 97.7±0.3% | 95.2% | 11.2 ppm (48 h);  1.03 ppm (96 h) | **This work** |

# References

[1] X. Zhang, X. Liu, Z.-F. Huang, L. Guo, L. Gan, S. Zhang, M. Ajmal, L. Pan, C. Shi, X. Zhang, G. Yang, J.-J. Zou, *ACS Catal.* **2023**, *13*, 14670-14679.

[2] S. Han, H. Li, T. Li, F. Chen, R. Yang, Y. Yu, B. Zhang, *Nat. Catal.* **2023**, *6*, 402-414.

[3] X. Kong, G. Liu, S. Tian, S. Bu, Q. Gao, B. Liu, C. S. Lee, P. Wang, W. Zhang, *Small* **2022**, *18*, e2204615.

[4] R. Zhang, Y. Guo, S. Zhang, D. Chen, Y. Zhao, Z. Huang, L. Ma, P. Li, Q. Yang, G. Liang, C. Zhi, *Adv. Energy Mater.* **2022**, *12*, 2103872.

[5] a) W. Yu, J. Yu, M. Huang, Y. Wang, Y. Wang, J. Li, H. Liu, W. Zhou, *Energy Environ. Sci.* **2023**, *16*, 2991-3001; b) Y. Lee, J. Theerthagiri, N. Yodsin, A. Min, C. J. Moon, S. Jungsuttiwong, M. Y. Choi, *Angew. Chem. Int. Ed.* **2024**, *63*, e202413774

[6] W. Yu, Y. Wang, H. Tan, M. Huang, J. Yu, L. Chen, J. Wang, H. Liu, W. Zhou, *Adv. Energy Mater.* **2024**, *14*, 2402970.

[7] J. Hafner, *J. Comput. Chem.* **2008**, *29*, 2044-2078.

[8] P. E. Blochl, *Phys. Rev. B.* **1994**, *50*, 17953-17979.

[9] J. P. Perdew, Burke, K., Ernzerhof, M., *Phys. Rev. Lett.* **1996**, *77*, 3865-3868.

[10] B. Hummer, Hansen, L. B., Norskov, J. K. , *Phys. Rev. B* **1999**, *59*, 7413-7421.

[11] W. Kohn, Sham, L. J., *Phys. Rev.* **1965**, *140*, A1133-A1138.

[12] G. Kresse, Furthmuller, J. , *Phys. Rev. B* **1996**, *57*, 11169-11184.

[13] S. L. Dudarev, Botton, G. A., Savrasov, S. Y., Humphreys, C. J., Sutton, A. P. , *Phys. Rev. B* **1998**, *57*, 1505-1509.

[14] A. Jain, S. P. Ong, G. Hautier, W. Chen, W. D. Richards, S. Dacek, S. Cholia, D. Gunter, D. Skinner, G. Ceder, K. A. Persson, *APL Mater.* **2013**, *1*, 011002.

[15] A. Hjorth Larsen, J. Jorgen Mortensen, J. Blomqvist, I. E. Castelli, R. Christensen, M. Dulak, J. Friis, M. N. Groves, B. Hammer, C. Hargus, E. D. Hermes, P. C. Jennings, P. Bjerre Jensen, J. Kermode, J. R. Kitchin, E. Leonhard Kolsbjerg, J. Kubal, K. Kaasbjerg, S. Lysgaard, J. Bergmann Maronsson, T. Maxson, T. Olsen, L. Pastewka, A. Peterson, C. Rostgaard, J. Schiotz, O. Schutt, M. Strange, K. S. Thygesen, T. Vegge, L. Vilhelmsen, M. Walter, Z. Zeng, K. W. Jacobsen, *J. Phys. Condens. Matter.* **2017**, *29*, 273002.

[16] J. K. Nørskov, Rossmeisl, J., Logadottir, A., Lindqvist, L. , *J. Phys. Chem. B* **2004**, *108*, 17886-17892.

[17] M. Bajdich, M. Garcia-Mota, A. Vojvodic, J. K. Norskov, A. T. Bell, *J. Am. Chem. Soc.* **2013**, *135*, 13521-13530.

[18] L. Qiao, D. Liu, A. Zhu, J. Feng, P. Zhou, C. Liu, K. W. Ng, H. Pan, *Appl. Catal. B* **2024**, *340*, 123219.

[19] J. A. Koza, C. M. Hull, Y.-C. Liu, J. A. Switzer, *Chem. Mater.* **2013**, *25*, 1922-1926.

[20] S. Liang, X. Teng, H. Xu, L. Chen, J. Shi, *Angew. Chem. Int. Ed.* **2024**, 63， e202400206.
